# Fluorine-free polymer binder enables circular manufacturing and recycling of lithium-ion batteries

**DOI:** 10.1038/s41467-026-78215-3

**Published:** 2026-10-09

**Authors:** Jiao Lin, Anthony Mu, Wei Tang, Feng Li, Yuwei Zhu, Mingqian Li, Ram Hemanth Yeerella, Christopher Potts, Shengqiang Cai, Linqin Mu, John Muldoon, Zheng Chen

**Affiliations:** 1 Aiiso Yufeng Li Family Department of Chemical and Nano Engineering, University of California San Diego, La Jolla, CA USA; 2 Materials Science and Engineering, School for Engineering of Matter, Transport and Energy, Arizona State University, Tempe, AZ USA; 3 Department of Mechanical and Aerospace Engineering, University of California San Diego, La Jolla, CA USA; 4 Program in Materials Science and Engineering, University of California San Diego, 9500 Gilman Dr, La Jolla, CA USA; 5 Toyota Research Institute of North America, 1555 Woodridge Avenue, Ann Arbor, MI USA; 6 Sustainable Power and Energy Center, University of California San Diego, La Jolla, CA USA

**Keywords:** Batteries, Environmental impact, Sustainability, Batteries

## Abstract

Today’s lithium‑ion battery manufacturing heavily relies on polyvinylidene fluoride binder and N‑methyl‑2‑pyrrolidone solvent-based processing, which incurs high cost and hinders environmental sustainability. While bio-derived binders enable aqueous processing, their reliance on hydrogen bonding alone limits mechanical strength and electrochemical stability. Here, we introduce a fluorine‑free, water‑processable binder comprised of polyacrylic acid and carboxymethyl cellulose crosslinked by citric acid. The network integrates hydrogen, covalent, and ionic bonding among binder chains, active particles, and current collectors, providing strong adhesion as well as good oxidative stability and thermal stability. LiFePO_4_ positive electrodes fabricated with fluorine‑free binder deliver comparable initial capacity to the control electrodes with polyvinylidene fluoride binder, while providing better cycle stability. This chemistry applies to a wide range of positive and negative electrode materials. Importantly, the fluorine‑free binder further facilitates sustainable direct recycling, demonstrating full recovery of spent positive and negative electrodes to achieve the same level of performance with pristine materials without generating fluorinated residue. Life-cycle analysis shows significant reductions in energy cost and emissions compared with recycling polyvinylidene fluoride-based batteries. This binder strategy endows high performance with circularity, offering a scalable route to sustainable battery manufacturing technologies.

## Abstract (web summary)

Conventional polymer binders rely on fossil-derived chemicals and toxic solvents. Here, the authors develop a renewable citric acid-crosslinked polymer binder that enables durable lithium-ion batteries, improves electrode integrity, and supports sustainable battery manufacturing and recycling.

## Introduction

Transitioning to large‑scale electrification requires battery manufacturing to combine high performance with safety, resource‑efficiency and recyclability^1^. Although binders account for a relatively small mass fraction of an entire battery, their chemistry dictates the choice of processing solvent, cell structure stability and end‑of‑life (EoL) strategies^2^. Polyvinylidene fluoride (PVDF) provides good chemical stability and mechanical cohesion but requires fluorinated feedstocks with toxic N‑methyl‑2‑pyrrolidone (NMP) solvent, complicating factory environmental, health, and safety controls^3–6^. Moreover, PVDF residues are difficult to separate from active materials and require high-temperature removal, generating hazardous byproducts that threaten environmental and human health^7^. These drawbacks drive up manufacturing costs, complicate recycling, and motivate the urgent replacement of fluoropolymers with more sustainable alternatives^8,9^.

Water-processable binders such as carboxymethyl cellulose (CMC) and polyacrylic acid (PAA) offer renewable and biodegradable options that eliminate NMP, reduce drying energy, and lower capital costs^10^. Already manufactured at scale for packaging and coatings, these polymers have been commercialized in graphite negative electrodes and are being explored for positive electrodes manufacturing. Techno-economic analyses show that aqueous processing can halve capital requirements compared with organic solvent-based systems by simplifying drying and obviating solvent recovery, while also lowering environmental burden^11^. However, aqueous binder films often crack during drying, forming brittle electrodes. At high voltages, repeated volume changes generate mechanical stress, and limited binder elasticity risks delamination and capacity fade^12^.

**Fig. 1:**
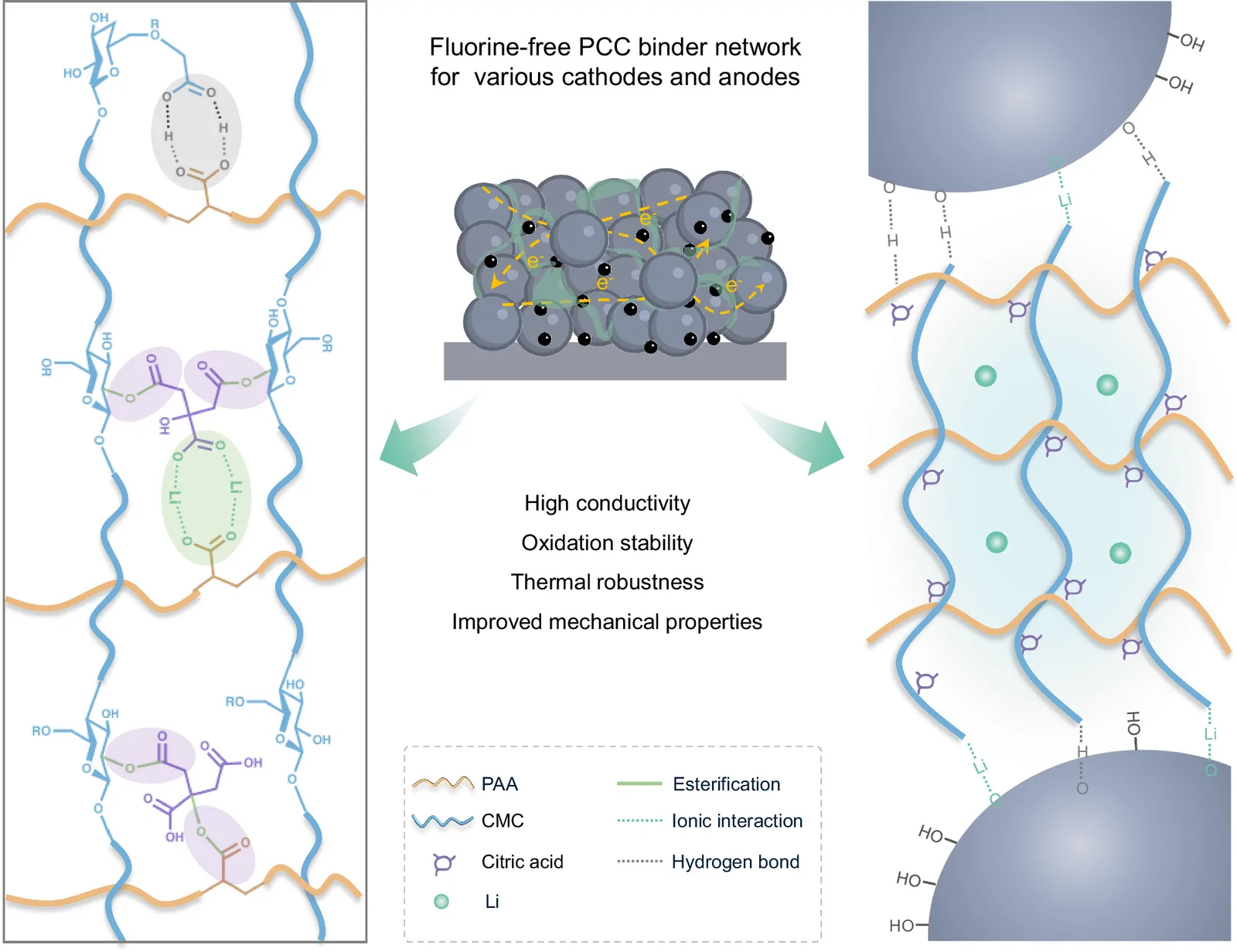
Schematic illustration of the poly(acrylic acid)/carboxymethyl cellulose/citric acid (PCC) binder network and its multifunctional compatibility with diverse electrode chemistries. Poly(acrylic acid) (PAA) and carboxymethyl cellulose (CMC) chains form a dynamic network stabilized by esterification reactions, ionic crosslinking, and hydrogen bonding with citric acid (CA), and Li^+^. The incorporation of CA introduces abundant carboxyl and hydroxyl groups, enhancing interfacial adhesion and structural robustness. The resulting hydrogel-like PCC matrix exhibits strong binding and mechanical integrity, enabling universal applications across a wide range of electrodes.

Herein, we introduce a fluorine (F)-free polymer network composed of crosslinked PAA and CMC (denoted as PCC) for aqueous electrode manufacturing. Citric acid (CA) acts as a multifunctional crosslinker, forming ester bridges with both polymers and reinforcing hydrogen-bond networks to strengthen interfacial cohesion. The resulting PCC binder forms a robust, stress-dissipative network that maintains intimate electrical contact between active materials and conductive carbon, supporting structural stability during long-term cycling (Fig. 1). In LiFePO_4_ (LFP) positive electrodes, it simplifies binder removal for efficient material recovery. This scalable and environmentally benign binder platform enhances both performance and recyclability, advancing sustainable LIB production.

## Results

### Design and properties of PCC binder

A desired water-processable binder must simultaneously satisfy mechanical, electrochemical, and recyclability requirements. The PCC binder is designed to integrate these functions within a single, F-free aqueous system. PAA provides abundant carboxyl groups that form hydrogen bonds and ester linkages. In mildly neutralized aqueous conditions, PAA exhibits high segmental mobility that facilitates uniform film formation and processability^5,13^. In contrast, CMC contributes rigidity and cohesive strength through hydroxyl- and ether-mediated bridging between active materials and conductive carbon^14^. Yet each polymer alone has intrinsic shortcomings: in the dry state, PAA forms glassy, moisture-sensitive films with limited toughness and unstable adhesion, whereas CMC lacks elasticity and exhibits poor oxidative stability on high-voltage operation due to the underlying polysaccharide backbone and carboxymethyl groups (-CH_2_COOH)^13^. Common modifications (e.g., CMC/styrene-butadiene rubber blends or crosslinker addition within a single-binder system) yield only modest gains because the interactions remain weak and reversible, and single-polymer chemistries impose inherent constraints^15^.

To overcome these limitations, we couple the processability of PAA with the cohesion strength of CMC while suppressing oxidative degradation pathways. Direct coupling between PAA and CMC is hindered by steric hindrance and chain entanglement, and traditional elevated-temperature crosslinking (≥150 °C) risks polymer degradation and loss of processability^16,17^. We therefore introduce CA as a small molecule crosslinker that is capable of covalently crosslinking the two polymers under mild aqueous conditions (Supplementary Table 1, Supplementary Note 1). The solution pH is adjusted to ≈6 to preserve positive electrodes integrity and prevent aluminum current collector corrosion^18^. At such conditions, PAA (p*K*_a_ = 4.5^19^) and CMC (p*K*_a_ = 4.76^20^) are partially deprotonated, enabling esterification and Li^+^-mediated ionic coordination. With its hydroxyl and carboxyl groups, CA mediates multiple interactions: covalent ester linkages with PAA and CMC, hydrogen bonding among polymer chains, and Li⁺-bridged ionic associations. The resulting covalent-ionic-hydrogen-bonded hybrid PCC network consumes free -OH and -COOH groups, thereby lowering the density of oxidizable sites and confining residual polar groups within a dense matrix^21,22^, which enhances oxidative stability while retaining compatibility with aqueous processing (Fig. 1)^23^.

**Fig. 2:**
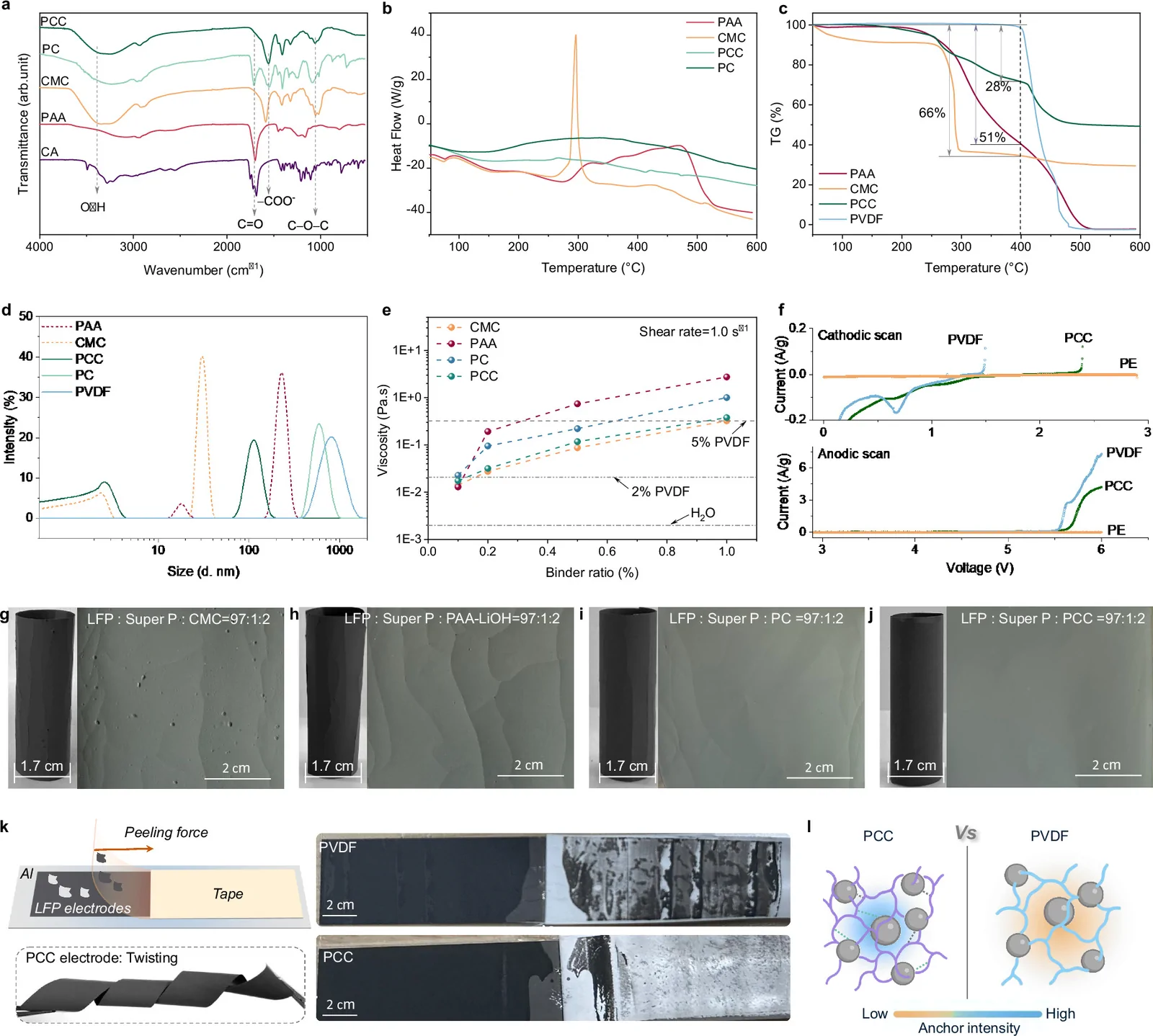
Characterizations of the PCC binder system. **a** Fourier transform infrared (FTIR) spectra of PAA, CMC, CA, and various mixtures. **b** Differential scanning calorimetry (DSC) profile of pristine PAA powder, pristine CMC powder, PAA-CMC-LiOH mixture (PC), and PCC powder. **c** Thermogravimetric analysis (TGA) thermograms of PAA, CMC, PCC, and polyvinylidene fluoride (PVDF). **d** Size distributions of PAA, CMC, PC, PCC, and PVDF/N-methyl-2-pyrrolidone (NMP) solutions. All water-processable binder solutions were prepared at 1 wt%, whereas the PVDF solution was prepared at 0.2 wt% because its large particle size resulted in poor dispersion at higher concentrations. **e** Viscosity comparison of various binder solutions (CMC, PAA, PC, PCC) at different concentrations (wt%). Data were extracted from a shear rate of 1 s^−1^ for comparison. Dashed lines mark the viscosity range of conventional 2 wt% and 5 wt% PVDF solutions in NMP, as well as pure water, for comparison. Complete flow curves over a shear-rate of 0.1–15 s^−1^ are provided in Supplementary Fig. 5. **f** Linear sweep voltammetry of PVDF- and PCC-based electrodes, with pure electrolyte (PE) included as a control, under anodic and cathodic scans (25 ± 2 °C). **g**, **j** Digital photographs of LiFePO_4_ (LFP) electrodes prepared with different binders: (**g**) CMC, (**h**) PAA-LiOH, (**i**) PC, and (**j**) PCC. All electrodes were prepared with a fixed composition of LFP: Super P: Binder = 97: 1: 2 by weight and cast on carbon-coated Al foil under identical conditions. **k** Schematic and photographic illustration of tape-peeling tests comparing PVDF and PCC electrodes, alongside a twisting test for PCC electrodes. The two left panels show the initial peel. **l** Schematic comparison of the polymer-particle interactions in PCC and PVDF. The color gradient denotes relative anchoring strength. Source data are provided as a Source Data file.

Fourier-transform infrared spectroscopy confirms the aforementioned interactions. Compared with pristine CMC, the CMC and 5 wt% CA (CMC-CA) system shows weakened O-H stretching (~3400 cm⁻¹) and the emergence of a new peak near 1700 cm⁻^1^ indicates ester formation^24^, accompanied by enhanced carboxylate stretching (~1600 and ~1400 cm⁻¹) and R-O-R’ vibrations (~1050–1150 cm⁻¹, Supplementary Fig. 1a). Similar features appear in the PAA and 5 wt% CA (PAA-CA, pH adjusted to ~6) system, where intensified C-O stretching (~1200–1250 cm⁻¹) and a new ester peak at ~1700 cm⁻^1^ confirm covalent bonding between PAA and CA^25^. A shoulder near 1550 cm⁻^1^ corresponding to lithium-carboxylate coordination, indicating Li⁺-mediated ionic bridges within the network (Supplementary Fig. 1b). Pronounced splitting of carbonyl bonds and broadening of the O-H stretching in crosslinked PCC indicate an ester-Li^+^-carboxylate network, consistent with enhanced oxidative stability (Fig. 2a).

Thermogravimetric analysis (TGA) further supports this multi-interaction framework (Supplementary Fig. 2, Supplementary Table 2). A left-shifted decomposition profile is consistent with ester-containing networks^26^, while differential scanning calorimetry (DSC) reveals a low-temperature exotherm upon LiOH incorporation relative to PAA, indicative of lithium-carboxylate formation. Electrolyte uptake, galvanostatic intermittent titration technique (GITT) analysis, and cycling stability measurements (Supplementary Fig. 3) further reveal how crosslink density influences electrochemical behavior. Increasing CA content systematically reduces binder swelling by increasing the density of esterification-induced covalent crosslinks within the polymer network (Supplementary Fig. 3b). The tighter network restricts polymer chain mobility and reduces free volume, thereby suppressing solvent absorption and electrolyte uptake. While this compact structure enhances mechanical cohesion and interfacial stability, excessive crosslinking can hinder electrolyte infiltration and limit Li^+^ accessibility within the electrode pores. This effect is reflected in the GITT results, where highly crosslinked binders exhibit lower apparent Li⁺ diffusion coefficients, indicating additional transport resistance within the electrode matrix (Supplementary Fig. 3c). The optimal composition, a PAA:CMC ratio of 1:1 with 5–8 wt% CA, achieves a balanced network, providing sufficient crosslink density for mechanical reinforcement without excessive thermal degradation (Fig. 2b, Supplementary Note 1, and Supplementary Tables 3, 4)^24^. At this optimized ratio, the hybrid ionic-covalent network yields delayed thermal decomposition (Fig. 2c), suggesting enhanced thermal durability.

The colloidal behavior and rheological characteristics of PCC elucidate its favorable processability. Dynamic light scattering (DLS) reveals distinct size distributions: CMC disperses into the smallest particles (~20–40 nm), whereas PAA forms larger aggregates (~200–300 nm). The binary PAA-CMC-LiOH (PC) system yields broad distributions (~500–900 nm), indicating random aggregation in aqueous media. Incorporation of CA and LiOH strengthens PAA-CMC associations, yielding smaller, uniform particles (~100–200 nm). In contrast, PVDF in NMP forms broad, multimodal distributions extending into the micron size range (~800–1200 nm), symptomatic of gel-like clusters rather than discrete nanoparticles (Fig. 2d). Importantly, PCC dispersions remain stable across pH 4–6 with negligible shifts (<10 nm) in hydrodynamic size, confirming their robust colloidal stability (Supplementary Fig. 4a).

Furthermore, comparison among alternative acids indicates that CA yields the most uniform dispersions. The CA-based PCC shows narrow distributions, whereas boric acid, oxalic acid, and L-ascorbic acid produce distinctly broadened or multimodal distributions (Supplementary Fig. 4b). This behavior is attributed to the trifunctional topology and mixed carboxylate-hydroxyl chemistry of CA, which supports balanced esterification of both PAA and CMC at pH ≈ 6 to form a dense yet colloidally stable ionic network without the need of heat treatment. Increasing the PCC binder concentration form 0.1 to 1 wt% results in a more than one-order-of-magnitude viscosity increase (from ~1.7 × 10^−2^ Pa·s to ~0.38 Pa·s), indicative of progressive network formation while maintaining colloidal uniformity and optical clarity (Fig. 2e and Supplementary Fig. 5).

Electrochemical stability was evaluated using linear sweep voltammetry (LSV). The PCC binder demonstrates an oxidative onset exceeding 5.0 V vs. Li/Li⁺ and minimal parasitic currents under both cathodic and anodic conditions (Fig. 2f). To benchmark the PCC binder, we systematically compared LFP electrodes fabricated with PCC against those using a commercial PVDF binder. Morphological analyses reveal that binder rheology and structural stability during solvent evaporation govern electrode integrity. Electrodes fabricated using CMC, CMC-CA, PAA-LiOH, PAA-LiOH-CA, or PC exhibit pronounced cracks, voids, and delamination upon drying (Fig. 2g–i and Supplementary Fig. 6). In contrast, PCC yields smooth, defect-free coatings without shrinkage or interfacial disruption (Fig. 2j). Mechanical tests further confirm its robustness: negligible active material detachment was observed after repeated tape-peeling or twisting, whereas PVDF electrodes suffer severe materials loss after only a few trials (Fig. 2k and Supplementary Fig. 7). This stronger adhesion in PCC originates from a Li-carboxylate-ester network that delivers multipoint anchoring^13^, whereas PVDF relies solely on weak van der Waals interactions^27^ (Fig. 2l). At the atomic scale, Fe K-edge X-ray absorption near-edge structure (XANES) and Fourier-transformed extended X-ray absorption fine structure (EXAFS) analysis confirm that PCC processing does not perturb the olivine structure or Fe^2+^ environment. Both PCC- and PVDF-based electrodes display identical absorption edges near ~7125 eV and similar radial distribution profiles, indicating that the aqueous PCC system preserves the original coordination environment of LFP (Supplementary Fig. 8).

Collectively, these results establish PCC as a promising F-free binder that combines mechanical resilience, thermal and electrochemical stability, and full aqueous processability, offering a sustainable and scalable alternative to PVDF-based systems.

### Electrochemical properties of PCC-aqueous-based binder electrodes

**Fig. 3:**
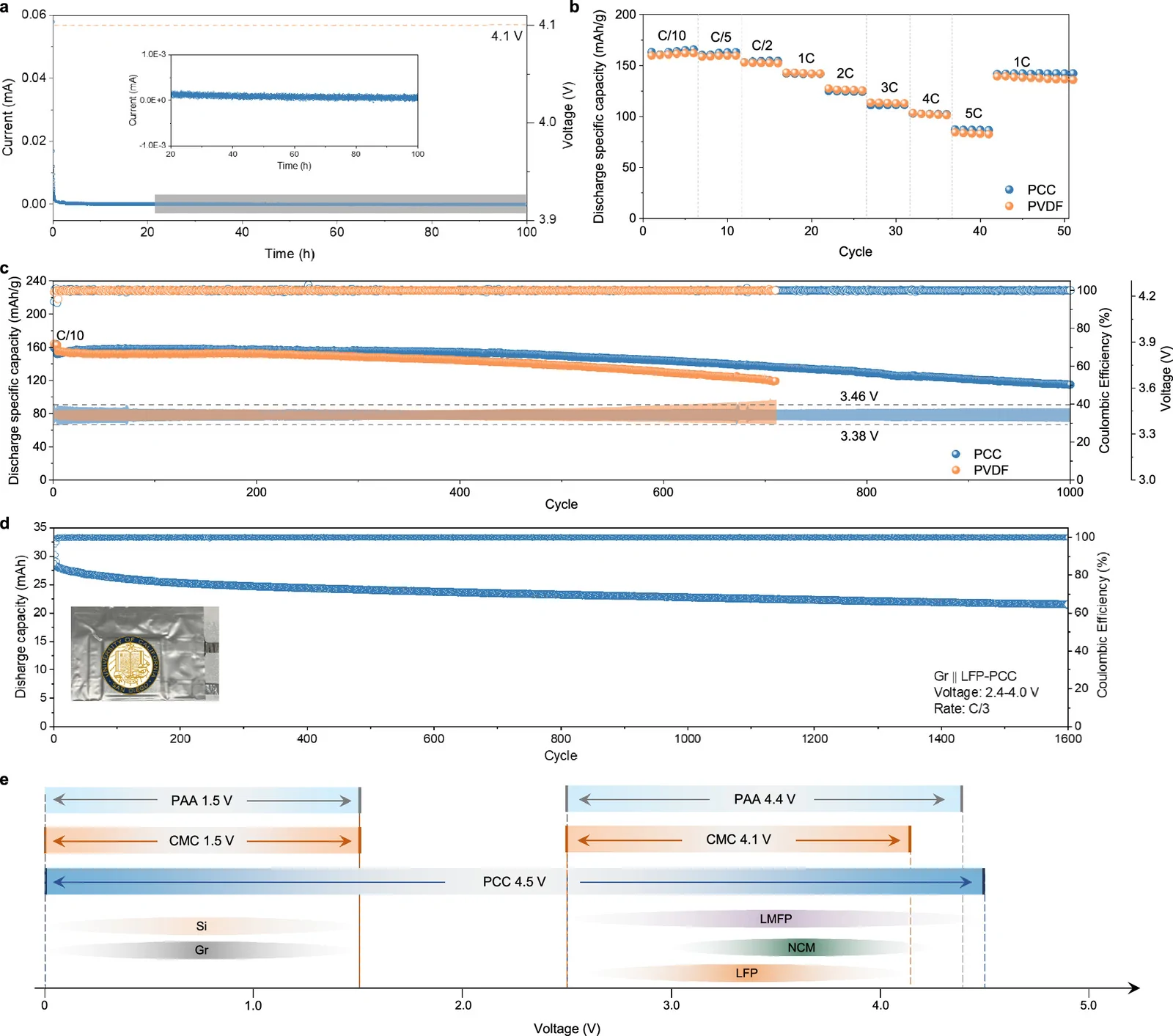
PCC binder enables uniform coatings, strong adhesion, and stable electrochemical performance. **a** Current profiles of Li | |LFP half-cells during a 4.1 V float-charge test (25 ± 2 °C). **b** Rate capability of coin cells employing LFP-PCC and LFP-PVDF positive electrodes (1 C = 170 mA g^−1^, 25 ± 2 °C). **c** Cycling performance and polarization profiles of Li | |LFP half coin-cells employing LFP-PCC and LFP-PVDF positive electrodes cycled between 2.7 and 4.1 V versus Li/Li⁺ after formation at C/10 (1 C = 170 mA g^−1^, 25 ± 2 °C). **d** Cycling performance of a single-layer graphite (Gr)||LFP-PCC pouch cell operated at C/3 (1 C = 150 mA g^−1^, 25 ± 2 °C). **e** Schematic illustrating the applicability of the PCC binder to silicon (Si), graphite, LFP, Ni-rich layered oxide, and LiMn_0.6_Fe_0.4_PO_4_ (LMFP) electrodes. Source data are provided as a Source Data file.

We employed LFP as a model positive electrode material to investigate the electrochemical properties of the PCC binder system. The PCC-based LFP electrode exhibits a near-zero leakage current during a 100-h floating test at 4.1 V, demonstrating good interfacial passivation and electrochemical stability (Fig. 3a). Electrochemical impedance spectroscopy (EIS) combined with distribution of relaxation time (DRT) analysis reveals that LFP-PCC electrodes maintain lower charge-transfer resistance and cathode electrolyte interphase resistance than PVDF counterparts throughout cycling (Supplementary Figs. 9, 10 and Supplementary Table 5)^28^. The PCC electrode exhibits stable impedance evolution and minimal interfacial buildup, consistent with enhanced Li^+^ transport. Complementary conductivity measurements of LFP: binder (98:2, w/w) composites show that PCC preserves the electronic conductivity of pristine (pLFP) more effectively than PVDF (41 vs 24 mS cm⁻^1^; Supplementary Fig. 11, 12), reflecting improved particle bridging through hydrogen-bonded and ionically coordinated networks.

The electrochemical performance of LFP-PCC and LFP-PVDF electrodes was further benchmarked in Li | |LFP half-cells. Both electrodes exhibit the characteristic flat voltage plateaus and sharp dQ/dV peaks associated with the Fe^2+^/Fe^3+^ redox couple in LFP (Supplementary Fig. 13). LFP-PCC positive electrode delivers an initial discharge capacity of 160.2 mAh g^−1^ and initial Coulombic efficiency of 96.8%, comparable to the PVDF-based positive electrode (160.5 mAh g^−1^ and 95.7%, Supplementary Fig. 13b). Rate capability tests show that LFP-PCC positive electrodes exhibit comparable capacity retention across all rates (Fig. 3b) with higher capacity recovery when returning to 1 C (1 C = 170 mA g^−1^) relative to PVDF. Extended cycling of LFP-PCC at C/3 retains 114.9 mAh g⁻^1^ after 1000 cycles (Fig. 3c), corresponding to 75.7% capacity retention, whereas PVDF electrodes undergo faster capacity fade, particularly after 400 cycles. A slight initial capacity increase observed for PCC-based electrode may be attributed to Li reservoirs present within the binder network, providing additional active Li^+^ in early cycles^15,29^. This feature likely contributes to the marginally larger initial charge-discharge voltage gap (ΔV) of PCC-based electrodes compared with PVDF. Upon cycling, ΔV of PCC quickly decreases and stabilizes, suggesting improved interfacial compatibility and reduced polarization (Fig. 3c). After prolonged cycling, PCC electrodes retain low-impedance and narrow relaxation-time distributions, whereas PVDF shows broadened mid-frequency features and increased interfacial resistance (Supplementary Figs. 14–16 and Supplementary Table 5). Correlative post-mortem transmission electron microscopy-energy dispersive spectroscopy (TEM-EDS) reveals that PCC maintains well-resolved crystalline fringes capped by a thin (~2-10 nm) uniform interphase with minimal F signal. In contrast, PVDF forms an inhomogeneous, F-rich layer (~5-50 nm) with lattice distortion and particle edge roughening (Supplementary Figs. 17, 18). PVDF forms a fluorine- and salt-rich sub-surface interphase that persists upon sputtering, whereas PCC exhibits a more readily removable interphase with weaker subsurface salt accumulation (Supplementary Figs. 19). These observations are consistent with prior evidence that PVDF/LiPF_6_ systems yield thicker F-rich interphases, while aqueous binders promote more uniform, thinner passivation layers^30,31^.

We extended evaluation across multiple electrode chemistries, including LiMn_0.6_Fe_0.4_PO_4_ (LMFP), Ni-rich (LiNi_0.83_Co_0.11_Mn_0.06_O_2_, NCM811), graphite (Gr), and silicon (Si). In Li | |LMFP half-cell configuration, the LMFP-PCC electrode delivers a stable discharge capacity of ~123.7 mAh g^−1^ at a C/3 rate over 590 cycles, corresponding to slow capacity decay of 0.01 % per cycle (Supplementary Fig. 20). At 42 °C, it exhibits an initial discharge capacity of 144.0 mAh g^−1^ with 91.2% capacity retention after 180 cycles (Supplementary Fig. 21). These results underscore the compatibility of the PCC binder with high-voltage phosphate positive electrodes (up to 4.5 V) and its robustness under elevated-temperature operation. For Ni-rich materials, PCC binder enables robust dispersion and suspension of NCM811 particles, effectively suppressing settling and time-dependent instability (Supplementary Fig. 22). Time-dependent rheological measurements show that the NCM811-PCC slurry maintains stable viscosity and shear-thinning behavior over 0–24 h without the abrupt viscosity increase characteristic of slurry gelation (Supplementary Fig. 22a), indicating a stable particle-binder network during storage and casting. In contrast, PVDF-based NCM811 slurries exhibit a pronounced viscosity decrease with holding time and severe sedimentation after 48 h of static storage, whereas the PCC slurry remains homogeneous without observable phase separation (Supplementary Fig. 22b, c). Ni-rich NCM811 positive electrodes fabricated with PCC exhibit the characteristic H_1_ → M → H_2_ → H_3_ phase transitions along with improved capacity retention compared to its PVDF analog (Supplementary Fig. 23). On the negative electrode side, PCC enables stable cycling of both graphite and silicon. Gr-PCC electrodes show narrower charge-discharge voltage gaps compared to those with PVDF (Supplementary Fig. 24a), revealing improved lithiation/delithiation kinetics and enhanced capacity retention. Li | |Si half cells employing the PCC binder exhibit robust cycling stability, retaining over 3000 mAh g^−1^ after 100 cycles at C/3 with a stable 0.22 V polarization (Supplementary Fig. 24b).

Finally, the practicality of PCC was validated in single- and multi-layer Gr | |LFP pouch cells, where the binder ensured uniform coating, mechanical flexibility, and defect-free interfaces across large-area electrodes (Supplementary Fig. 25). Single layer cell cycling at C/3 rate demonstrated good electrochemical stability, retaining 76.4% of its initial capacity over 1600 cycles (Fig. 3d). The mechanical robustness of PCC also enabled straightforward assembly of multi-layer pouch cells, whose electrodes maintained integrity under practical handling. The multi-layer pouch delivered slightly lower capacity retention (74.5% of 700 cycles vs 83.5% of 700 cycles for the single layer), sustaining a discharge capacity about 99.8 mAh g^−1^ for 700 cycles (Supplementary Fig. 26). This modest capacity gap stems from stack-level effects, including higher cumulative areal capacity and pressure or electrolyte gradients across layers, which may introduce slight electrolyte starvation and current-distribution nonuniformity in inner layers, increasing polarization and parasitic loss. Collectively, these results establish PCC as a universal, F-free binder platform capable of enabling stable operation across diverse electrode chemistries and practical pouch-cell architectures (Fig. 3e). The scalability and versatility of PCC position it as a viable pathway toward sustainable electrode manufacturing and next-generation high-performance LIBs.

### Direct recycling of PCC-based batteries

Recycling of PVDF-based batteries remains inherently challenging because of the strong C-F bonds and complex F chemistry involved. During recycling, PVDF decomposes into stable fluorinated species such as LiF and organic C-F fragments, which strongly adhere to active materials^32^. These residues not only complicate separation and purification but also persist through thermal and chemical regeneration, leading to F contamination in recycled products and additional environmental burdens^33^. Addressing this F-related challenge is therefore critical to achieving truly sustainable battery recycling.

**Fig. 4:**
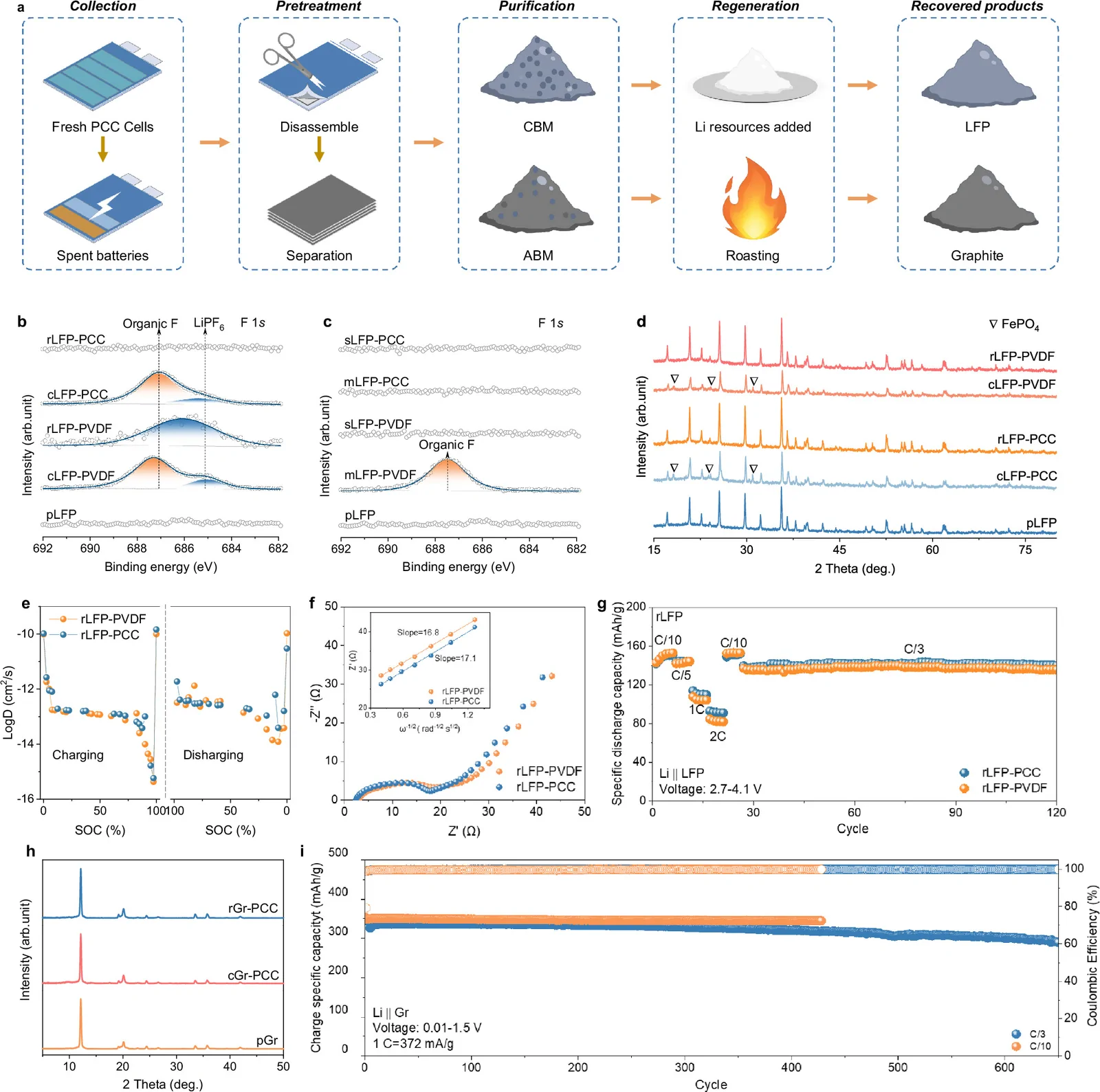
Sustainable recycling of spent LFP positive electrodes using the PCC-based binder system. **a** Schematic illustration of the recycling process. **b**, **c** F 1 *s* X-ray photoelectron spectroscopy (XPS) spectra of various LFP powders. cLFP-PVDF and cLFP-PCC denote cycled LFP powders obtained from PVDF- and PCC-based pouch cells, respectively. The electrodes were harvested in the fully discharged state after 100 cycles at C/3 (1 C = 150 mA g^−1^) at 25 ± 2 °C. rLFP-PVDF and rLFP-PCC denote the corresponding recycled LFP materials derived from the PVDF-and PCC-based electrodes, respectively. mLFP-PVDF and mLFP-PCC denote mixtures of pristine LFP with PVDF or PCC binders (98:2 by weight) used to evaluate possible fluorine residues originating from the binder after recycling. sLFP-PVDF and sLFP-PCC denote the corresponding samples after sintering at 650 °C for 4 h under argon (Ar). **d** X-ray diffraction (XRD) patterns of the corresponding LFP powders. **e** Galvanostatic intermittent titration technique (GITT) measurements of coin cells and the corresponding state-of-charge (SOC)-dependent Li^+^ diffusion coefficients of rLFP-PCC and rLFP-PVDF positive electrodes at C/10 (1 C = 170 mA g^−1^, 25 ± 2 °C). **f** Electrochemical impedance spectroscopy of coin cells employing rLFP-PCC and rLFP-PVDF electrodes. The inset shows ths linear fits of ${Z}^{{\prime} }$ versus ${{\upomega}}^{-1/2}$ in the low-frequency region for rLFP-PVDF and rLFP-PCC electrodes. **g** Rate capability and cycling performance of coin cells employing rLFP-PCC and rLFP-PVDF positive electrodes (1 C = 170 mA g^−1^, 25 ± 2 °C). **h** XRD patterns of recycled graphite form PCC-based electrodes (rGr-PCC) and cycled graphite form PCC-based electrodes (cGr-PCC), compared with pristine graphite (Gr). The cycled graphite electrodes were harvested in the fully discharged state after 100 cycles at C/3 (1 C = 372 mA g^−1^) at 25 ± 2 °C. **i** Cycling performance of coin cells employing rGr-PCC at C/10 and C/3 (1 C = 372 mA g^−1^, 25 ± 2 °C). The prefixes c, r, m, and s denote cycled, recycled, mixed, and sintered samples, respectively. Source data are provided as a Source Data file.

To overcome these limitations, we introduced this F-free, water-processable PCC binder within a direct recycling framework (Fig. 4a). Direct recycling offers an economically viable and environmentally benign pathway for recovering battery materials by preserving their bulk structure and composition^34^. Of particular interest is LFP-based batteries because of the low/negative economic return by conventional pyrometallurgical or hydrometallurgical recycling methods^35^. Following cell disassembly, cycled LFP and graphite electrodes (denoted as cLFP and cGr) were collected for regeneration. PVDF-bound electrodes delamination was performed using NMP following established pre-treatment protocols^36^. In contrast, PCC-bound electrodes were delaminated by mild soaking in deionized water, leveraging the intrinsic water solubility of PCC binder to enable efficient separation of active materials from the current collectors. X-ray photoelectron spectroscopy (XPS) of the cycled (spent) and recycled LFP powders derived from PVDF- and PCC-based batteries (rLFP-PVDF, rLFP-PCC; Fig. 4b, c and Supplementary Fig. 27) reveals pronounced F 1 *s* peaks at ~686 eV for rLFP-PVDF, corresponding to LiPF_6_ decomposition by-products from residue surface-bounded electrolyte species^37^. In contrast, rLFP-PCC shows no detectable F, demonstrating the compatibility of PCC with direct recycling and its ability to prevent accumulation of fluorinated contaminants.

Structural characterization further verifies complete recovery of the LFP phase, with no FePO_4_ impurities observed by XRD or TEM (Fig. 4d and Supplementary Fig. 28), full compositional restoration verified by elemental analysis (Supplementary Fig. 29), and a homogeneous Fe^2+^ distribution visualized by transmission X-ray microscopy (TXM) with XANES mapping (Supplementary Fig. 30). The recovered LFP from PCC system retains slurry processability comparable to the pLFP-PCC slurry system when reused in PCC (Supplementary Fig. 31). rLFP-PCC shows comparable Li^+^ diffusion, lower charge-transfer resistance (Fig. 4e, f), and higher capacity retention and rate capability compared with rLFP-PVDF (Fig. 4g). These improvements arise from the structurally intact lattice and the uniform amorphous carbon layer formed on rLFP-PCC, which together facilitate rapid Li^+^ exchange and stable interfacial charge transfer, whereas rLFP-PVDF exhibits nonuniform surface layers (Supplementary Fig. 28). Similarly, graphite directly recovered from PCC-based electrodes retains its crystalline integrity and delivers a reversible capacity of ~344.8 mAh g^−1^ at C/10 over 420 cycles, along with stable retention at a higher rate of C/3 (Fig. 4h, i). Collectively, these results demonstrate that replacing PVDF with PCC enables interfacial stability and electrochemical recovery in the direct recycling of LIBs.

### Life cycle assessments

Binder chemistry exerts a profound yet often underestimated influence on the sustainability of LIBs. To elucidate its system-level impact, we conducted a cradle-to-gate comparison between conventional PVDF-NMP and our F-free, water-processable PCC systems. Prospective life cycle assessments (LCAs), based on GREET 2024^38^, BatPac 2024^39^, and EverBatt 2023^40^, quantified environmental and economic burdens from raw-material acquisition through cell manufacturing and EoL processing. For EoL evaluation, solid-state relithiation and graphite reconstruction were benchmarked against conventional high-temperature sintering, while pyrometallurgical and hydrometallurgical pathways were excluded due to their limited suitability for LFP and graphite recovery (Supplementary Notes 2, 3)^34^. It is important to note that the LCA presented here represents a scenario-based analysis designed to enable relative comparison between PVDF-NMP and PCC-water binder systems, rather than a universal depiction of industrial practice. Several parameters, including solvent recovery efficiency, fluorinated species formation and capture, and binder removal effectiveness, can vary with regional regulations, plant design, process scale, and technology maturity. Accordingly, the results should be interpreted as indicative of comparative trends rather than absolute industrial benchmarks.

PVDF production, derived from fluorinated monomers (Supplementary Fig. 32a), carries a substantial embodied emission of ~55.8 kg carbon dioxide equivalent (CO_2_-eq)/kg and an energy demand of ~90 MJ/kg, based on literature^41^ and GREET, respectively. By contrast, PAA and CMC, sourced from renewable or bio-based feedstocks (Supplementary Fig. 32b, c), emit just 1.5-2.7 kg CO_2_-eq/kg with an energy demand of 32.2-38.7 MJ/kg^42^. The replacement of NMP ( ~ 98 MJ/kg and ~4.4 kg CO_2_-eq/kg) with water drastically reduces the solvent-related environmental burden (Supplementary Figs. 33, 34). Overall, substituting PVDF and NMP with PCC and water can significantly reduce binder-associated greenhouse gas (GHG) emissions and energy demands.

**Fig. 5:**
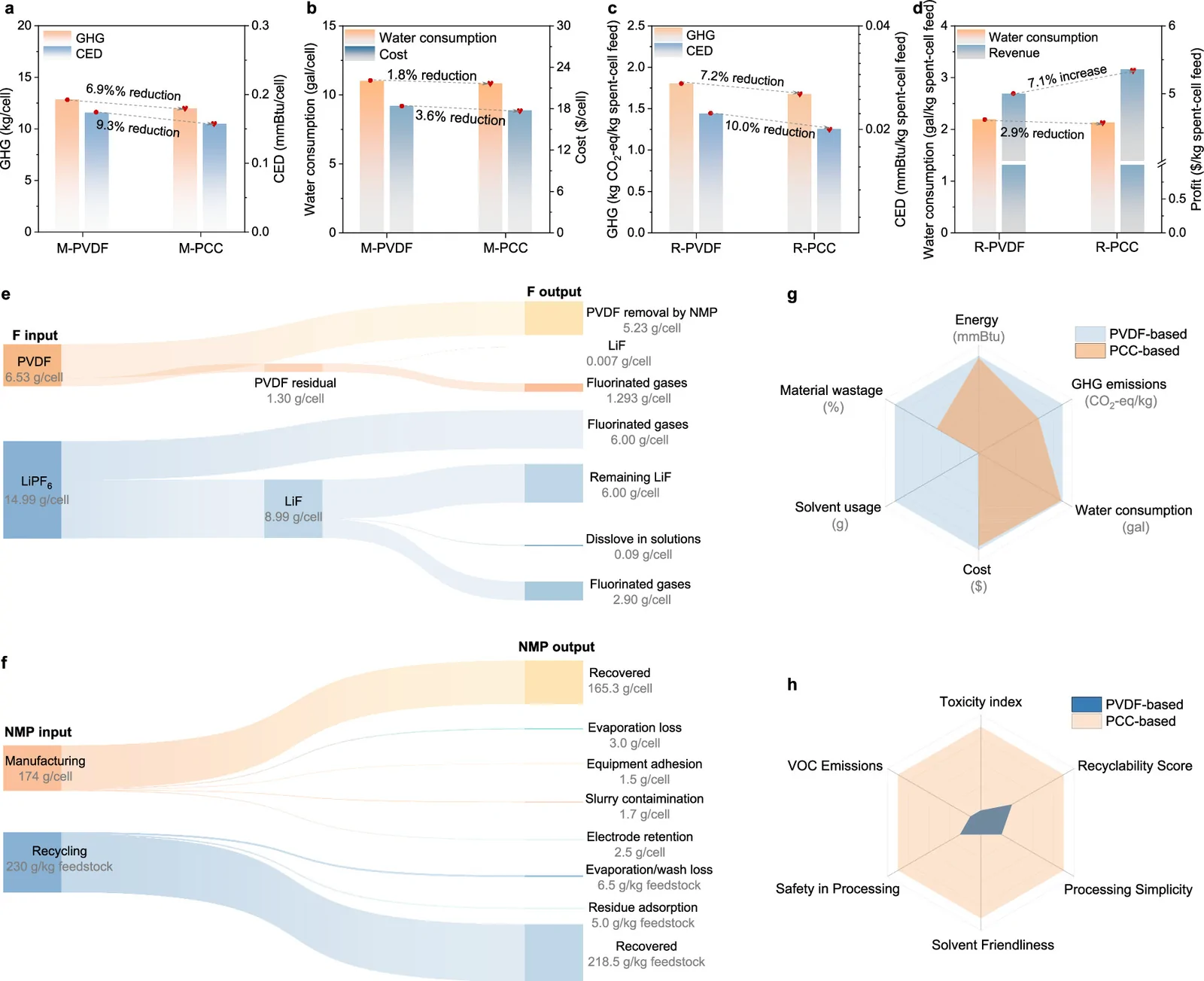
Life-cycle assessment (LCA) of PCC-based Gr | |LFP battery, with comparison of PVDF-based Gr | |LFP battery. **a** Greenhouse gas (GHG) emissions and cumulative energy demand (CED) during the manufacture of PVDF-based and PCC-based lithium-ion batteries (LIBs). **b** Water consumption and cost during the manufacture of PVDF- and PCC-based LIBs. **c** GHG emissions and CED during the recycling of PVDF- and PCC-based LIBs. **d** Water consumption and cost during the recycling of PVDF- and PCC-based LIBs. **e** Fluorine (F) input and output analysis during the manufacture and recycling process of PVDF-based LIBs. **f** N-methyl-2-pyrrolidone (NMP) input and output analysis during the manufacture and recycling process of PVDF-based LIBs. **g** Comparison of normalized environmental and process indicators for the PVDF- and PCC-based systems. **h** Qualitative techno-functional comparison based on a 10-point ordinal scale. LiPF_6_ lithium hexafluorophosphate, LiF lithium fluoride, VOC volatile organic compound, CO_2_-eq carbon dioxide equivalent, mmBtu million British thermal units. M-PVDF and M-PCC denote the manufacturing processes for PVDF- and PCC-based LIBs, respectively, whereas R-PVDF and R-PCC denote their corresponding recycling processes. Source data are provided as a Source Data file.

Integrated LCA and cost analyses (Supplementary Fig. 35, 36) show that PCC-based cell manufacture emits 11.9 kg/cell and consumes 0.157 million British thermal units (mmBtu)/cell, representing reductions of 6.9% and 9.3%, respectively, compared with PVDF-based cells (Fig. 5a). Water usage and production cost are likewise reduced to 10.8 gal and $17.7 per cell, owing to the elimination of NMP recovery and the simplicity of aqueous processing (Fig. 5b). During recycling, the PCC system further lowers energy consumption by 10.0%, water use by 2.9%, and GHG emissions by 7.2%, while increasing profit by 7.1% through streamlined operations (Fig. 5c, d).

F and solvent mass balances highlight environmental liabilities of conventional systems (Supplementary Note 3, Supplementary Fig. 37). In standard Gr | |LFP cells, F originates predominantly from PVDF ( ~ 6.5 g/cell) and LiPF_6_ (~15 g/cell). Although largely stable during fabrication, residual PVDF can decompose during recycling to generate LiF and volatile fluorinated species on the order of ~1.3 g/cell, depending on thermal conditions and off-gas management, while LiPF_6_ contributes ~9 g LiF and ~6 g of fluorinated by-products (Fig. 5e)^43^. Without appropriate off-gas control, these emissions pose environmental and safety concerns. Replacing PVDF with PCC reduces total F input by ~30% and eliminates PVDF-derived emissions (Supplementary Fig. 38), though LiPF_6_ remains a contributor to LiF deposition and volatile byproducts (e.g., POF_3_, HF)^44^. NMP usage represents an additional burden in PVDF-based processing. Approximately 174 g/cell is consumed during fabrication, with ~8.7 g lost assuming high recovery efficiency (≈95%). Recycling operations may require ~230 g NMP per kg black mass, of which ~11.5 g is lost through evaporation and washing (Fig. 5f). By contrast, PCC enables fully water-based processing, virtually eliminating volatile organic compound emissions and solvent toxicity.

Across twelve sustainability indicators, including six quantitative (energy consumption, GHG emissions, water usage, cost, solvent consumption, and material wastage) and six qualitative (toxicity index, recyclability, processing simplicity, solvent compatibility, safety, and energy demand), the PCC system shows lower calculated environment and resource impacts for the quantitative metrics and more favorable qualitative scores than the PVDF system under the defined assessment framework (Fig. 5g, h). Moreover, its ambient aqueous processing and F-free chemistry simplify EoL treatment and mitigate hazards. While absolute values are influenced by assumptions regarding solvent recovery, fluorine capture, and binder removal efficiency, reasonable variations in these parameters do not alter the qualitative conclusion that replacing PVDF-NMP with a fluorine-free, water-processable binder substantially reduces solvent-related emissions, fluorine-associated environmental burdens, and process complexity. Future work may further refine these estimates as aqueous manufacturing and direct-recycling technologies continue to mature.

## Discussion

In summary, this study establishes an F-free polymer network binder that reconciles high electrochemical performance with water-based manufacturing and direct recycling of LIBs. By integrating covalent, ionic, and hydrogen-bond interactions, the PCC system enables durable operation across diverse positive and negative electrode chemistries and pouch-cell formats while avoiding the toxic solvents and fluorinated residues. Crucially, its compatibility with direct regeneration of positive and negative electrodes, coupled with life-cycle analyses demonstrating substantial reductions in GHG emissions, energy demand and cost, illustrates how the molecular design of auxiliary components can transform the sustainability of entire technologies.

## Methods

### Binder and electrode preparation

Poly(vinylidene fluoride) (PVDF, HSV 1800, batch no. 1756520, Kureha, Japan) was used as the control binder. Polyacrylic acid (PAA, M_W_ = 3,000,000, product no. 306223, Sigma-Aldrich, USA) was pre-dispersed in deionized (DI) water at 1 wt%. The pH of the dispersion was adjusted to 6 with a 10 wt% lithium hydroxide monohydrate (LiOH·H_2_O, battery grade, Thermo Scientific Chemicals, USA) solution. Carboxymethyl cellulose (CMC, M_W_ = 250,000, product no. 419303, Thermo Scientific Chemicals, USA) was pre-dispersed at 1 wt% in DI water. Equal volumes of PAA and CMC solutions were combined (1:1), followed by the addition of citric acid (CA, ≥99.5%, Sigma-Aldrich, USA), and the mixture was stirred continuously for 8 h to obtain the PCC binder. Other binders, including PAA/CMC/Boric acid (PCB), PAA/CMC/Oxalic acid (PCO), PAA/CMC/L-ascorbic acid (PC-L-AA) were synthesized following the same procedure. Boric acid (≥99.5%, Sigma-Aldrich, USA), oxalic acid (≥99%, Sigma-Aldrich, USA), and L-ascorbic acid (≥99%, Sigma-Aldrich, USA) were used as received.

LiFePO_4_ (LFP, batch no. 06724B3, MSE Supplies, USA) served as the primary positive electrode material. Electrodes were fabricated using a slurry containing 97 wt% LFP, 1 wt% Super P conductive carbon black (≥99.5% carbon, product no. MA-EN-CO-0005, Canrd), and 2 wt% binder, with N-methyl-2-pyrrolidone (NMP, anhydrous, 99.5%, Sigma-Aldrich, USA) or DI water as solvent depending on binder type. The slurries were cast onto Al foil (15 μm Al substrate with a 1 μm carbon coating on each side, product no. MA-EN-CU-000307, Canrd) using a doctor blade. PVDF-based electrodes were dried under vacuum at 120 °C for 12 h, whereas PCC-based electrodes were dried in a convection oven at 60 °C for 4 h. Graphite negative electrodes were cast onto Cu foil (12 μm, single-bright copper foil, product no. MA-EN-CU-007201, Canrd) with a composition of 92 wt% graphite (SLC 1520p, Superior Graphite, USA), 4 wt% binder, and 4 wt% Super P, yielding an areal capacity of 3 mAh cm^−2^. LiMn_0.6_Fe_0.4_PO_4_ (LMFP, batch no. 29023C2, MSE Supplies, USA) positive electrodes were fabricated using the same protocol as LFP at an areal capacity of 1.0 mAh cm^−2^. LiNi_0.83_Co_0.11_Mn_0.06_O_2_ (NCM811, battery-grade material supplied by General Motors R&D, USA) positive electrodes were cast onto Al foil with 96 wt% NCM811, 2 wt% Super P, and 2 wt% PCC, also targeting 1.0 mAh cm^−2^. Silicon negative electrodes were cast onto Cu foil with 70 wt% Si powder (≥99.99%, D50 = 1–5 μm, batch no. HJ23112803, Fuzhou Hokin Chemical Technology Co., Ltd., China), 10 wt% Super P, and 20 wt% PCC, yielding an areal capacity of 2 mAh cm^−2^. Unless otherwise specified, electrode active materials were used as received without further purification.

### Cell assembly and electrochemical testing

Disc electrodes (12 mm diameter, single-side coated) were punched, calendared, and assembled into CR2032 stainless-steel coin cells in an Ar-filled glovebox (O_2_ < 0.1 ppm, H_2_O < 0.1 ppm). Each coin cell contained 80 μL of LP57 electrolyte (1.0 M LiPF_6_ in ethylene carbonate/ethyl methyl carbonate, 3:7 by vol., Gotion, USA, used as received). For half-cells, Li metal (99.95%, CANRD, China; 0.45 mm thick, 15.6 mm diameter) was used as the counter/reference electrode. The lithium chip was used as received without additional surface treatment before cell assembly. For full cells, graphite negative electrodes were employed at an N/P ratio of ~1.1. The separator was a commercially available Celgard 2320 separator (Celgard LLC, USA), consisting of a trilayer polypropylene/polyethylene/polypropylene (PP/PE/PP) membrane with a nominal thickness of 20 μm, a diameter of 19 mm (for CR2032 coin cells), a porosity of 39%, and an average pore size of 0.043 μm. Galvanostatic cycling was performed on a Neware battery tester. Li | |LFP half-cells were cycled between 2.7–4.1 V, and Gr | |LFP full cells between 2.4–4.0 V, with three formation cycles at C/10 followed by cycling at C/3 (1 C = 170 mA g^−1^ for half-cells, 1 C = 150 mA g^−1^ for pouch full-cells). Pouch cells were assembled using single-side-coated electrodes measuring 3.3 cm × 3.3 cm. Each pouch cell was filled with 500 μL of electrolyte and subsequently vacuum sealed. The sealed cells were allowed to rest for 30 h before electrochemical testing to allow electrolyte wetting. No degassing step was performed. During cycling, the pouch cells were held in a mechanical fixture to maintain cell alignment. The applied stack pressure was not actively controlled or measured. All cycling tests were performed at 25 ± 2 °C unless otherwise specified. Li | |NCM half-cells were cycled from 3.0–4.3 V, Li | |LMFP half-cells from 2.5–4.5 V, and Li | |Gr and Li | |Si half-cells from 0.01-1.5 V (25 ± 2 °C). Galvanostatic intermittent titration technique (GITT) was conducted in the second cycle, applying 10 min current pulses at 0.1 C within 2.7–4.1 V, followed by 180 min relaxation (25 ± 2 °C). Diffusion coefficients were calculated from the transient and steady-state voltage responses. The collected data were analyzed to determine the diffusion coefficient of the material. High-temperature cycling tests were conducted using LMFP positive electrodes to evaluate binder stability under elevated voltage and temperature. Li | |LMFP half-cells were cycled in the voltage window of 2.5-4.5 V versus Li/Li⁺ at a controlled temperature of 42 °C. After formation of first cycle at C/10 (1 C = 150 mA g^−1^, 25 ± 2 °C), galvanostatic cycling was performed at a C/3 rate (1 C = 150 mA g^−1^, 42 °C).

In-situ electrochemical impedance spectroscopy (EIS) was performed on VSP-300 (BioLogic) with a 20 mV perturbation over 7 MHz-0.05 Hz (six points per decade). To ensure that differences in impedance evolution arise solely from binder chemistry, the electrolyte composition, cell configuration, and formation protocols were kept identical across all binder systems. Specifically, both LFP-PCC and LFP-PVDF cells employed the same LP57 electrolyte, separator, and electrode loading, and were subjected to identical formation and testing procedures. For impedance tracking of rLFP-PCC and rLFP-PVDF systems, cells were charged at 0.1 C for 1 h, rested for 0.5 h, and then subjected to EIS measurements. This sequence was repeated until reaching the cutoff voltage. Data were analyzed using a MATLAB-based DRT toolbox^45^. The anodic and cathodic stability windows of the PVDF and PCC were evaluated by cyclic voltammetry by scanning from the open-circuit voltage to 6 V and 0 V, respectively, at 0.1 mV S^−1^ using a VSP-300 at 25 °C. It is important to note that DRT peak assignment in porous composite electrodes is inherently non-unique, as individual relaxation features may reflect overlapping contributions from interfacial charge transfer, ionic transport through binder domains, and electrode porosity effects. Accordingly, DRT is used here as a comparative diagnostic tool rather than as a definitive assignment of discrete physicochemical processes.

The electronic conductivities of the LFP/binder were evaluated using DC polarization techniques via a VSP-300. All preparations were carried out under an Ar-filled atmosphere (VIGOR, O_2_ < 1 ppm, H_2_O  < 1 ppm). A symmetric cell with a configuration of Ti|LFP+binder|Ti was used for electronic conductivity measurement. The positive electrode powders (LFP-PCC and LFP-PVDF, 98:2 wt%, pristine LFP powder was tested as controls) were cold-pressed into cylindrical pellets (10 mm diameter) using a hydraulic press (Model 4350 Carver Bench Top Laboratory Pellet Press) under a pressure of 360 MPa for 3 min, and two Ti rods were employed as non-reactive ion-blocking electrodes. Applying 100–500 mV for the symmetric cell and recording steady-state current. During testing, an external pressure of 50 MPa was maintained. The ionic and electronic conductivities were calculated using the following formula: $\sigma=\frac{L}{R\times A}$ where L, R, and A represent the thickness, resistance, and contact area of the composite positive electrode pellet, respectively. In this study, A is fixed as 0.785 cm^2^.

Electrolyte uptake of PCC binder films with different CA contents was evaluated using a gravimetric swelling method. Free-standing binder films were prepared by casting the corresponding aqueous binder solutions onto polyethylene terephthalate (PET) substrates, followed by drying at 80 °C until complete water removal. The films were further dried under vacuum at 60 °C overnight to remove residual moisture and equilibrated to 25 °C prior to testing. Film samples were cut into coupons of similar dimensions, and their dry mass (m_dry) was measured using an analytical balance. Electrolyte uptake tests were conducted by immersing the film coupons in LP57 electrolyte at 25 °C. After 1 h soaking, the samples were removed, gently blotted to eliminate surface-adhered electrolyte, and immediately weighed to obtain the wet mass (m_wet). Electrolyte uptake was calculated as: ${\mbox{Electrolyte uptake}}$ $(\%)=\frac{{{\mbox{m}}}\_{{\rm{wet}}}-{{\mbox{m}}}\_{{\rm{dry}}}}{{{\mbox{m}}}\_{{\rm{dry}}}{{\mbox{x}}}}100$. Film integrity after electrolyte exposure was confirmed by visual inspection to ensure that mass changes arose from electrolyte absorption rather than dissolution.

Leakage-current measurements were performed to evaluate the electrochemical stability of PCC under 4.1 V cut-off conditions. Li | |LFP coin cells were charged galvanostatically to the 4.1 V and then held at 4.1 V for 100 h duration while the current response was continuously recorded using a VSP-300 potentiostat (BioLogic). The resulting steady-state current was taken as the leakage current, reflecting parasitic reactions and interfacial instability under prolonged polarization.

### Direct recycling of LFP and graphite

Gr | |LFP pouch cells containing PVDF- or PCC-based electrodes were assembled in an Ar-filled glovebox and filled with 500 μL of electrolyte per cell. The cells were vacuum sealed (MTI vacuum sealer) and activated between 2.4–4.0 V at C/10 for three cycles. After 100 total cycles, cells were disassembled, and positive and negative electrode sheets were collected for recycling. For LFP recycling, the recovered cathode sheets were stirred in 20 mL of NMP (PVDF system) for 2 h or 20 mL of DI water (PCC system) for 5 min to delaminate the active material from the Al current collector. The resulting black mass (BM) was collected by centrifugation and washed 3 times with 5 mL of ethanol per wash for the PVDF system or 5 mL of DI water per wash for the PCC system. The washed black mass was vacuum-dried at 80 °C for 12 h. Li_2_CO_3_ was added to adjust Li:Fe to 1.02. The mixture was sintered in Ar at 540 °C for 3 h followed by 650 °C for 10 h. The regenerated powders were re-fabricated into coin cells for electrochemical evaluation. For graphite recycling, the recovered negative electrodes were stirred in 20 mL of DI water for 5 min to delaminate the graphite from the current collector. The recovered graphite powders were collected by centrifugation, washed 3 times with 5 mL of DI water per wash, and vacuum-dried at 80 °C for 12 h. The dried powder was annealed under Ar at 1000 °C for 4 h and subsequently evaluated in Li | |Gr half-cells. No quantitative mass-based recovery yield was determined for the experimental recycling process.

### Materials characterization

Elemental compositions were determined by an inductively coupled plasma quadrupole mass spectrometer (ICP-MS, Thermo Scientific, iCAP RQ model). Shear-rate-dependent viscosity measurements were conducted over a range of 0.1-15 s⁻¹, using a Discovery Hybrid Rheometer HR-3. The upper shear-rate limit was constrained by instrument torque sensitivity under the selected measurement geometry and high-solids slurry formulation. This range captures shear conditions relevant to slurry mixing, structural relaxation, and laboratory-scale doctor-blade coating. Thermogravimetric analysis (TGA) and differential scanning calorimetry (DSC) were performed by using TA Instruments thermal analyzers (Discovery SDT 650). High-resolution transmission electron microscopy (HRTEM) was performed on a Talos 200X TEM (200 kV, ThermoFisher) with the CETA Camera. The detailed structure information was measured by DigitalMicrograph (DM). Crystal structures were examined by X-ray powder diffraction (XRD, Rigaku Miniflex, Cu Kα radiation, *λ* = 1.5406 Å). Surface chemistry was probed by X-ray photoelectron spectroscopy (XPS, PHI 5000 VersaProbe II, Al K*α* radiation at 1486.6 eV,) calibrated to C 1 s peak at 284.8 eV. XPS depth profiling was carried out using an Ar^+^ gas cluster ion beam operated at 10 keV with an ${{\mbox{Ar}}}_{1000}^{+}$ cluster size. The sample surface was sequentially etched for 60 s per cycle, with a total of three sputtering cycles performed to probe the depth-dependent chemical composition. Survey and high-resolution spectra (C 1 *s*, O 1 *s*, F 1 *s*, P 2*p*, and Li 1 *s*) were collected after each sputtering step. Sputtering time was used as a relative depth coordinate due to uncertainties in sputter yield in porous composite electrodes. For ex situ TEM and XPS characterization, the cells were disassembled in an Ar-filled glovebox (O_2_ < 0.1 ppm and H_2_O < 0.1 ppm). The harvested electrodes were rinsed three times with dimethyl carbonate to remove residual electrolyte and dried inside the glovebox. The electrodes were transferred from the glovebox to the characterization facilities in sealed containers to minimize air exposure prior to analysis. Fourier transform infrared (FTIR) spectra were collected using a Thermo Scientific Nicolet 6700 FTIR spectrometer equipped with a Smart‑iTR^TM^ diamond attenuated total reflectance (ATR) accessory. Spectra were acquired over the range of 4000–400 cm^−1^ at a resolution of 4 cm ^−1^, with 32 scans averaged for each spectrum. The binder samples were dried at 80 °C for 12 h under ambient conditions before measurement and placed directly on the diamond ATR crystal without further preparation. Dynamic light scattering (DLS) measurements were performed using a DynaPro Plate Reader III (Wyatt Instruments) at 25 °C. Water-processable binder samples were prepared in DI water at 1 wt%, whereas PVDF was dissolved in NMP at 0.2 wt%. Data were acquired and analyzed using the instrument-associated DYNAMICS software with the measurement settings used at the time of data collection. Transmission X-ray Microscopy (TXM) was performed at National Synchrotron Light Source II of Brookhaven National Laboratory on Beamline FXI. The three-dimensional X-ray absorption near-edge structure (XANES) map was acquired with a spatial resolution of 30 nm. Data post-processing was completed with TXM-Sandbox and Dragonfly 3D World. Fe K-edge XANES and extended X-ray absorption fine structure (EXAFS) measurements were performed at 7-BM, Quick X-ray Absorption and Scattering at NSLS II.

### Environmental analysis

Life-cycle assessment (LCA) and cost analyses were performed using the Greenhouse gases, Regulated Emissions, and Energy use in Transportation (GREET) 2024^38^, Battery Performance and Cost (BatPac) 2024^39^, and EverBatt 2023^40^ developed at Argonne National Laboratory to compare PVDF/NMP- and PCC/water-based Gr | |LFP battery systems. The functional unit for the manufacturing analysis was one 0.25-kWh pouch cell, whereas recycling results were normalized to 1 kg of spent-cell feed. The analysis represents an industrial-scale U.S. context based on the material, energy, process, and cost inventories implemented in these model versions; no temporal adjustment or cost escalation was applied. The manufacturing boundary included electrode-material production, electrode fabrication, cell assembly, formation, and integration, whereas the recycling boundary included preprocessing, electrode separation, binder removal, active-material recovery, LFP relithiation, graphite regeneration, utilities, and waste treatment. Both manufacturing scenarios used 15 μm Al foil, 10 μm Cu foil, and an N/P ratio of 1.1. The PVDF-based positive electrode contained 96 wt% LFP, 2 wt% PVDF, and 2 wt% conductive carbon and was processed using NMP, whereas the PCC-based positive electrode contained 97 wt% LFP, 2 wt% PCC, and 1 wt% conductive carbon and was processed using water. Detailed inventories and unit prices are provided in Supplementary Tables 6–13.

During preprocessing, 95% of the LFP and graphite was assumed to enter the recovered black-mass stream, whereas 90% of the Al and Cu current collectors was recovered. Subsequent direct recycling recovered 90% of the LFP and graphite in the black mass, giving an overall active-material recovery yield of 85.5%. The preprocessing model generated 0.665 and 0.655 kg of black mass per kilogram of PVDF- and PCC-based spent-cell feed, respectively. Accordingly, CM-recovery inputs, environmental burdens, costs, and revenues calculated per kilogram of black mass were multiplied by the corresponding black-mass yield before being combined with the preprocessing results. A baseline NMP recovery efficiency of 95% was assumed. Reported metrics included greenhouse-gas (GHG) emissions, cumulative energy demand (CED), water consumption, and listed process-input cost. The latter included the material, energy, water, and waste-treatment inputs specified in the inventory but excluded diesel, capital, labor, maintenance, and overhead costs where data were unavailable. No numerical cutoff threshold, allocation among recovered products, or avoided-production credit was applied. Fluorine inputs and potential output pathways were estimated by mass balance, but off-gas and wastewater fluorine-capture efficiencies were not modeled. The comparison was deterministic, and no formal sensitivity or quantitative uncertainty analysis was performed.

For Fig. 5g, the calculated energy consumption, GHG emissions, water consumption, cost, solvent use, and material waste were normalized to the corresponding PVDF-based values, which were set to 1.0. This normalization was used only for visualization. The techno-functional criteria in Fig. 5h were evaluated separately using a 10-point ordinal scale, with scores of 1–2, 3–4, 5–6, 7–8, and 9–10 representing very unfavorable, unfavorable, moderate, favorable, and very favorable performance, respectively. Scores were assigned using predefined criterion-specific experimental, modeling, literature, or safety evidence. Because these scores represent qualitative judgments rather than measured quantities, no statistical uncertainty was assigned. Their inherent subjectivity is acknowledged, and the scores were used only for qualitative comparison and visualization. Detailed criteria, scores, and supporting evidence are provided in the Source Data file.

Figures 1, 2k, l, 4a, and Supplementary Figs. 16 and 37 were created using Microsoft PowerPoint, while Fig. 5g, h were generated using OriginPro.

## Supplementary information

Supplementary Information

Transparent Peer Review file

## Source data

Source Data

## Data Availability

The data supporting the findings of this study are available within the Article, its Supplementary Information and the Source Data file. Source data underlying Fig. 2–5, Supplementary Fig. 2-5, Supplementary Fig. 8-15, Supplementary Fig. 19–24, and Supplementary Fig. 26, 27, 29, 31, 34, and 38 are provided with this paper. Additional details on datasets and protocols that support the findings of this study will be made available by the corresponding author upon request. Source data are provided with this paper.
