# Supplementary material for: Fluorine-free polymer binder enables circular manufacturing and recycling of lithium-ion batteries
Source: Nat Commun. 2026 Oct 9;17:11473. doi: 10.1038/s41467-026-78215-3 (PMC13740254; doi:10.1038/s41467-026-78215-3)
Supplement: Supplementary file 1 [file 41467_2026_78215_MOESM1_ESM.pdf]

Supplementary Information

Fluorine-free polymer binder enables circular manufacturing and recycling of lithium-ion batteries

Jiao Lin[1], Anthony Mu[1], Wei Tang[1], Feng Li[1], Yuwei Zhu[2], Mingqian Li[1], Ram Hemanth Yeerella[3], Christopher Potts[1], Shengqiang Cai[3,4], Linqin Mu[2], John Muldoon[5*], Zheng Chen[1,4,6*]

[1]Aiiso Yufeng Li Family Department of Chemical and Nano Engineering, University of California San Diego, La Jolla, California 92093, USA

[2]Materials Science and Engineering, School for Engineering of Matter, Transport and Energy, Arizona State University, Tempe, AZ 85212, USA

[3]Department of Mechanical and Aerospace Engineering, University of California San Diego, La Jolla, California 92093, USA

[4]Program in Materials Science and Engineering, University of California San Diego, 9500 Gilman Dr, La Jolla, CA 92093, USA

[5]Toyota Research Institute of North America, 1555 Woodridge Avenue, Ann Arbor, Michigan 8105, USA

[6]Sustainable Power and Energy Center, University of California San Diego, La Jolla, California 92093, USA

Corresponding to: zhc199@ucsd.edu (Z.C.), john.muldoon@toyota.com (J. M.)

Table of contents

Supplementary Figures

Supplementary Tables

Supplementary Notes

Supplementary References

Supplementary Figures

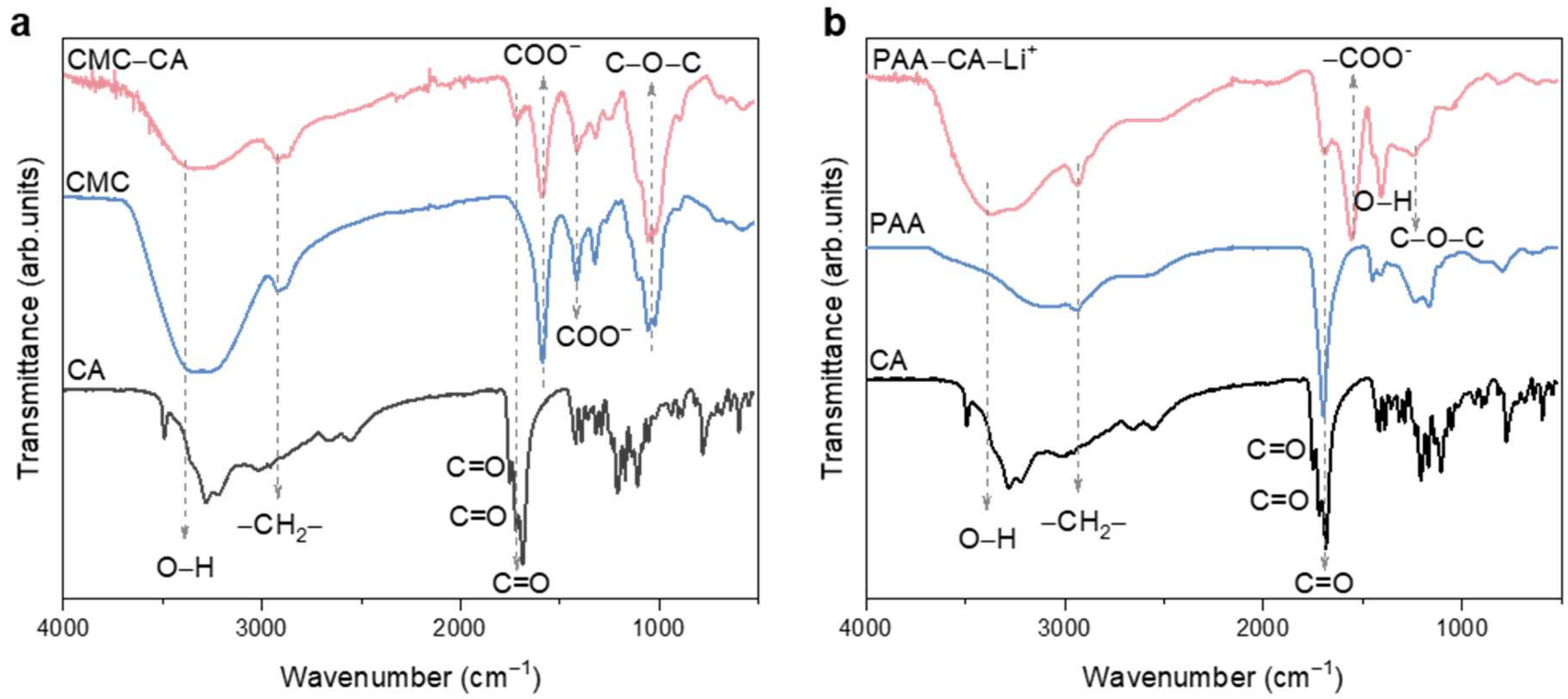

Supplementary Figure 1: FTIR analysis of CA-induced interactions in PAA and CMC. a, FTIR spectra of CA, CMC and CMC-CA system. **b**, FTIR spectra of CA, PAA and PAA-CA mixture. For the PAA-CA system, LiOH was added to adjust the pH to ~6, whereas the CMC-CA sample required no adjustment since its pH was already near 6. In both cases, the CA content was fixed at 5 wt%.

In the FTIR spectrum of pristine PAA, the peaks at ~2930 cm^{-1} is assigned to CH_2 stretching, while the strong absorption near ~1700 cm^{-1} corresponds to the C=O stretching vibration of carboxylic acid groups. The bands at ~1446 and 1417 cm^{-1} are associated with CH_2 bending and carboxyl-related vibrations. For CMC, the characteristic band at ~1595 cm^{-1} is assigned to the asymmetric stretching of COO^- groups, while the band at ~1410 cm^{-1} corresponds to the symmetric stretching of COO^-. The absorptions at ~1270 and ~1045 cm^{-1} are attributed to C-O- and C-O stretching vibrations in the polysaccharide backbone[1]. In addition, CA exhibits strong carbonyl-related bands at ~1720 and 1673 cm^{-1}, which are associated with carboxylic acid groups in different hydrogen-bonding environments[2].

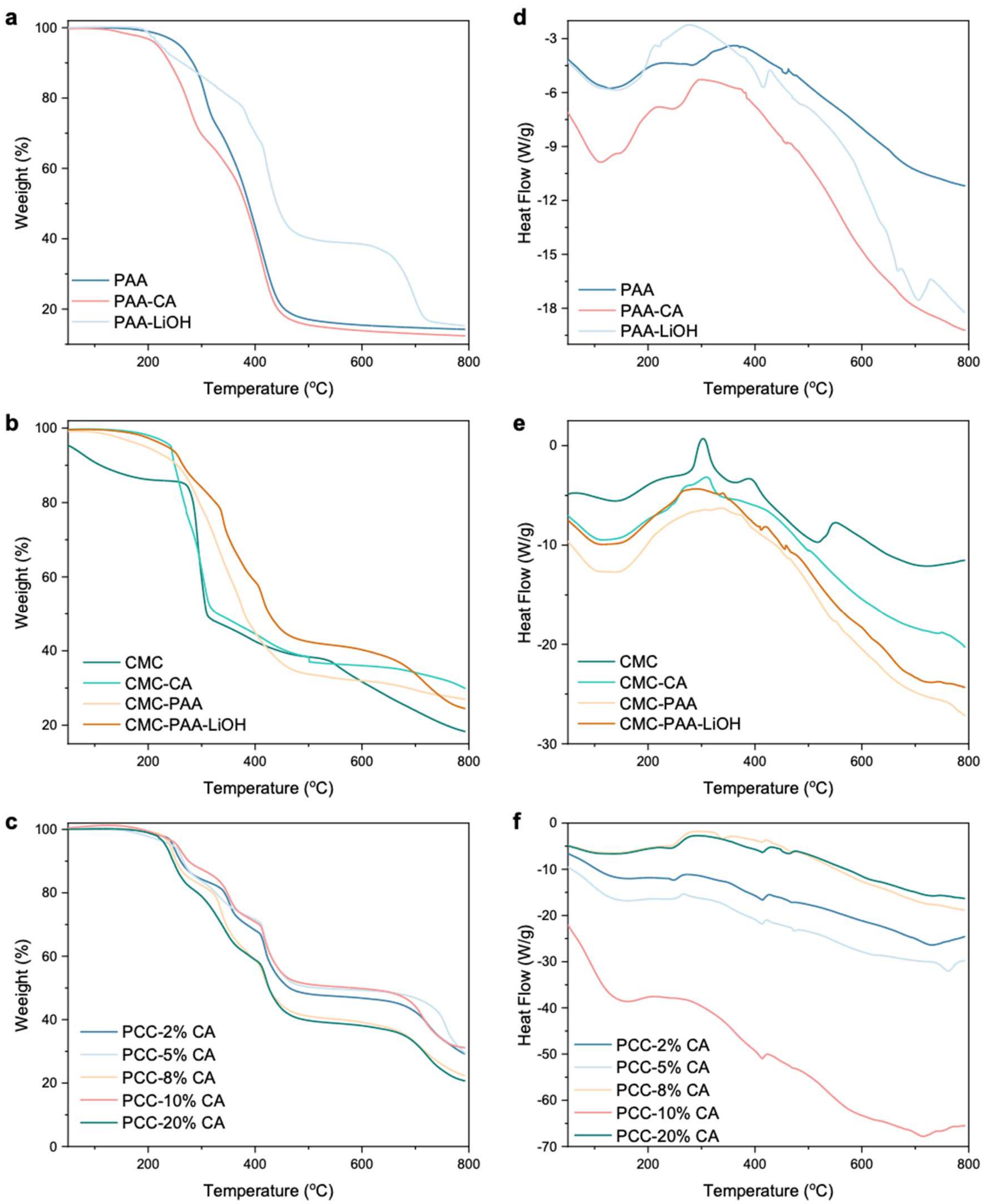

Supplementary Figure 2: Thermal behaviour of binder systems. a, b, c, Thermogravimetric analysis of the binder formulations indicated in the legends. **d, e, f,** Corresponding heat-flow traces for the same samples. All measurements were conducted under identical conditions; exotherm/endotherm directions and heating rates follow the instrument convention. DSC results

are used qualitatively to assess relative changes in the thermal response and apparent network evolution of the PCC binders, rather than as a direct or quantitative measure of crosslink density.

The thermal properties of PAA, PAA-CA, and PAA-LiOH binders were evaluated using differential scanning calorimetry (DSC) and thermogravimetric analysis (TGA) to elucidate how chemical modification influences the thermal response of the binder system (**Supplementary Fig. 2a, d**). Pure PAA remains thermally stable below ~250 °C, followed by an initial mass loss stage from ~250-320 °C and a major decomposition step between ~320 and 430 °C, consistent with progressive polymer backbone degradation. In contrast, PAA-CA displays a pronounced heat-flow feature in the range of ~150-200 °C, which is likely associated with thermally activated esterification and crosslinking reactions between CA and PAA chains. Correspondingly, TGA reveals an earlier onset of mass loss (~220-250 °C), followed by the main decomposition process up to ~430 °C, suggesting that the presence of CA modifies the thermal decomposition pathway. PAA-LiOH shows a distinct two-step decomposition behavior. The first mass loss stage occurs from ~250 to 450 °C, followed by a second decomposition step at ~620 to 720 °C, likely associated with the breakdown of lithium carboxylate species and residual inorganic lithium-containing phases. Compared with PAA and PAA-CA, the broader decomposition window and delayed high-temperature mass-loss step indicate improved thermal persistence after LiOH neutralization. Overall, these results suggest that CA promotes thermally induced network formation, whereas LiOH modification stabilizes the binder through formation of lithium carboxylate structures with distinct multi-step decomposition behavior.

TGA-DSC was performed on CMC and its chemically modified variants to evaluate the effects of polymer blending, crosslinking, and lithium coordination on thermal stability and decomposition pathways (**Supplementary Fig. 2b, e**). Pristine CMC exhibits an initial minor weight loss below ~150 °C, accompanied by a broad DSC thermal event, which is attributed to the removal of physically adsorbed and bound water associated with the hydrophilic polysaccharide backbone. A major mass loss step is observed between ~250-350 °C, where the weight decreases sharply from ~90% to ~50%, corresponding to the primary decomposition of the CMC backbone through glycosidic bond cleavage, dehydration, and decarboxylation reactions. This process is consistent with the pronounced DSC peak centered at ~300 °C. At higher temperatures (~350-750 °C), a more gradual mass loss is observed, which can be assigned to further decomposition and

carbonization of residual polymer fragments. CMC-CA shows a similar overall decomposition profile to CMC but with slightly earlier onset of mass loss and reduced residual mass evolution at elevated temperature, suggesting that the introduction of citric acid modifies intermolecular interactions and may induce partial esterification between hydroxyl and carboxyl groups during heating. Correspondingly, the DSC response shows a broadened and less intense thermal transition around ~280-320 °C, indicating altered decomposition kinetics relative to pristine CMC. For the CMC-PAA system, the major decomposition step shifts to a broader temperature range (~280-450 °C), with TGA showing a more gradual weight-loss process compared with pristine CMC. This behavior is consistent with the formation of an interpenetrated hydrogen-bonded polymer network between CMC hydroxyl groups and PAA carboxyl groups, which increases structural connectivity and broadens the decomposition pathway[3]. The corresponding DSC trace exhibits an extended thermal event spanning ~100-350 °C, supporting stronger interpolymer interactions and a more distributed bond dissociation process[4]. Upon LiOH incorporation, the CMC-PAA-LiOH sample exhibits the broadest decomposition window (~280-500 °C) and the highest mass retention in the intermediate temperature region (~400-650 °C), indicating enhanced thermal robustness of the polymer matrix. This behavior is attributed to the formation of lithium carboxylate coordination sites within the CMC-PAA network, which introduces ionic crosslinking and stabilizes the polymer framework during thermal decomposition. The broadened DSC response and suppressed low-temperature thermal event further support the presence of a more thermally distributed decomposition process in the lithium-coordinated binder system.

The thermal behaviors of PCC binders containing different citric acid (CA) contents (2-20 wt.%) were evaluated by TGA and DSC to examine the influence of crosslinker concentration on thermal decomposition and network evolution (**Supplementary Fig. 2c, f**). All PCC samples exhibit similar multistep decomposition behavior. A minor low-temperature thermal event below ~150 °C is assigned to the removal of residual moisture. This is followed by the first major mass-loss stage at ~220-320 °C, corresponding to the onset of thermally activated condensation reactions and initial decomposition of the polymer network, and a second major decomposition stage at ~320-450 °C, associated with further breakdown of the crosslinked PAA-CMC framework and cleavage of CA-mediated ester linkages. A third gradual mass-loss stage above ~650 °C is attributed to the decomposition and carbonization of residual fragments. The DSC profiles show that the heat-flow

response evolves systematically with CA content, indicating that the crosslinker concentration strongly affects the thermal reaction pathway of the PCC network. Among all samples, the 20% CA binder shows the lowest residual mass at high temperature, indicating that excessive CA does not improve thermal robustness and may instead introduce a larger fraction of thermally labile linkages.

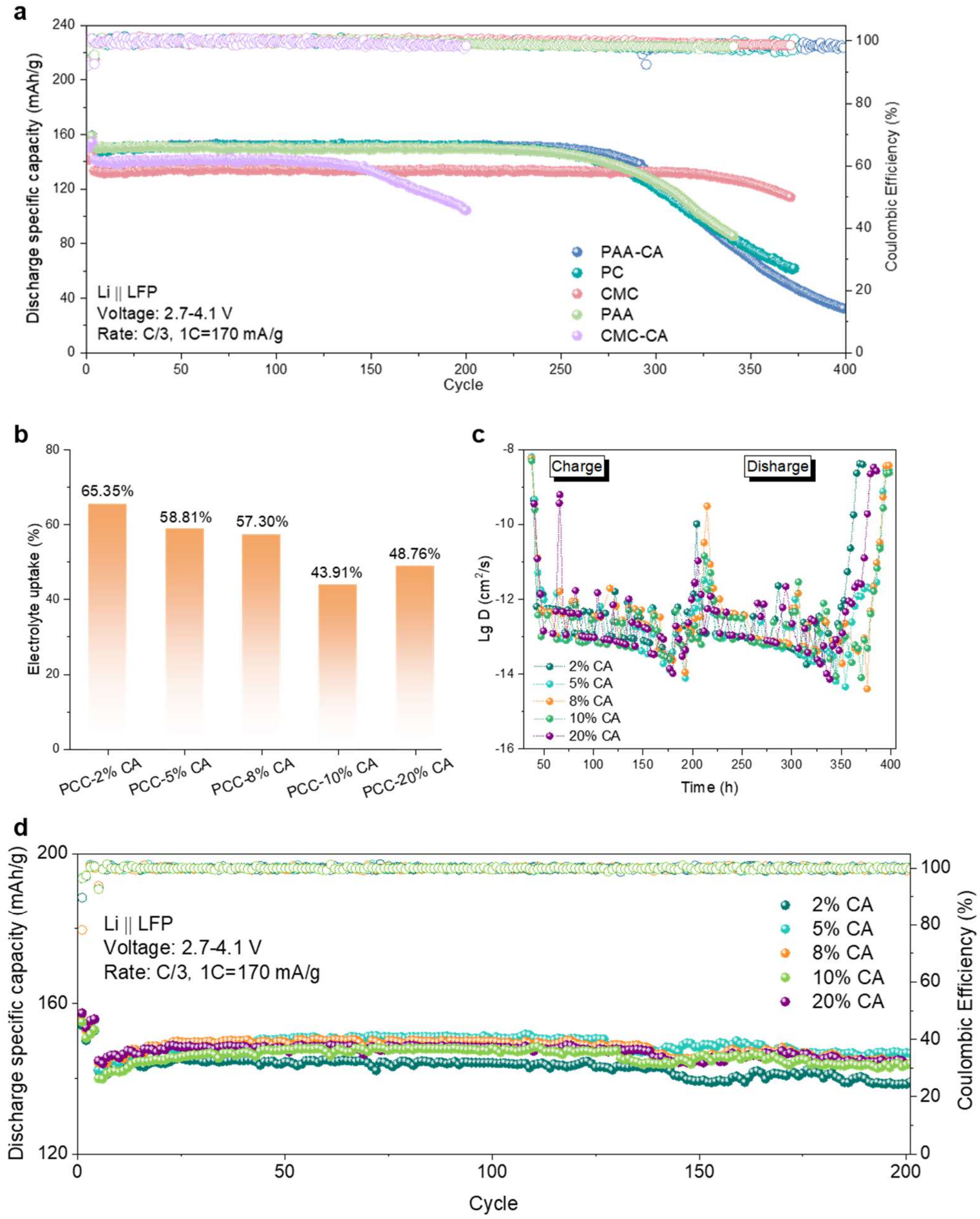

Supplementary Figure 3: Effect of citric acid (CA) content on PCC binder properties and electrochemical performance. a, Electrochemical performance comparison of PAA/CMC binders with and without esterification crosslinking. Cycling stability of electrodes prepared with PAA-CA, PAA-CMC-LiOH (PC), CMC, CMC-CA, and PAA-LiOH binders (1 C = 170 mA g^{-1}, 25 ± 2 °C). The comparison isolates the effect of esterification-enabled covalent crosslinking from physical blending and hydrogen-bond interactions, highlighting its role in stabilizing electrochemical performance during prolonged cycling. **b,** Electrolyte uptake of PCC binder films

with varying CA contents, illustrating the influence of crosslinking density on solvent absorption behavior. **c**, Galvanostatic intermittent titration technique (GITT) profiles during charge and discharge of LFP positive electrodes prepared with different CA loadings, comparing Li^+ diffusion behavior. GITT measurements were conducted in the second cycle following first-cycle activation at C/20 (1 C = 170 mA g^{-1}) at 25 ± 2 °C. **d**, Long-term cycling performance of LFP electrodes employing PCC binders with varying CA contents (1 C = 170 mA g^{-1}, 25 ± 2 °C), highlighting the impact of crosslinking level on capacity retention and electrochemical durability.

As shown in **Supplementary Figure 3a**, the physically mixed binder exhibits inferior cycling stability and accelerated capacity decay compared with PCC, despite having identical polymer composition and additive content. These results demonstrate that the observed performance improvements cannot be attributed solely to hydrogen bonding or physical blending of PAA, CMC, and CA. Instead, the esterification-induced covalent network formation plays a critical role in stabilizing electrode structure and interfacial integrity during prolonged cycling.

To elucidate the role of CA crosslinking density in regulating binder functionality, we systematically evaluated electrolyte uptake, Li^+ transport behavior (via GITT), and long-term cycling stability for PCC binders with varying CA contents. As shown in **Supplementary Fig. 3b**, electrolyte uptake decreases progressively with increasing CA addition. At low CA content (2% CA), the polymer network remains loosely crosslinked, resulting in higher free volume and greater solvent absorption. While enhanced swelling may facilitate ionic transport, excessive uptake can compromise mechanical integrity[5]. In contrast, higher CA loading (5% and 8%) increases crosslink density, restricts polymer chain mobility, and reduces solvent uptake, indicating formation of a more compact network. However, overly dense crosslinking (10% and 20%) may impede electrolyte penetration and Li^+ accessibility within the electrode matrix. GITT analysis (**Supplementary Fig. 3c**) shows that electrodes with low-to-intermediate CA contents (2-8 wt%) exhibit slightly higher apparent Li^+ diffusion coefficients compared with highly crosslinked counterparts, suggesting that excessive crosslink density imposes additional transport resistance.

The cycling performance (**Supplementary Fig. 3d**) further supports the existence of an optimal CA window. Moderate CA addition (5% and 8%) delivers higher discharge capacities, whereas

both insufficient (2 wt%) and excessive (≥10 wt%) crosslinking lead to reduced capacity. The former likely suffers from inadequate mechanical cohesion and interfacial stability, while the latter is associated with restricted ionic transport and increased internal resistance. Collectively, these results establish a clear structure-property-performance correlation within the PCC binder system. CA crosslinking density regulates the trade-off between mechanical stability and ionic transport, with an intermediate crosslinking level optimizing interfacial integrity, Li^+ diffusion, and overall electrochemical performance.

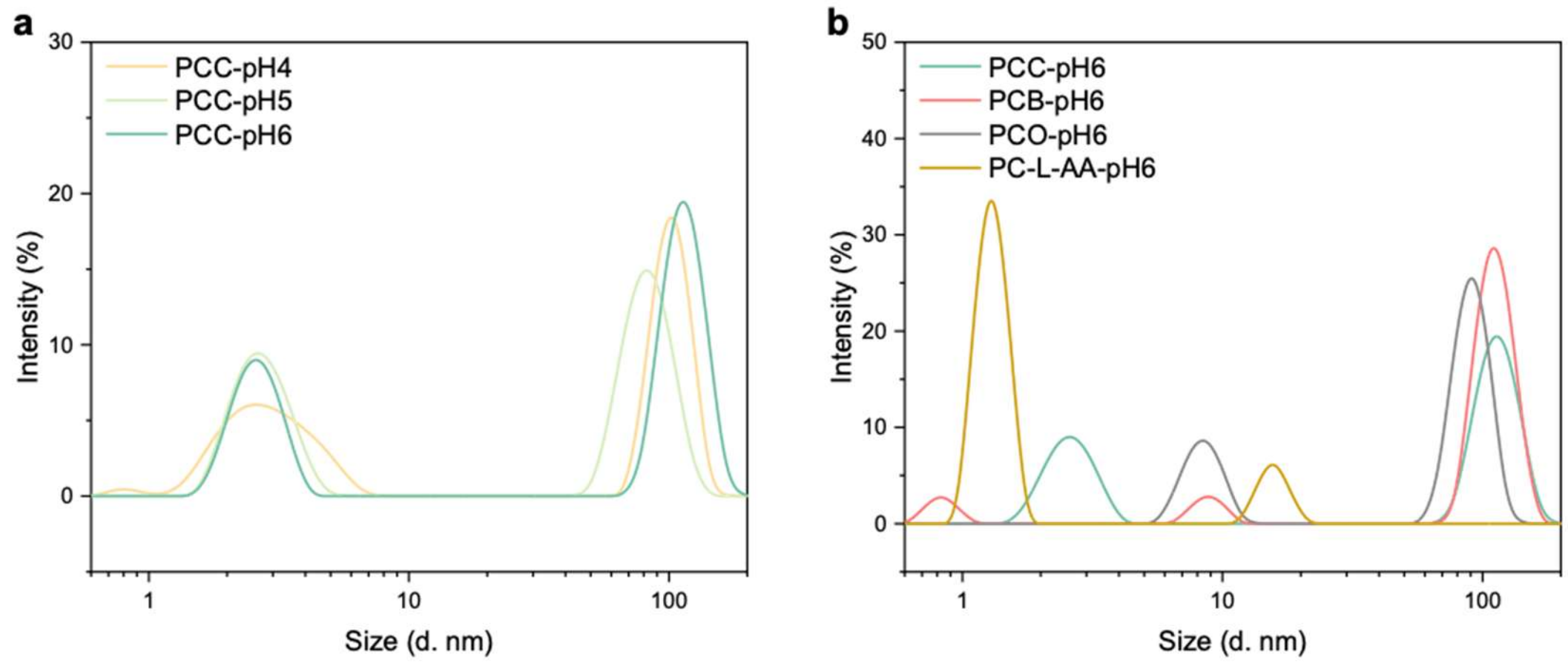

Supplementary Figure 4: Colloidal behavior of aqueous polymer binders. **a**, Dynamic light scattering (DLS) analysis of PCC (PAA + CMC + CA + LiOH) at different pH values shows nearly overlapping distributions, demonstrating robust colloidal stability across a wide pH range. **b**, Influence of different small-molecule additives on PC (PAA + CMC) dispersions.

The hydrodynamic radii remain centered in a narrow range, with only minor shifts in peak position and intensity. This suggests that the CA-mediated crosslinking and LiOH neutralization endow PCC with robust colloidal stability that is largely insensitive to pH fluctuations within the tested range (**Supplementary Fig. 4a**). Such stability is advantageous for slurry preparation, as it ensures reproducible dispersion behavior without requiring stringent pH control. Dynamic light scattering (DLS) was used to probe how different small-molecule additives influence the colloidal behavior of the PC binder (PAA + CMC) (**Supplementary Fig. 4b**). The addition of citric acid (PCC) produces a relatively narrow and uniform distribution, consistent with multidentate coordination that stabilizes interpolymer networks. In contrast, boric acid (PCB) results in broad, multimodal peaks, indicating heterogeneous interactions and poor colloidal stability. Oxalic acid (PCO) yields a mixed profile with both small- and intermediate-size aggregates, reflecting weaker diacid bridging that only partially stabilizes the dispersion. The incorporation of L-ascorbic acid (PC-L-AA) leads to a complex spectrum with populations at both small and large sizes, suggesting competing effects of hydrogen bonding, chain association, and partial aggregation. Together, these results demonstrate that the nature of the additive plays an important role in tuning the dispersion state of PC, with CA providing the most uniform and stable aqueous dispersions among the additives examined.

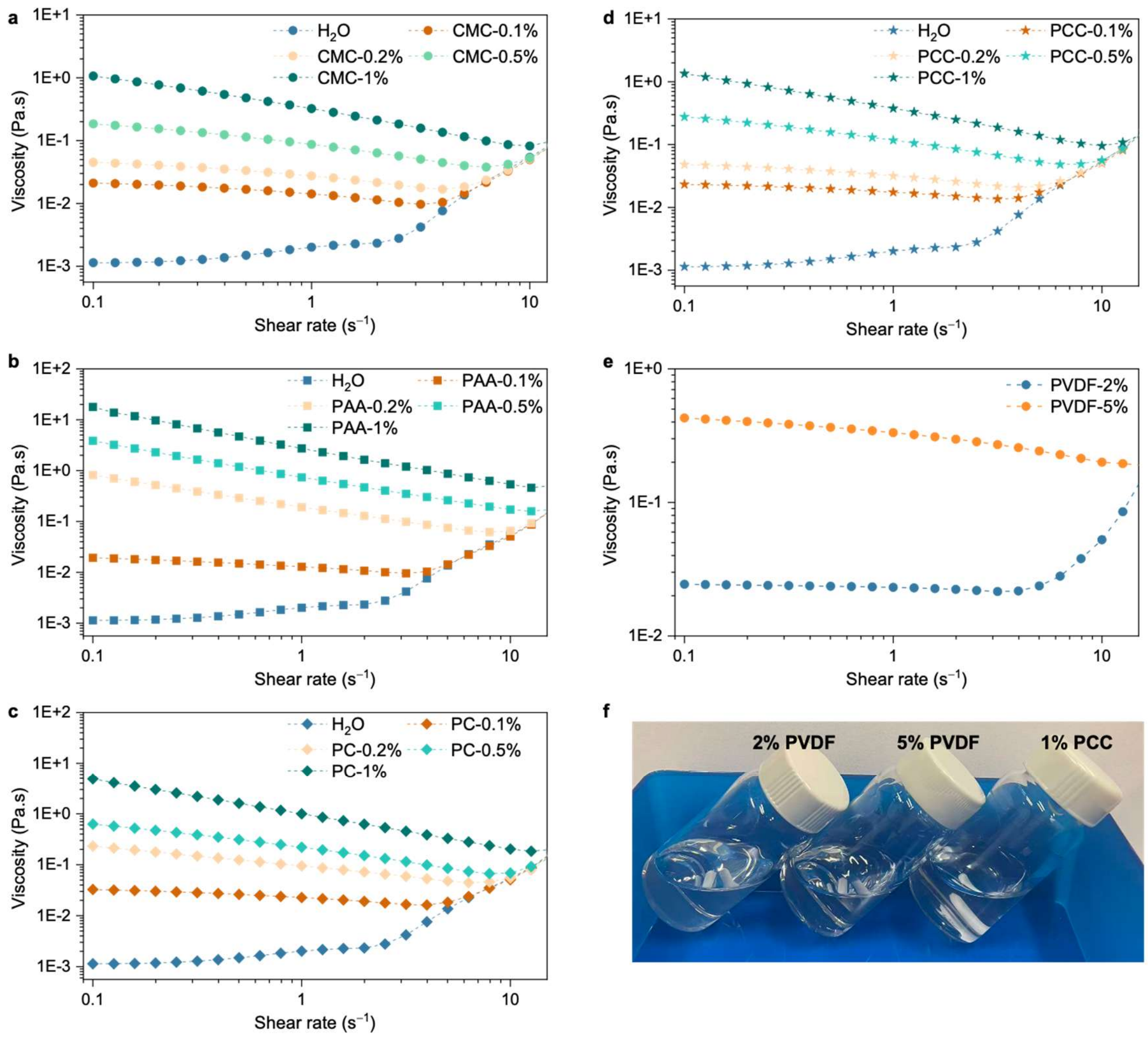

Supplementary Figure 5: **Viscosity behavior of different binder solutions and dispersion appearance.** Viscosity of aqueous binder solutions as a function of shear rate at different concentration (wt%) for (**a**) CMC, (**b**) PAA (pH adjusted to ~6 using LiOH), (**c**) PC (pH adjusted to ~6 using LiOH), and (**d**) PCC. For water-soluble binder systems, the viscosity of pure water is included as a reference. (**e**) Viscosity of PVDF solutions in NMP at 2 wt% and 5 wt%. (**f**) Photographs of PVDF dispersions in NMP (2 wt% and 5 wt%) and PCC in water (1 wt%).

Supplementary Figure 5 provides a comparative, concentration-dependent overview of the viscosity and dispersion behavior of individual binder components (CMC, PAA, PC), the crosslinked PCC system, and conventional PVDF solutions. For water-processable binders, viscosity increases with concentration as a result of polymer chain entanglement and

intermolecular interactions. CMC exhibits a pronounced viscosity increase even at low concentrations, consistent with its rigid polysaccharide backbone. PAA and PC, after pH adjustment to ~6, show more gradual viscosity growth, reflecting improved solubility and partial chain expansion associated with carboxylate formation. The PCC system displays intermediate viscosity behavior relative to its individual components, maintaining moderate viscosity at low concentrations.

In contrast, PVDF solutions in NMP exhibit higher turbidity and increased viscosity with concentration. The optical images in **Supplementary Figure 5f** highlight differences in dispersion appearance between PVDF in NMP and PCC in water, consistent with differences in polymer-solvent interactions and dispersion states under the respective processing conditions.

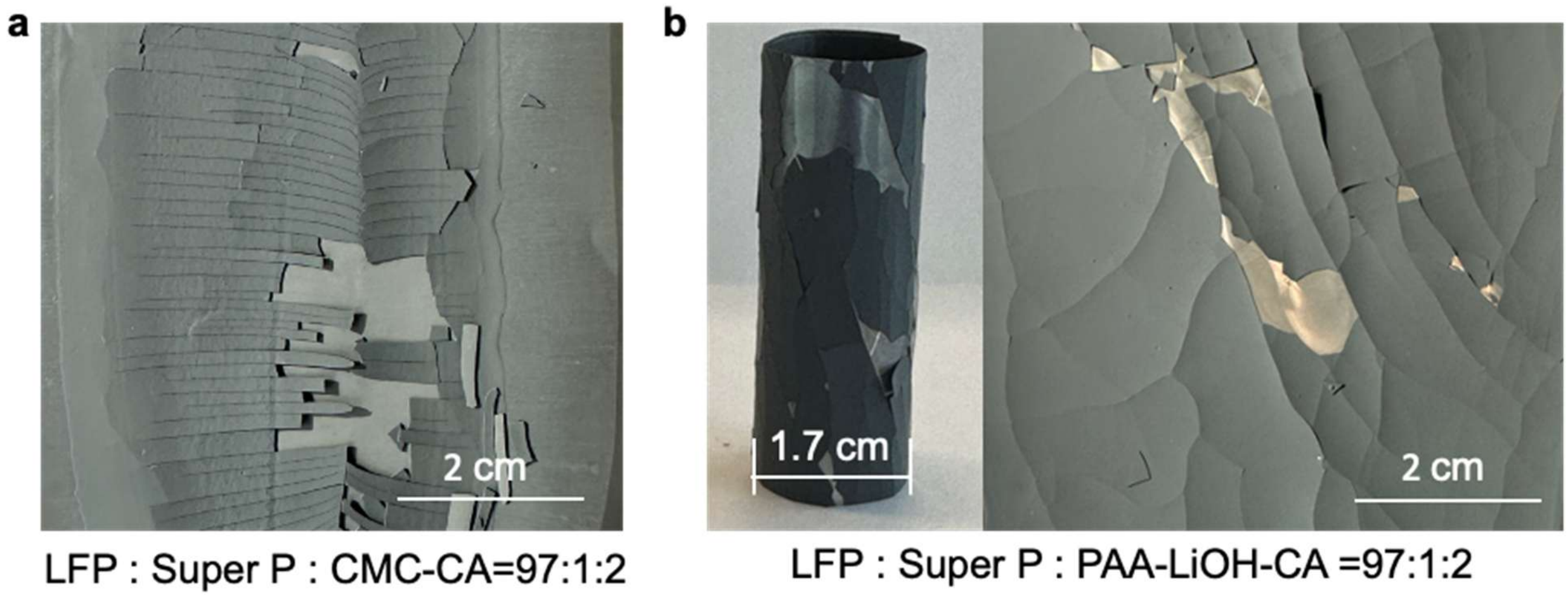

Supplementary Figure 6: **Photographs and corresponding planar views of LFP electrodes using different binder systems**. **a**, CMC-CA, **b**, PAA-LiOH-CA. All electrodes were prepared with a fixed composition of LFP: Super P: Binder = 97: 1 : 2 by weight and cast on carbon-coated Al foil under identical conditions.

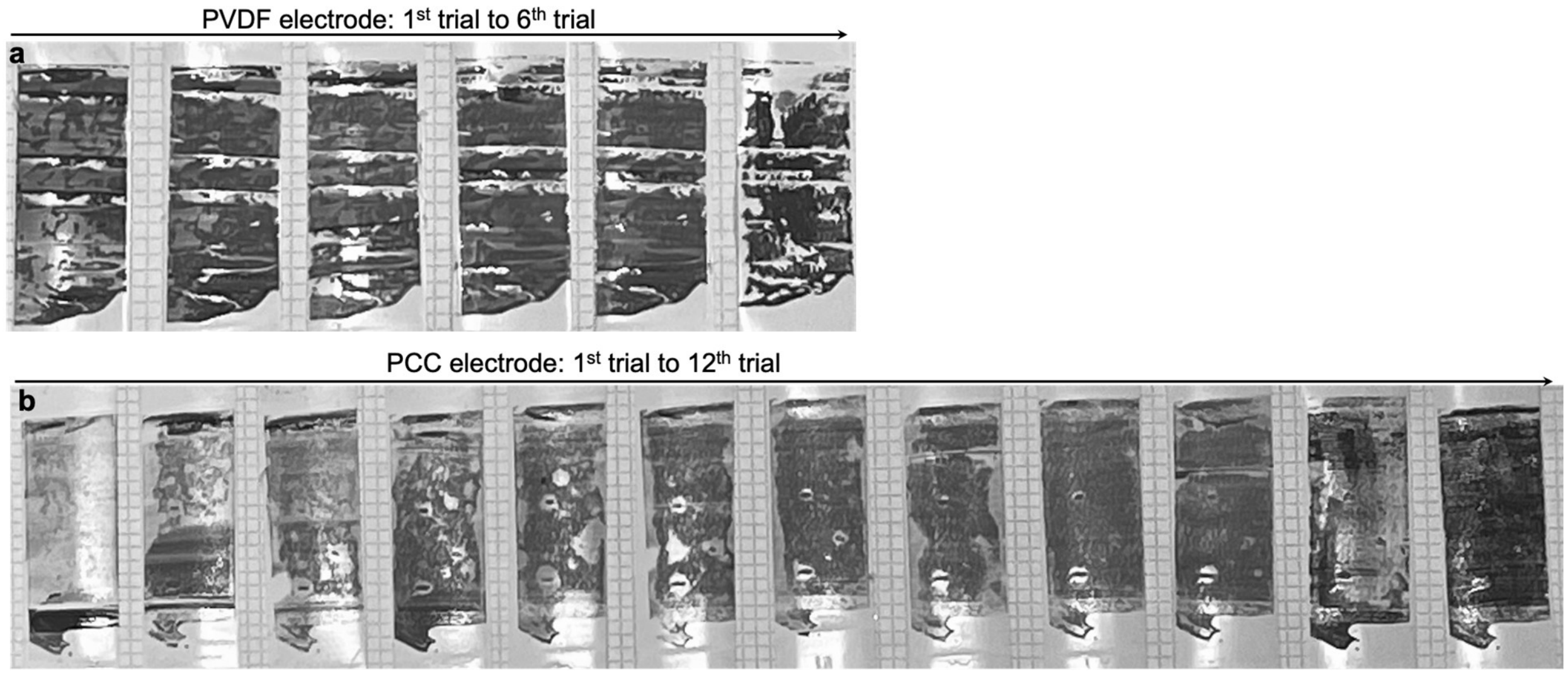

Supplementary Figure 7: Tape-peeling adhesion of LFP electrodes with different binders. Representative photographs of the adhesive tape after a peeling test from coated LFP electrodes (97 wt.% LFP, 1 wt.% super P, and 2 wt.% binder). **a**, PVDF electrode. **b**, PCC electrode. Dark regions on the tape correspond to material detached from the electrode; greater coverage signifies poorer adhesion to the Al current collector and/or cohesive failure within the electrode layer. Residue patterns differ markedly among binders, with the PCC-based electrodes showing visibly lower detachment than formulations with PVDF. Images were acquired under identical lighting.

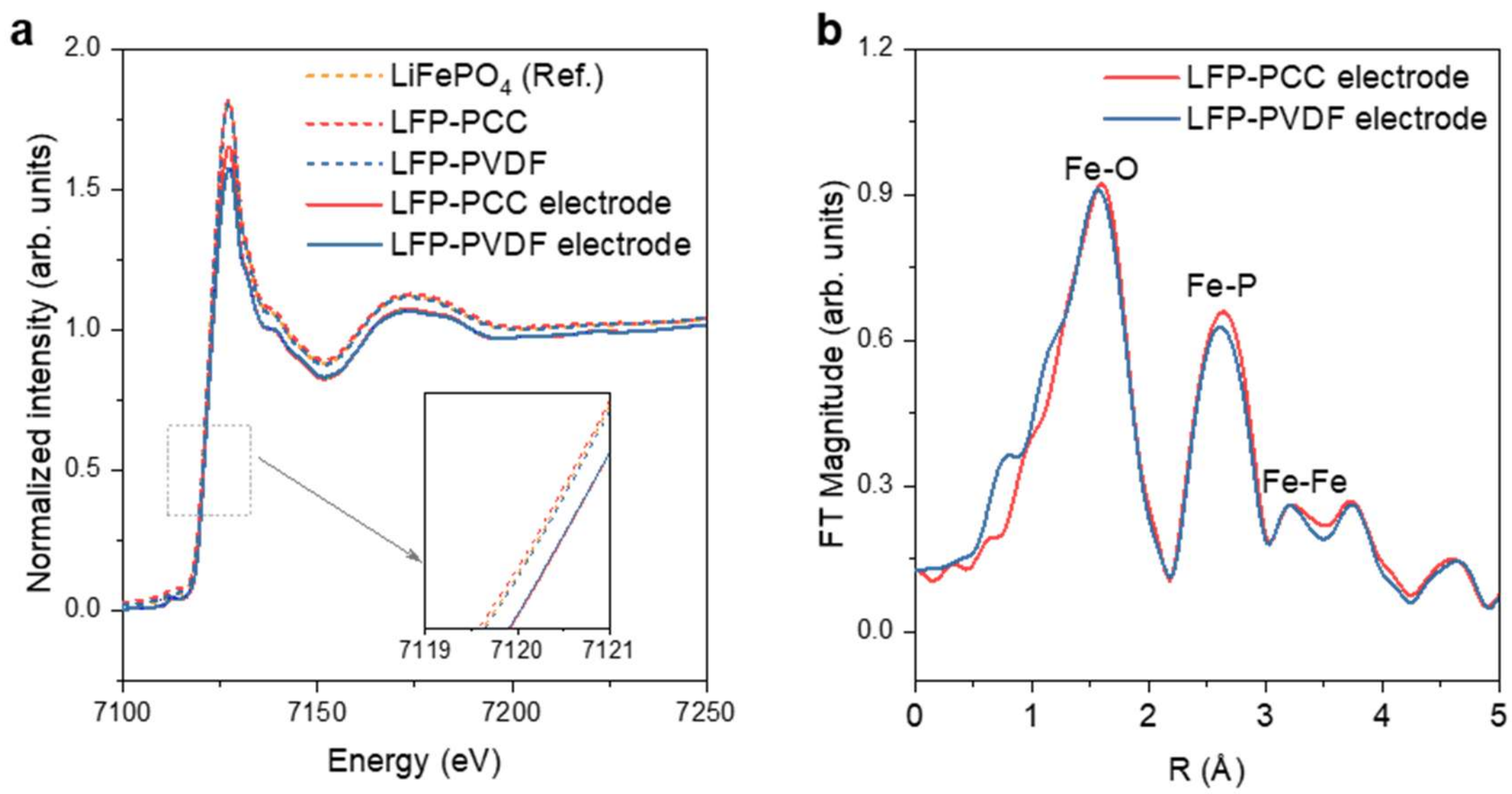

Supplementary Figure 8: Fe K-edge X-ray absorption near-edge structure (XANES) spectra and Fourier-transformed extended X-ray absorption fine structure (EXAFS) spectra of LFP samples prepared with different binders. a, Normalized Fe K-edge XANES spectra of $LiFePO_4$ reference, LFP powders (treated with PCC or PVDF), and corresponding LFP-PCC and LFP-PVDF electrodes after immersed into electrolyte, with the inset showing an enlarged view of the absorption-edge region. **b,** Magnitudes of the Fourier-transformed (FT) Fe K-edge EXAFS spectra of the LFP-PCC and LFP-PVDF electrodes.

XANES spectra reveal that all samples exhibit a characteristic absorption edge near ~7125 eV (**Supplementary Fig. 8a**), consistent with the Fe^{2+} oxidation state in olivine-structured LFP. The white-line intensities and overall spectral shapes of LFP-PCC and LFP-PVDF electrodes closely resemble that of the pristine LFP reference, indicating that neither binder system induces significant changes to the Fe valence state or the local electronic structure. The similarity between powder and electrode (with electrolyte) spectra further suggests that the electrode processing conditions, including aqueous-based slurry preparation with PCC and immersion with electrolyte, do not compromise the redox-active center of LFP. The radial distribution functions derived from Fourier-transformed extended X-ray absorption fine structure (EXAFS) spectra show well-defined peaks corresponding to Fe-O (~1.7 Å), Fe-P (~2.7 Å), and Fe-Fe (~3.2 Å) scattering paths (**Supplementary Fig. 8b**). Notably, the LFP-PCC electrode exhibits nearly identical peak

positions and amplitudes compared to its PVDF-based counterpart, confirming the maintenance of medium-range order within the olivine lattice. A slightly attenuated first-shell peak (Fe-O) in the PCC sample may indicate minor increases in local disorder, potentially due to differences in particle interactions during aqueous electrode processing. Nevertheless, the overall EXAFS profiles affirm that the PCC binder enables a comparable structural environment to conventional PVDF, without inducing lattice distortion or degradation.

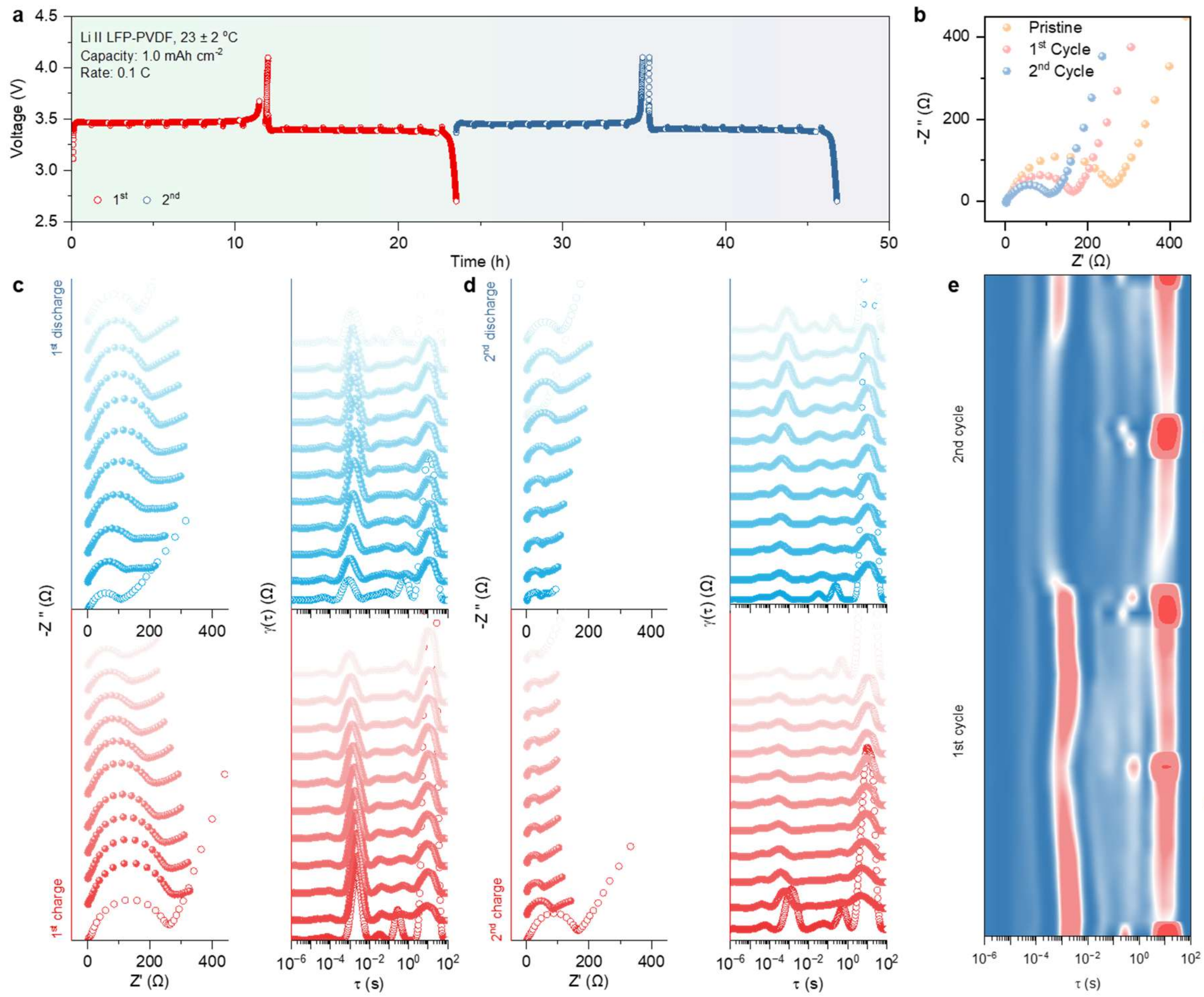

Supplementary Figure 9: *In-situ* electrochemical impedance tracking of an LFP-PVDF electrode during the first two charge/discharge cycles. **a,** Voltage-time profile of a Li||LFP-PVDF half-cell cycled at 0.1 C (1 C = 170 mA g^{-1}) and 25 ± 2 °C. Both 1st (red) and 2nd (blue) charge/discharge curves exhibit typical LFP voltage plateaus. **b**, overall Nyquist plots (Z' vs. $-Z''$) of fresh cell, the 1st cycle, and the 2nd cycle. Nyquist plots (Z' vs. $-Z''$) and corresponding impedance distribution functions $\gamma(\tau)$ extracted continuously during the (**c**) 1st and (**d**) 2nd charge (red) and discharge (blue) cycles. **e,** 2D intensity map of $\gamma(\tau)$ evolution across both cycles, highlighting the accumulation of high-resistance features at specific relaxation times. The increasing peak width and intensity suggest deteriorating interfacial kinetics and rising transport limitations.

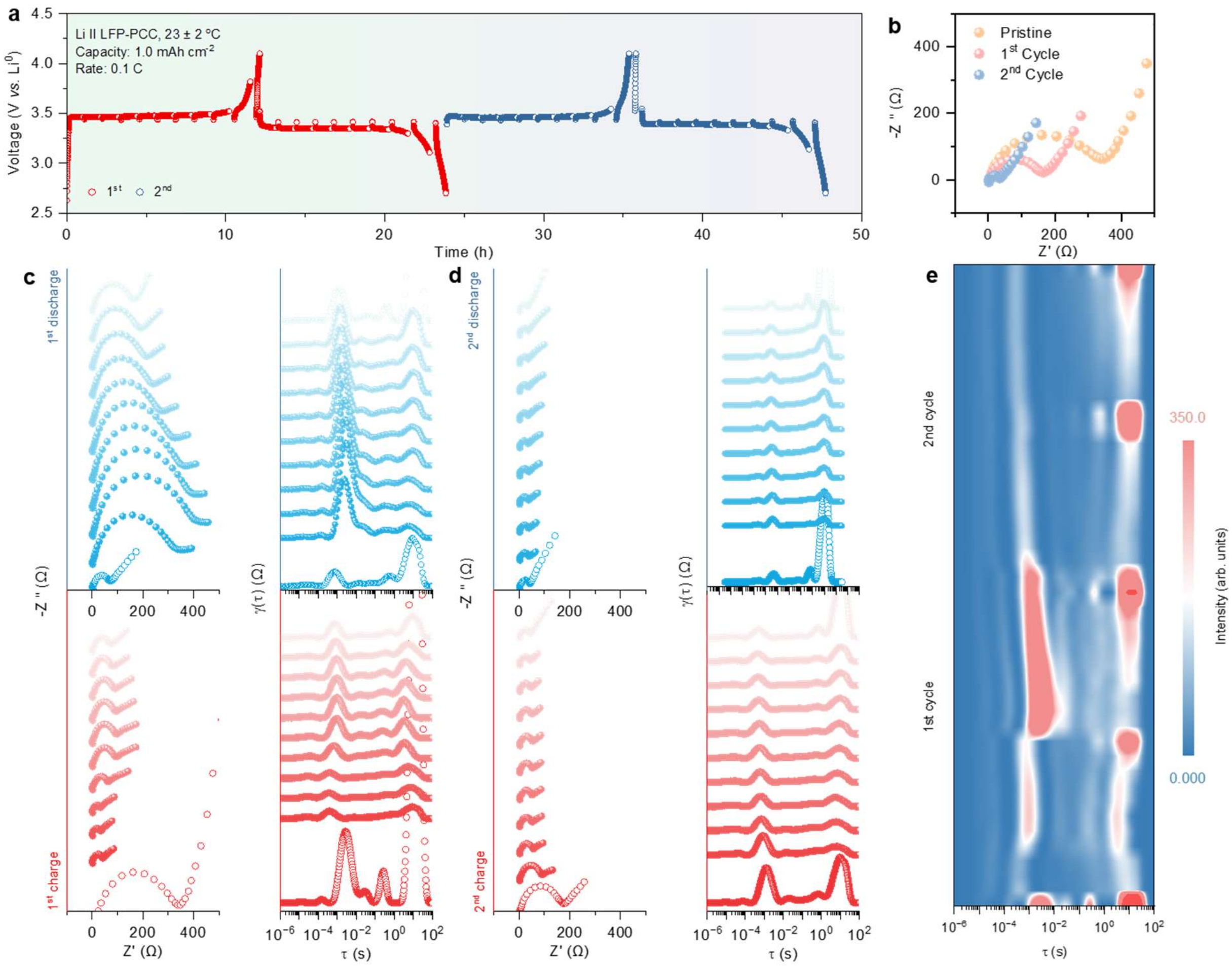

Supplementary Figure 10: *In-situ* electrochemical impedance tracking of an LFP-PCC electrode during the first two charge/discharge cycles. a, Voltage-time profile of a Li||LFP-PCC half-cell cycled at 0.1 C (1 C = 170 mA g^{-1}) and 25 ± 2 °C. The cell shows smooth and symmetric charge/discharge plateaus with a gradual voltage tail near the end of each step, indicating mild polarization buildup and good electrode kinetics. **b**, overall Nyquist plots (Z′ vs. -Z″) of fresh cell, the 1st cycle, and the 2nd cycle. Nyquist plots (Z′ vs. -Z″) and corresponding impedance distribution functions γ(τ) collected during the (**c**)1st and (**d**) 2nd charge (red) and discharge (blue) cycles. **e,** 2D intensity map of γ(τ) evolution shows fewer high-intensity regions compared to PVDF, confirming the suppression of slow relaxation processes and interfacial resistance accumulation.

In this analysis, the low-frequency (LF, $10^{-1} < \tau < 10^{0}$ s) process is assigned to the charge-transfer process associated with the Faradaic reaction at the electrode/electrolyte interface, while the mid-

frequency (MF, $10^{-4} < \tau < 10^{-2}$ s) process is assigned to the cathode electrolyte interphase[6]. Notably, the LFP-PCC electrode exhibits consistently lower charge-transfer resistance and cathode electrolyte interphase resistance values than its PVDF-based counterpart throughout the cycling process (**Supplementary Figs. 9-10**). Moreover, the PCC-based electrode demonstrates more stable impedance evolution and reduced accumulation of interfacial resistance, particularly after the first cycle (**Supplementary Fig. 10**), suggesting the formation of a more stable cathode electrolyte interphase, which facilitates Li^{+} transport across the cathode/electrolyte interface.

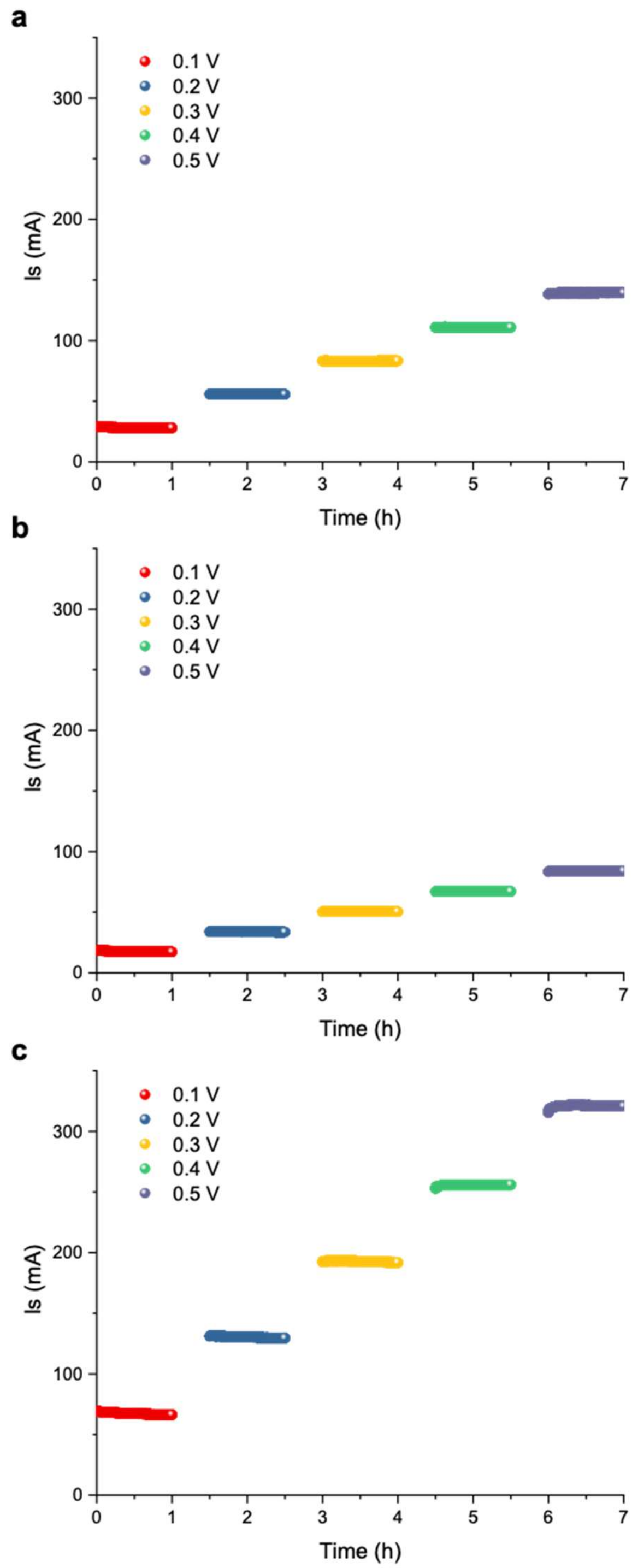

Supplementary Figure 11: Electronic conductivity measurements at 25 ± 2 °C.
Electronic conductivity measurement for (**a**) LFP-PCC, (**b**) LFP-PVDF, and (**c**) PLFP, measured by DC polarization method in the constant voltage range from 0.1 to 0.5 V.

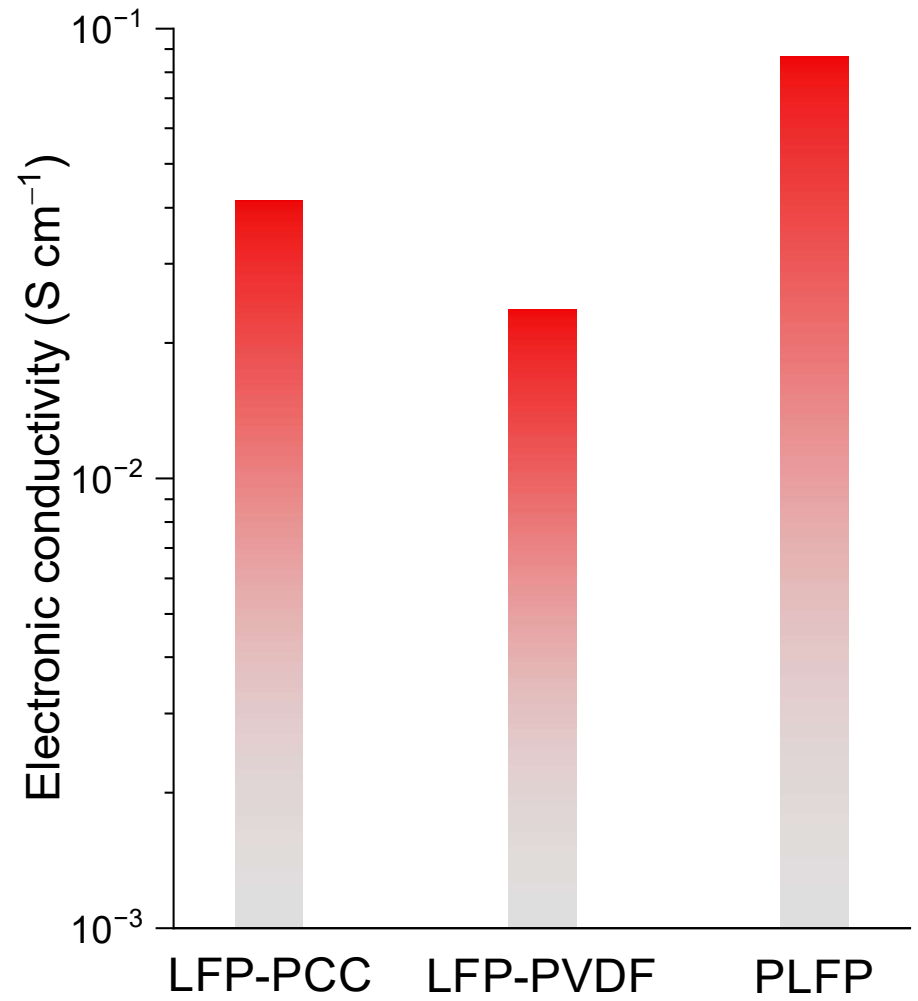

Supplementary Figure 12: Electronic conductivity comparison of PLFP, LFP-PCC, and LFP-PVDF. Samples were prepared with a fixed composition of LFP: Binder = 98: 2 by weight.

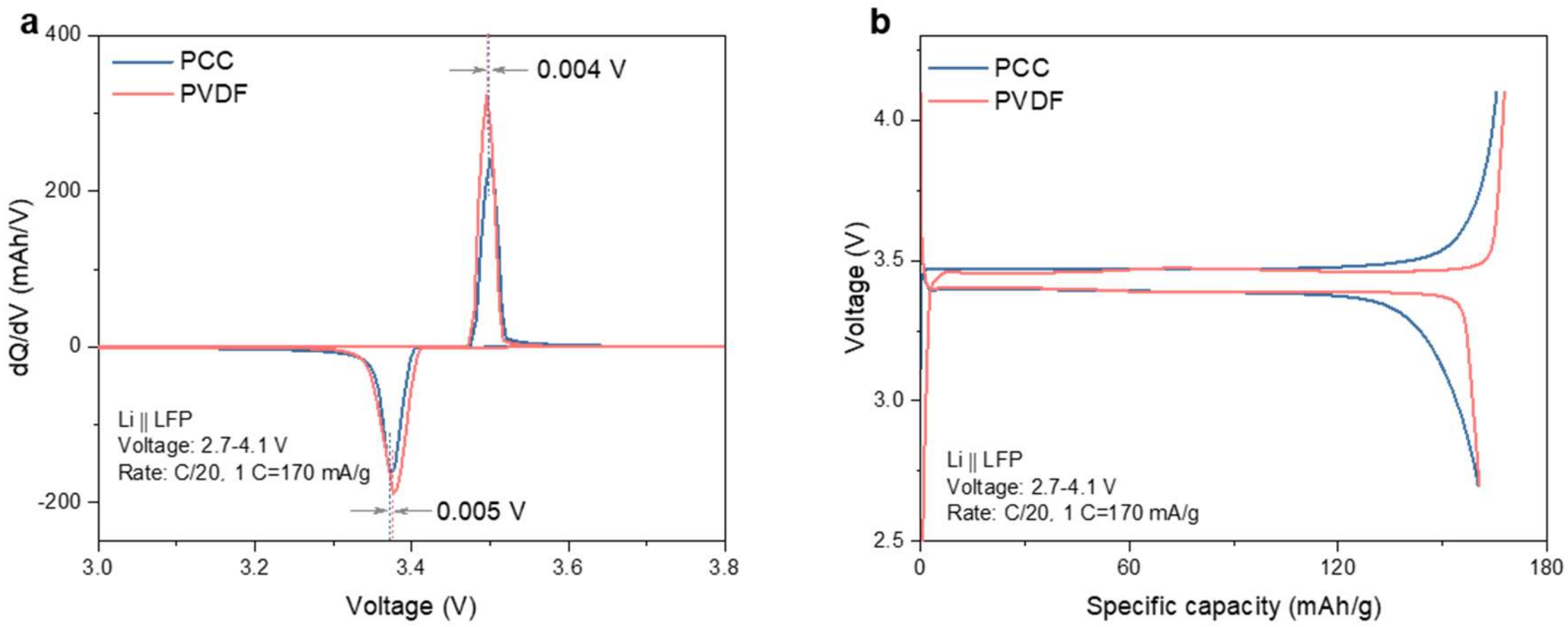

Supplementary Figure 13: Polarization analysis of Li||LFP cells with PVDF and PCC binders. a, Differential capacity (dQ/dV) curves and (**b**) corresponding galvanostatic voltage-capacity profiles of PCC- and PVDF-based electrodes during the 1st cycle at C/20 (1 C = 170 mA g^{-1}) and 25 ± 2 °C.

Both PCC and PVDF display the LFP two-phase Fe^{2+}/Fe^{3+} reaction near 3.45 V with similar peak positions in dQ/dV, indicating unchanged thermodynamics of the olivine redox couple. The PCC trace shows a broader, lower-amplitude dQ/dV peak and an earlier plateau tilt voltage-capacity profiles (≈120 mAh g^{-1}), whereas PVDF maintains a flatter plateau to ≈160 mAh g^{-1} before a sharper drop. Because the final discharge capacities are comparable and PCC shows a lower charge-transfer resistance than PVDF (**Supplementary Figs. 9, 10**), these differences do not stem from sluggish interfacial kinetics or loss of active material. This "broad-peak/early-tilt" signature has been widely reported in aqueous-processed electrodes employing CMC, PAA, or alginate binders[7], yet its mechanistic origin remains poorly understood. Resolving how polymer network chemistry governs these coupled mechanical and transport effects will be a central focus of future study.

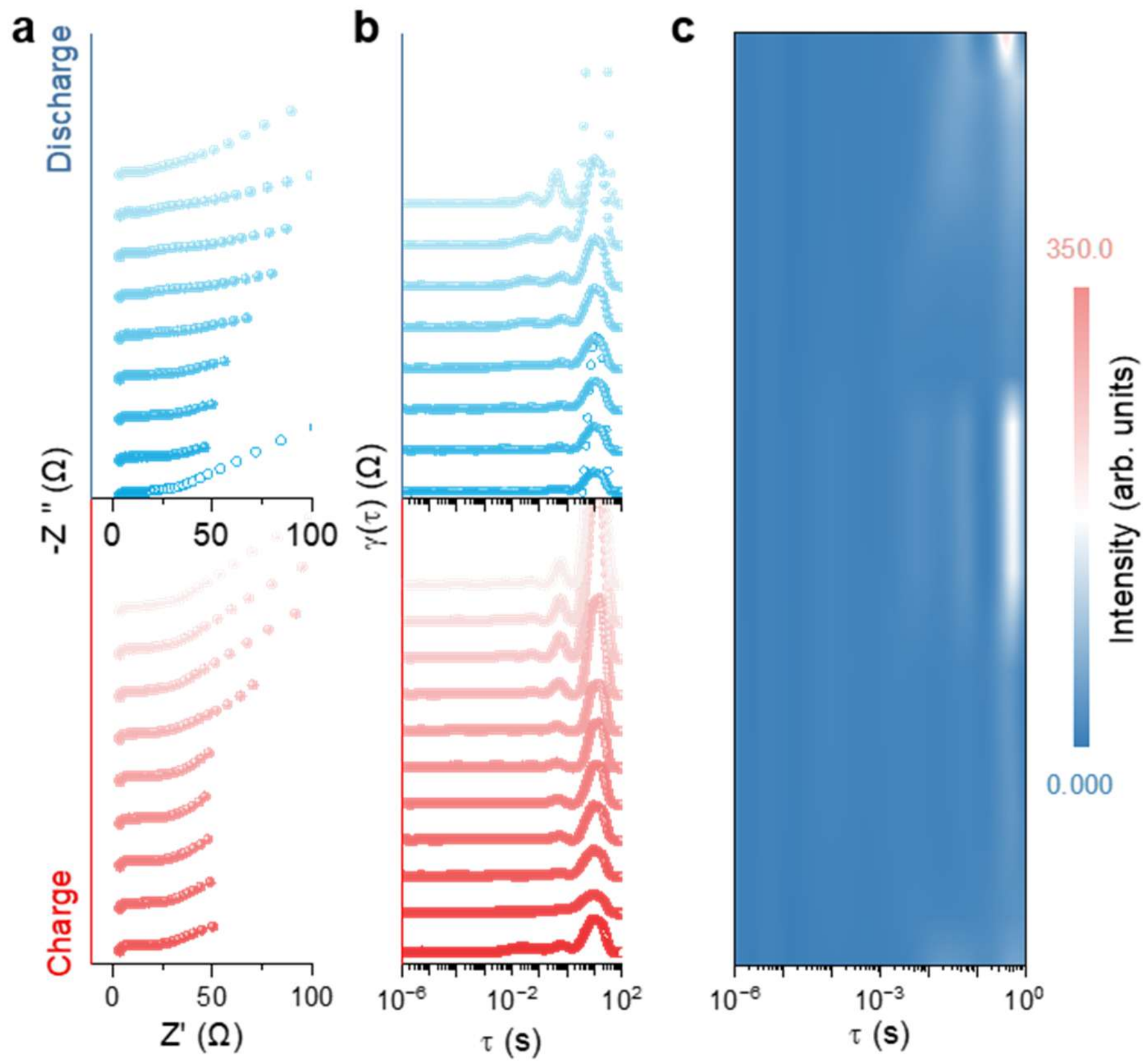

Supplementary Figure 14: *In-situ* EIS and DRT analysis of the LFP-PCC electrode after 1000 cycles at C/3 (1 C = 170 mA g^{-1}) and 25 ± 2 °C. **a**, Nyquist plots collected at different states of charge during charge and discharge process, showing minimal impedance growth. **b**, Corresponding DRT spectra highlighting a narrow mid-frequency, peak with negligible low-frequency growth, indicative of stable charge-transfer kinetics and unimpeded ionic transport. **c**, Two-dimensional DRT intensity map as a function of voltage.

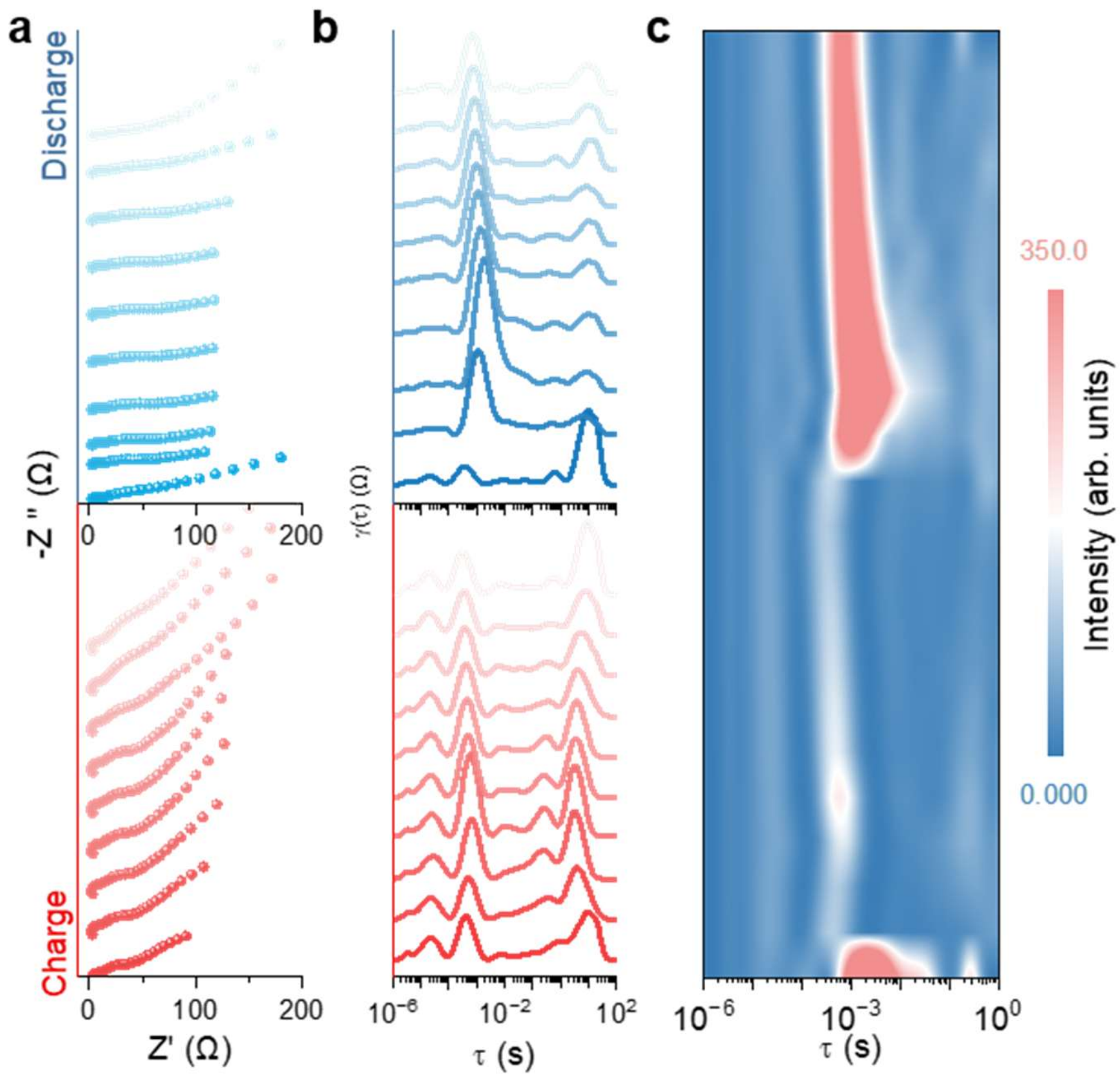

Supplementary Figure 15: *In-situ* EIS and DRT analysis of the LFP-PVDF electrode after 710 cycles at C/3 (1 C = 170 mA g^{-1}) and 25 ± 2 °C. **a**, Nyquist plots collected at different states of charge during charge and discharge, showing larger impedance arcs and more pronounced charge-discharge hysteresis compared to the PCC-based electrode. **b**, Corresponding DRT spectra featuring a broader and more intense mid-frequency peak and a pronounced LF tail, indicating elevated charge-transfer resistance and aggravated ionic transport limitations. **c**, Two-dimensional DRT intensity map as a function of voltage.

In-situ EIS with DRT deconvolution reveals that LFP electrodes using the PCC binder retain low impedance across the state of charge after 1000 cycles (**Supplementary Figure 14**). The DRT spectra exhibit a narrow low-frequency growth, indicating preserved charge-transfer pathways. By contrast, PVDF electrodes show larger Nyquist arcs and a broadened mid-frequency peak that shifts to longer relaxation times, accompanied by a pronounced low-frequency tail, consistent with interfacial slowdown and transport bottlenecks (**Supplementary Figure 15**).

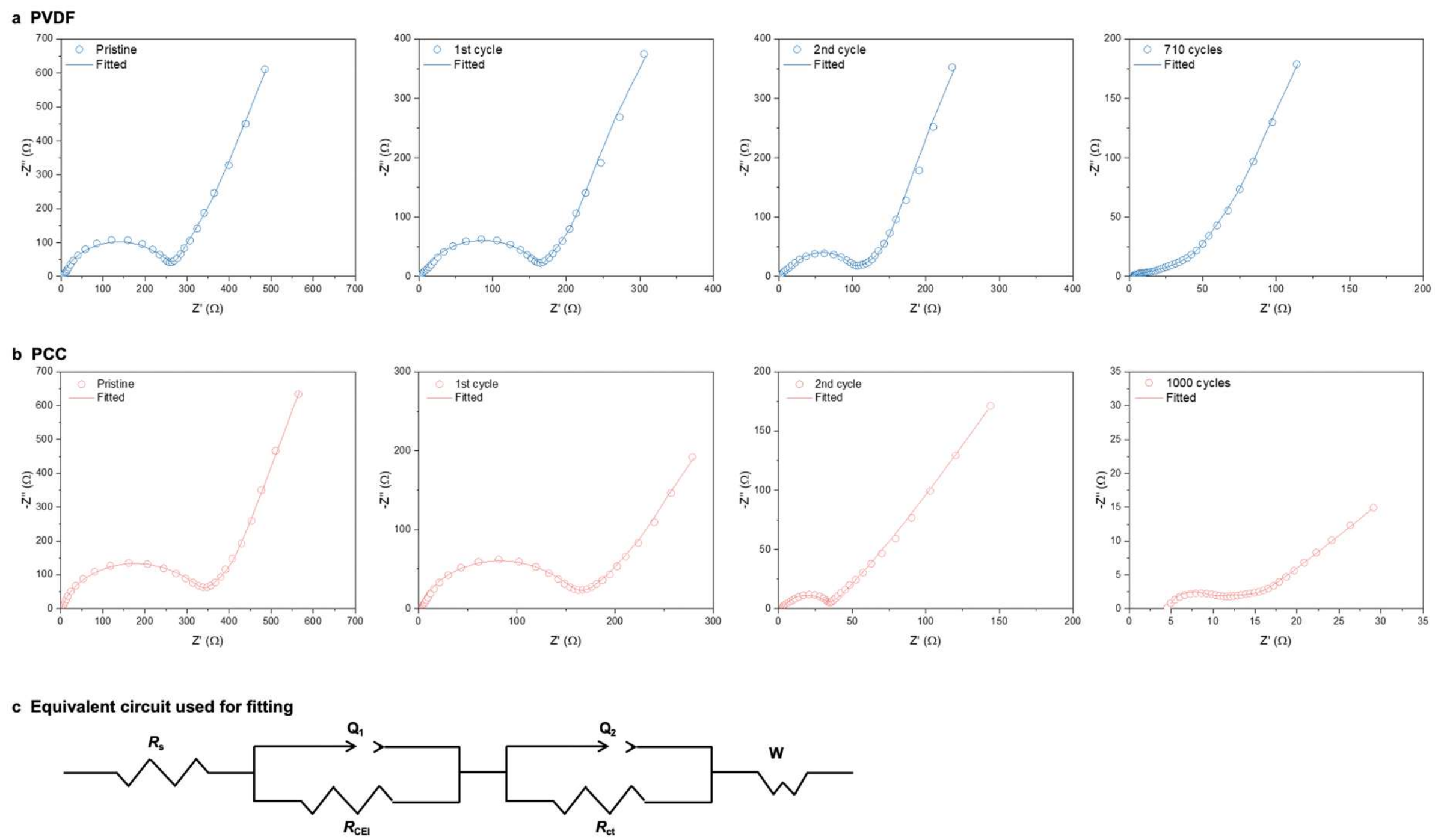

Supplementary Figure 16: Experimental Nyquist plots and corresponding equivalent-circuit fits. Experimental electrochemical impedance spectra (symbols) and corresponding equivalent-circuit fits (solid lines) for **(a)** PVDF and **(b)** PCC electrodes measured in the pristine state, after cycles 1 and 2, and at the end of life (left to right). Cells were cycled at C/3 (1 C = 170 mA g^{-1}) and 25 ± 2 °C. **(c)** Equivalent circuit used for impedance fitting. The extracted fitting parameters are summarized in Supplementary Table 5.

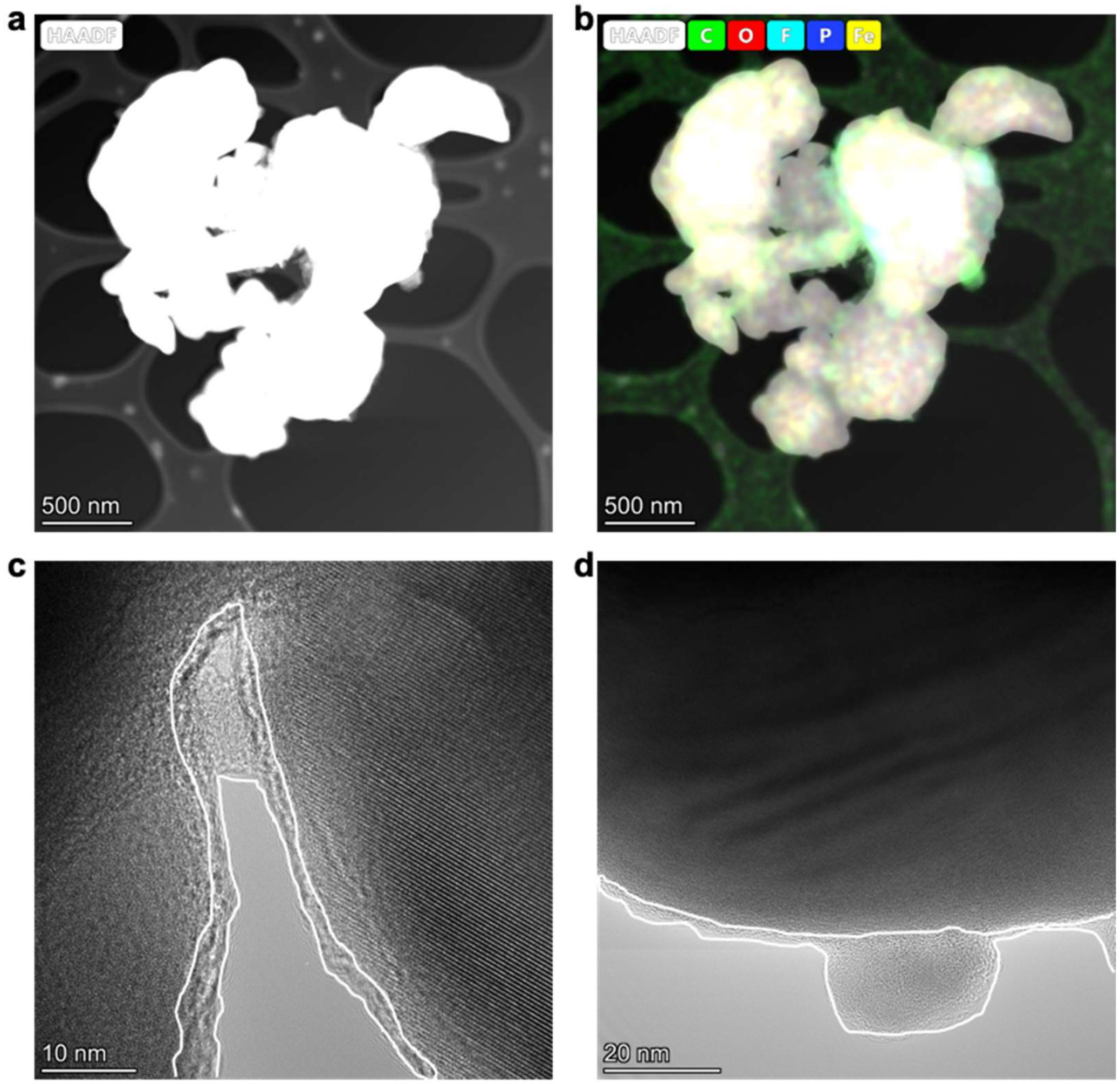

Supplementary Figure 17: TEM and EDS analysis of the LFP-PCC positive electrode after 1000 cycles. The electrode was harvested after 1000 cycles at C/3 (1 C = 170 mA g^{-1}) between 2.7 and 4.1 V versus Li/Li^+ in the fully discharged state at 25 ± 2 °C. **a**, HAADF-STEM image showing aggregated secondary particles with irregular morphology after extended cycling. **b**, Corresponding HAADF-EDS elemental maps of C, O, F, P, and Fe. (**c, d**) High-resolution TEM images of representative particle surfaces, showing a thick (~2-10 nm) amorphous surface layer. The surface layer was highlighted by while lines.

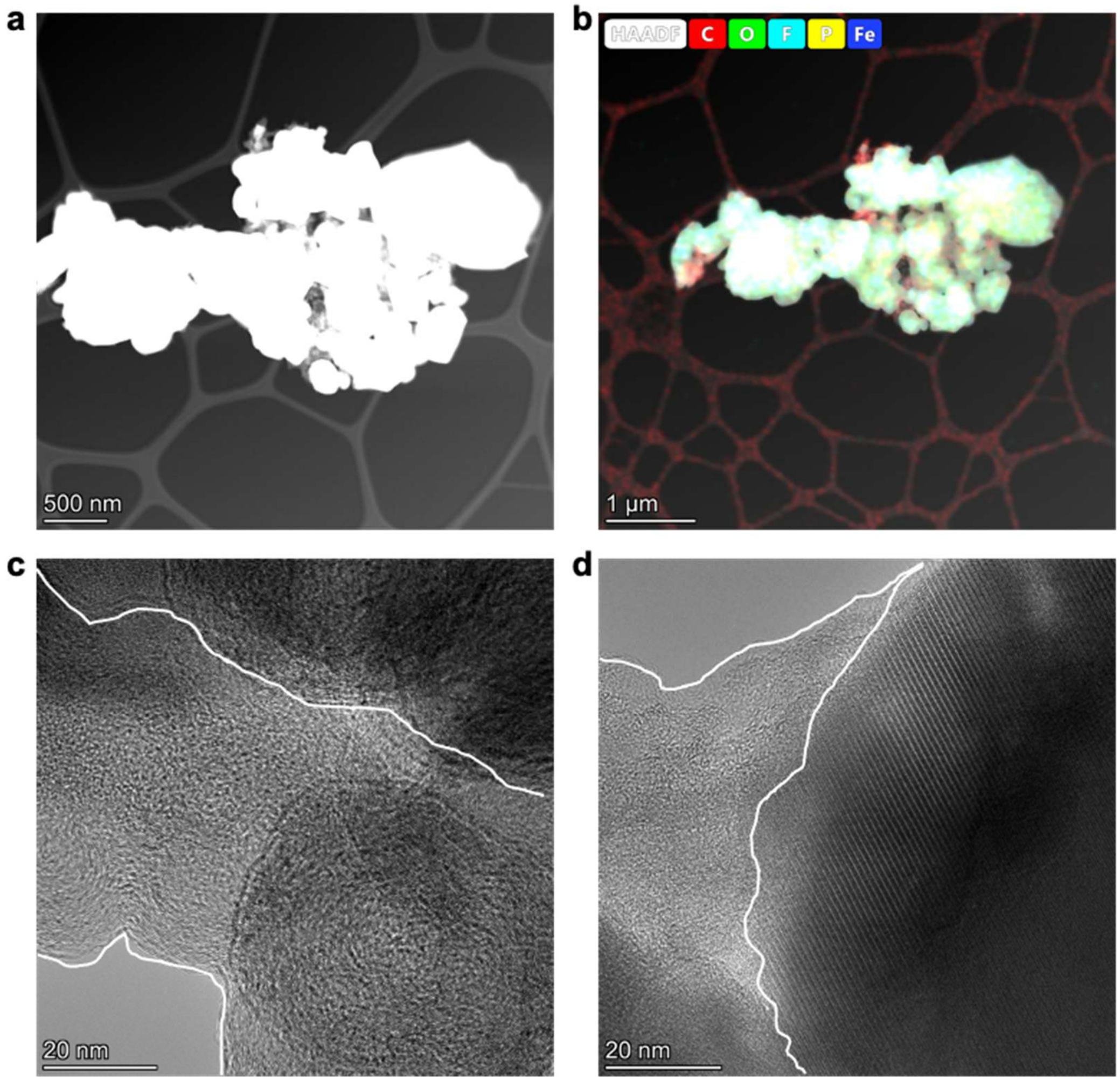

Supplementary Figure 18: TEM and EDS analysis of the LFP-PVDF electrode after 710 cycles. The electrode was harvested after 710 cycles at C/3 (1 C = 170 mA g^{-1}) between 2.7 and 4.1 V versus Li/Li^+ in the fully discharged state at 25 ± 2 °C. **a**, HAADF-STEM image showing aggregated LFP secondary particles with irregular and partially fragmented morphology after prolonged cycling. **b**, HAADF-EDS elemental maps of C, O, F, P, and Fe. (**c, d**) High-resolution TEM images showing particle surfaces covered by a thick (~5-50 nm) amorphous layer with rough texture and blurred lattice fringes at the interface. The surface layer was highlighted by white lines.

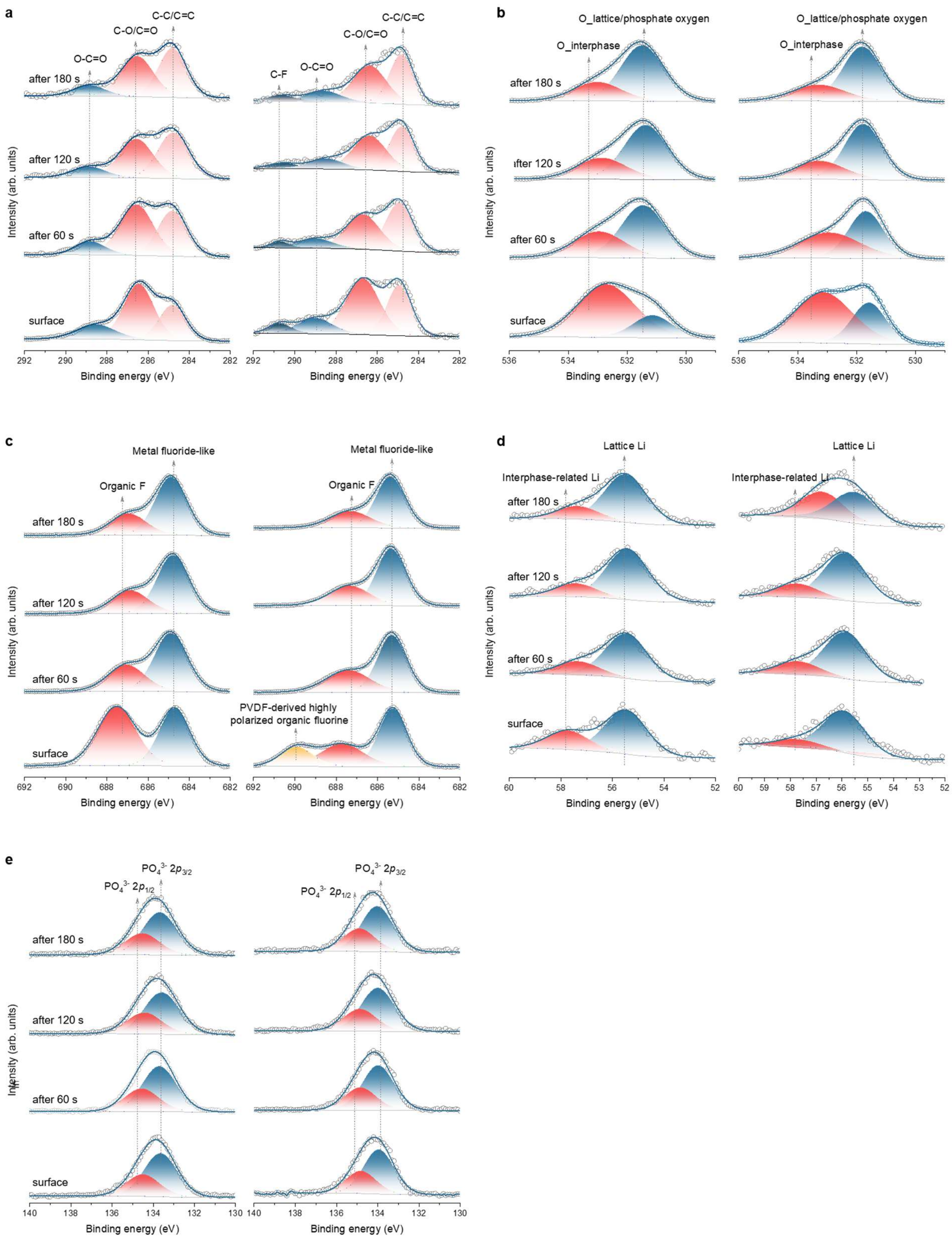

Supplementary Figure 19: Depth-resolved XPS analysis of cycled LFP electrodes with PCC and PVDF binders. The electrodes were harvested after 1000 cycles (PCC) and 710 cycles (PVDF)

at C/3 (1 C = 170 mA g^{-1}) between 2.7 and 4.1 V versus Li/Li^+ in the fully discharged state at 25 ± 2 °C. High-resolution XPS spectra of (**a**) C 1*s*, (**b**) O 1*s*, (**c**) F 1*s*, (**d**) Li 1*s*, and (**e**) P 2*p* collected from cycled LFP electrodes employing PCC (left) and PVDF (right) binders. For each panel, spectra from bottom to top correspond to increasing sputtering time, including the surface spectrum (0 s) followed by sequential Ar_{1000}^{+} gas cluster sputtering steps of 60 s per cycle (up to three cycles, total 180 s). Binding energies were calibrated using the C-C component of the C 1s peak at 284.8 eV, and spectra were fitted using physically constrained peak positions and full widths at half maximum across sputtering depths. Chemical-state assignments are discussed conservatively using grouped components, particularly for Li 1*s* and O 1*s*, due to intrinsic peak overlap and possible sputtering-induced artifacts[8]. Depth-dependent trends are interpreted qualitatively and corroborated across complementary core levels.

The C 1*s* spectra (**Supplementary Fig. 19a**) reveal distinct binder-dependent interfacial chemistries. For the PCC electrode, the surface is dominated by oxygenated carbon species (C-O, C=O, and O-C=O-related components)[9], which attenuate rapidly with sputtering, while the C-C/C=C contribution becomes increasingly prominent. In contrast, the PVDF electrode exhibits an additional $-CF_2-$ component at higher binding energy (~290.6 eV) that persists upon sputtering, indicating the presence of fluorinated polymer-derived carbon species extending beyond the outermost surface layer[10]. Such persistence of $-CF_2-$ signals is consistent with previous reports on PVDF-derived interphase residues in fluorinated binder systems.

Consistent trends are observed in the O 1*s* spectra (**Supplementary Fig. 19b**). All electrodes show a low-binding-energy component (~531 eV) attributed to lattice/phosphate oxygen from the LFP framework, together with higher-binding-energy components (~532.5-533 eV) associated with oxygenated interphase species (C-O/C=O/carbonate-related species and adsorbates)[11]. For both of the PCC and PVDF electrodes, the high-binding-energy oxygenated components decay rapidly with sputtering, revealing lattice oxygen at shallow depths. (Trends are discussed qualitatively given potential sputtering-induced artifacts.)

The F 1*s* spectra (**Supplementary Fig. 19c**) further highlights the contrasting interphase characteristics. Two principal fluorine components are resolved: a low-binding-energy component (~685 eV) associated with inorganic fluorine species (LiF-like) and a higher-binding-energy

component (~688-690 eV) corresponding to organic or polymer-derived fluorine[12]. For the PCC electrode, fluorine is largely confined to the outermost surface and diminishes rapidly with sputtering. In contrast, the PVDF electrode exhibits persistent organic fluorine and a LiF-like component that remains significant, and can become more pronounced, at subsurface depths, suggesting the formation of a fluorine-rich, salt-containing interphase beneath the surface. (Given the intrinsic overlap of Li 1s chemical states, assignments are discussed in conjunction with the F 1*s* and O 1*s* spectra.)

The Li 1*s* spectra (**Supplementary Fig. 19d**) were interpreted using grouped components due to the intrinsic overlap of Li chemical states. A lower-binding-energy contribution is associated with lattice Li in LFP, while a higher-binding-energy contribution corresponds to interphase-related Li species (LiF/carbonate-related)[13]. For the PCC electrode, the interphase-related Li contribution decreases rapidly with sputtering, consistent with a thin and readily removable surface layer. In contrast, the PVDF electrode retains a pronounced interphase-related Li contribution, which can exhibit a subsurface maximum upon sputtering, corroborating the presence of a salt-rich subsurface interphase inferred from the F 1*s* and O 1*s* spectra.

Importantly, the P 2*p* spectra (**Supplementary Fig. 19e**) remain invariant across sputtering depths and binder systems, displaying only the characteristic phosphate spin-orbit doublet (~133-134 eV)[14]. No additional phosphorus chemical states are resolved, indicating that the phosphate framework of LFP remains chemically intact after cycling. These results demonstrate that the binder-dependent differences arise primarily from interphase chemistry rather than degradation of the LFP host lattice.

Overall, the depth-resolved XPS analysis reveals that PCC promotes the formation of a thinner, oxygenated, and fluorine-poor interphase that is readily removed with sputtering, whereas PVDF leads to a fluorine- and lithium-rich subsurface interphase that persists to greater depths. These findings provide direct chemical evidence linking binder chemistry to interphase thickness, composition, and removability, with important implications for interfacial stability and recyclability.

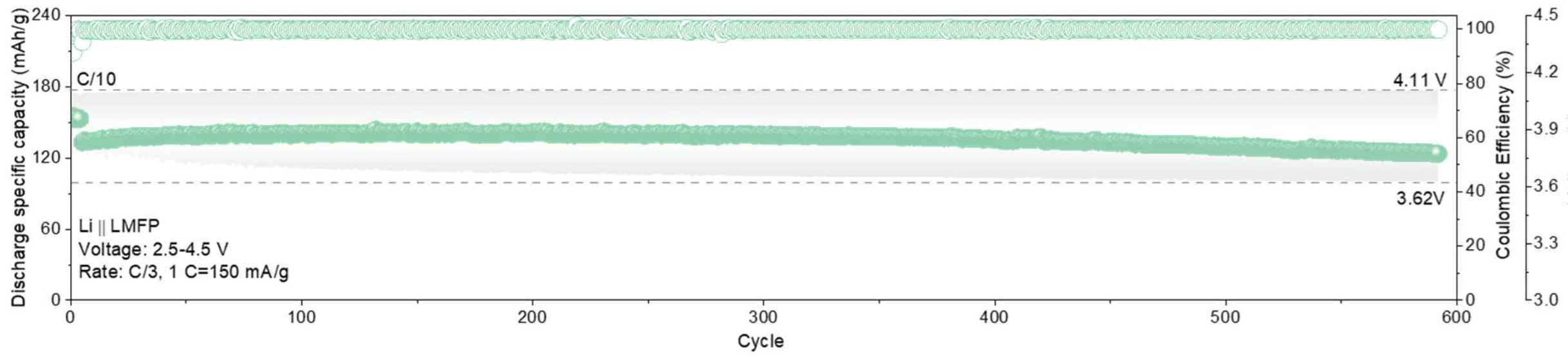

Supplementary Figure 20: Cycling performance of Li||LMFP half-cells employing PCC binders (25 ± 2 °C), measured at C/3 (1 C = 150 mA g^{-1}) with a voltage window of 2.5-4.5 V vs. Li/Li^{+}.

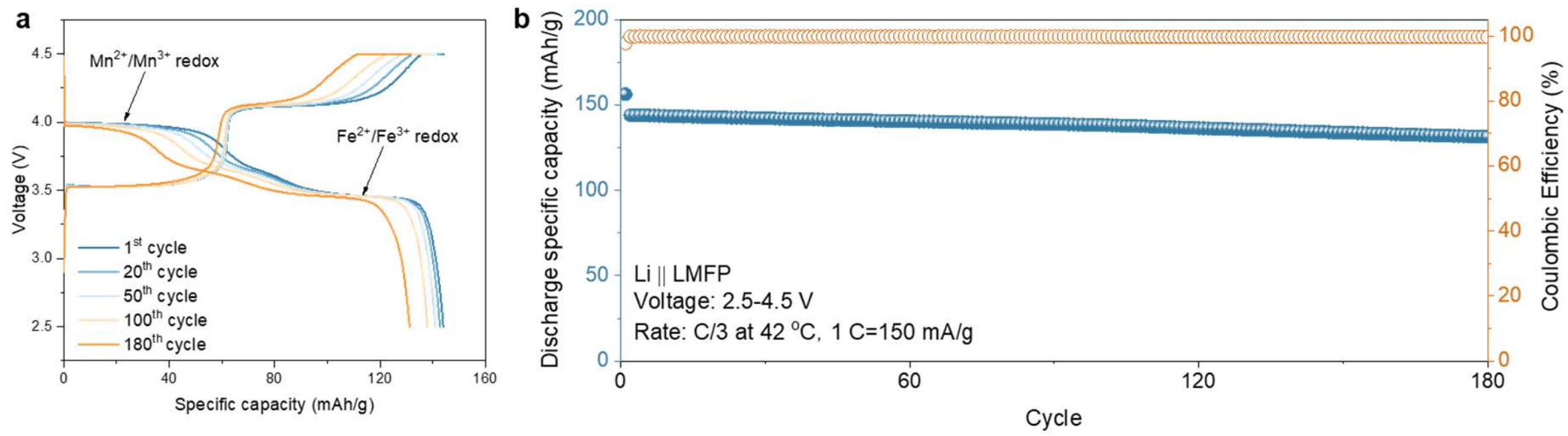

Supplementary Figure 21: Cycling performance of LMFP at 42 °C. a, Representative charge-discharge profiles and **b**, Cycling performance of LMFP at 42 °C, evaluated at C/3 (1 C = 150 mA g^{-1}) with a voltage window of 2.5-4.5 V vs. Li/Li^{+}.

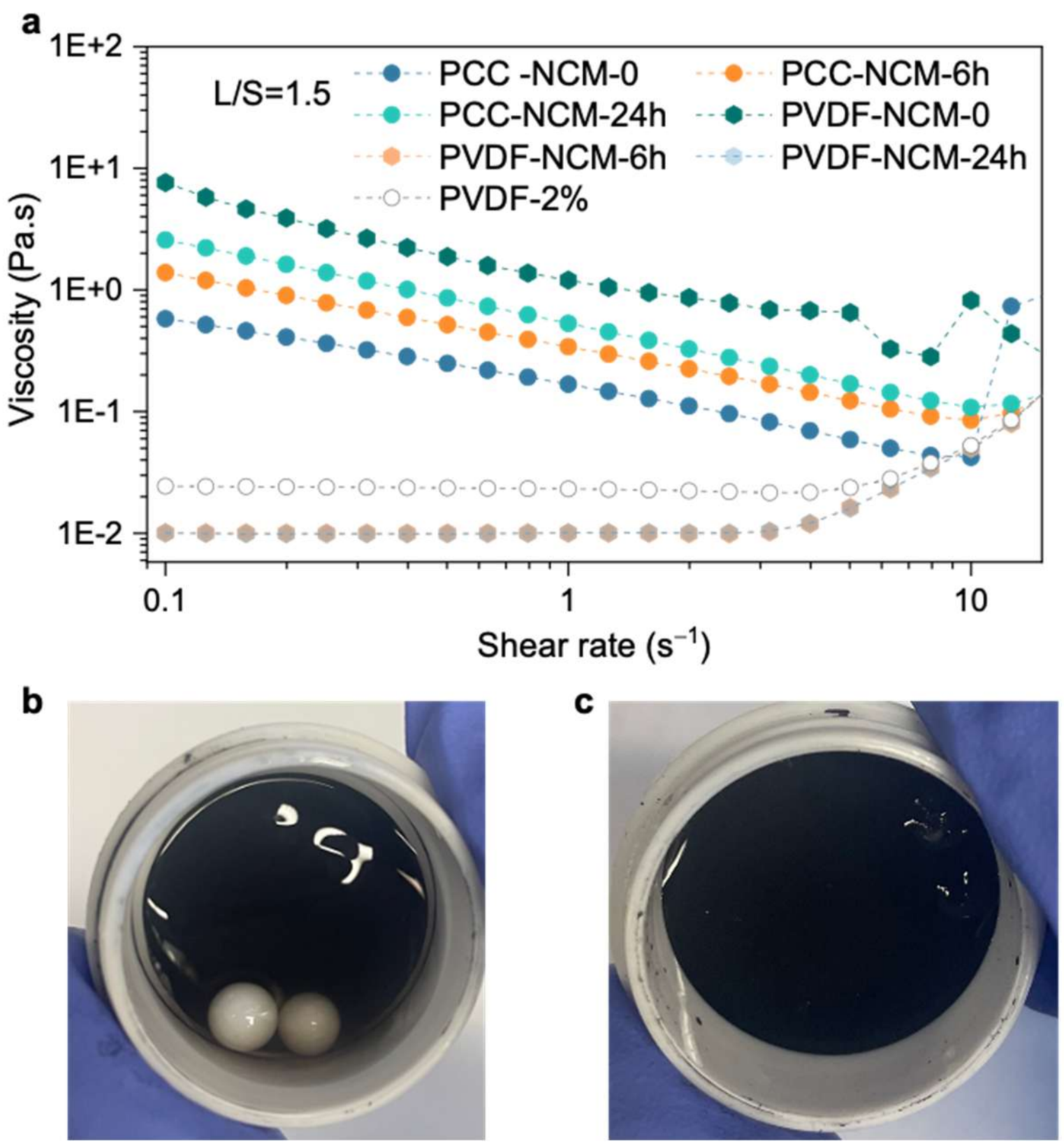

Supplementary Figure 22: Time-dependent viscosity and dispersion behavior of NCM811 slurries. a, Viscosity evolution of NCM811 electrode slurries prepared with PCC and PVDF binders as a function of holding time (0, 6, and 24 h), measured under identical formulation conditions (liquid-to-solid ratio, L/S = 1.5; carbon additive omitted to isolate binder-NCM interactions) and identical rheological measurement settings. **b, c,** Photographs of PVDF-based (b) and PCC-based (c) NCM811 slurries after 48 h of static holding. The PVDF-based slurry precipitates, leaving a clear solution on the top where the balls can be clearly seen while the PCC slurry remains homogenous without phase separation.

For PCC-based slurries, the apparent viscosity shows a modest increase with holding time, while the overall magnitude and shear-rate dependence remain largely unchanged, indicating a stable slurry microstructure with minimal time-dependent evolution. In contrast, PVDF-based slurries exhibit a pronounced decrease in viscosity with holding time (**Supplementary Fig. 22a**), reflecting progressive structural degradation during rest. After 24 h, the viscosity of the PVDF-

NCM slurry falls below that of a pure 2 wt% PVDF solution, suggesting weakened particle-binder interactions and loss of effective network formation. Consistent with the rheological results, macroscopic observations reveal visible particle agglomeration and sedimentation in the PVDF-based slurry, whereas the PCC-based slurry remains homogeneous after prolonged holding (**Supplementary Fig. 22b, c**). Collectively, these results demonstrate that the PCC binder enables robust dispersion and suspension of NCM811 particles, effectively suppressing settling and time-dependent instability.

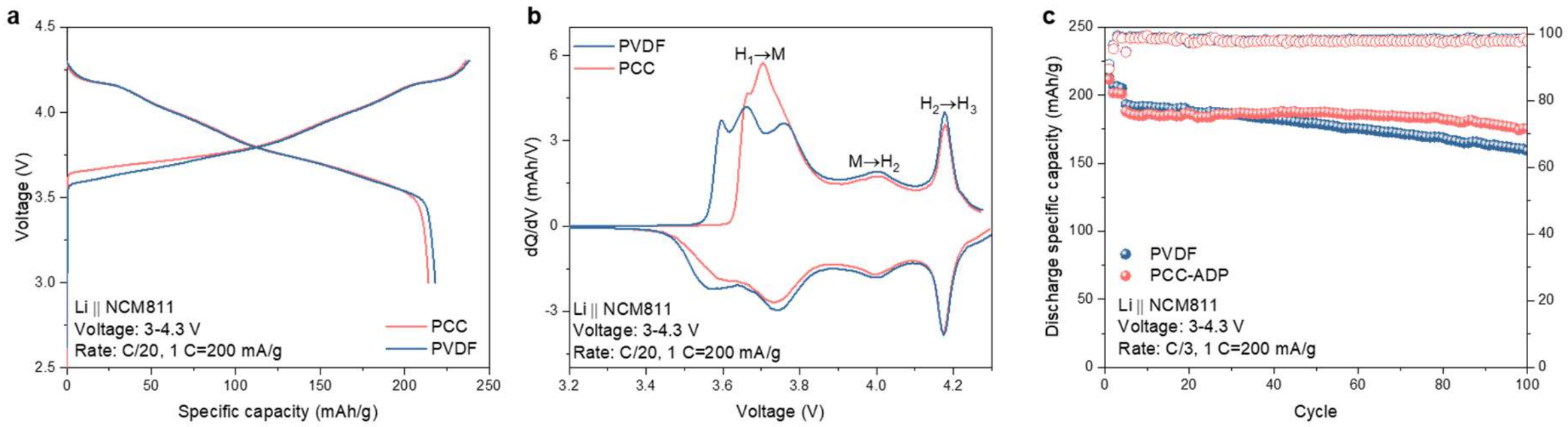

Supplementary Figure 23: Electrochemical performance comparison of NCM811 electrodes with different binders. a, Initial galvanostatic voltage-capacity profiles and corresponding (**b**) dQ/dV curves of PCC- and PVDF-based NCM811 electrodes. **c**, Long-term cycling stability comparison between these electrodes at a C-rate of C/3 (1 C = 200 mA g^{-1}) and 25 ± 2 °C.

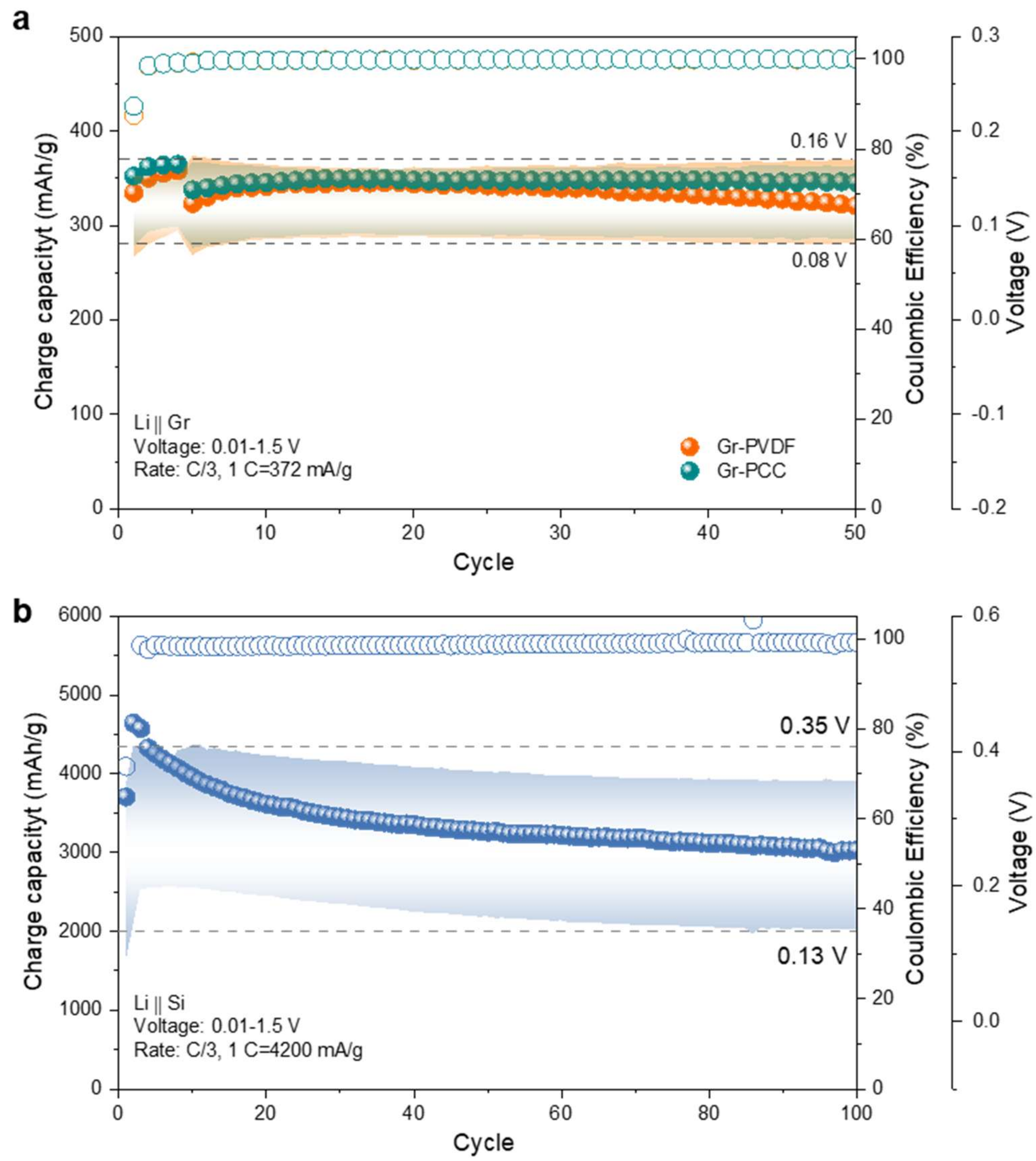

Supplementary Figure 24: Extend evaluation across negative electrode chemistries. a, Cycle performance of graphite by using PCC binder, compared with PVDF binder system (1 C = 372 mA g^{-1}, 25 ± 2 °C). **b**, Cycle stability of silicon by using PCC binder (1 C = 4200 mA g^{-1}, 25 ± 2 °C).

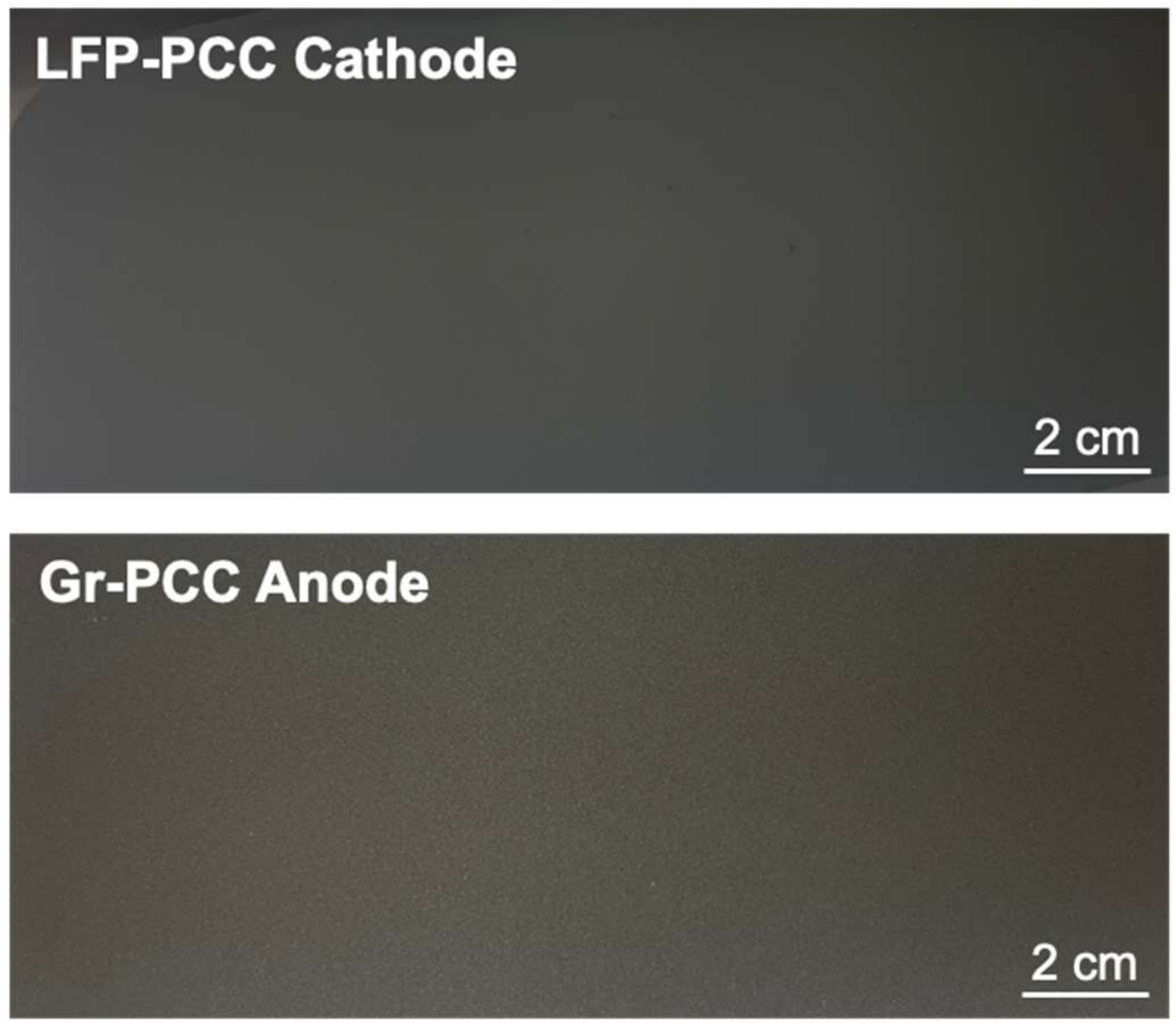

Supplementary Figure 25: Photographs of large-area LFP-PCC positive electrode and Gr-PCC negative electrode used for pouch cell assembly.

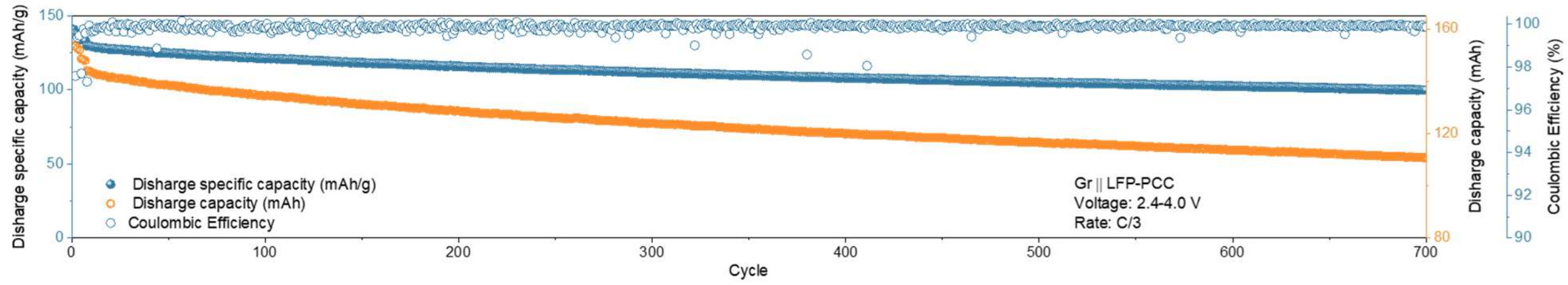

Supplementary Figure 26: Cycling performance of five layers Gr||LFP-PCC pouch cell operated at C/3 rate (1 C = 150 mA g^{-1}, 25 ± 2 °C). The discharge specific capacity (mAh g^{-1}), absolute discharge capacity (mAh), and Coulombic efficiency (%) are shown as a function of cycle number. The pouch cell has an initial capacity of 156.1 mAh.

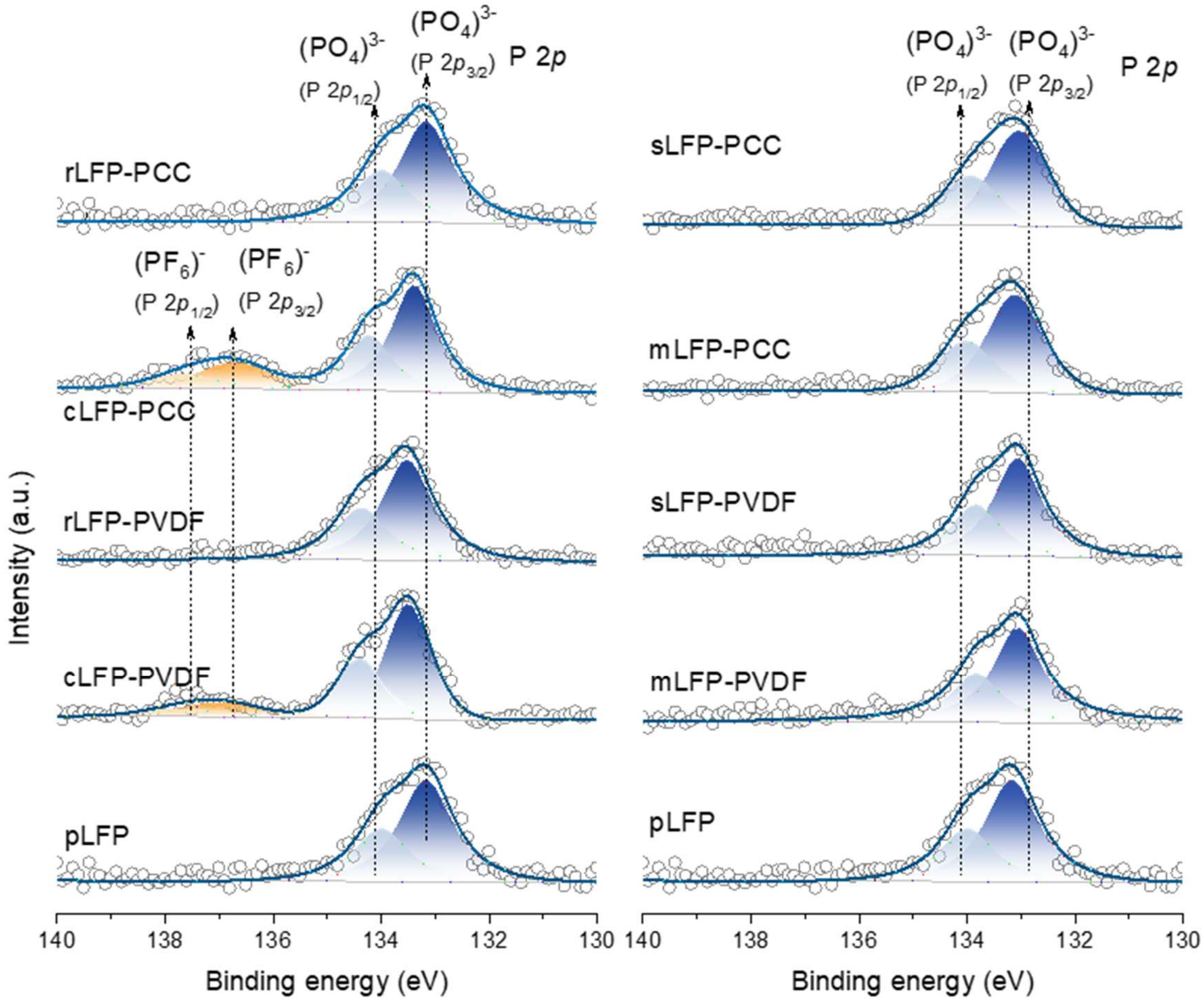

Supplementary Figure 27: P 2*p* XPS spectra of various LFP samples. cLFP-PVDF and cLFP-PCC denote cycled LFP powders obtained from PVDF- and PCC-based pouch cells, respectively. The electrodes were harvested after 100 cycles at C/3 (1 C = 150 mA g^{-1}) in the fully discharged state at 25 ± 2 °C. rLFP refers recycled LFP materials from corresponding counterparts. mLFP-PVDF and mLFP-PCC refers to mixed pristine LFP with PVDF and PCC binders in a ratio of 98:2, while sLFP refers samples that obtained from corresponding counterparts after sintered at 650 °C for 4 hours in Ar.

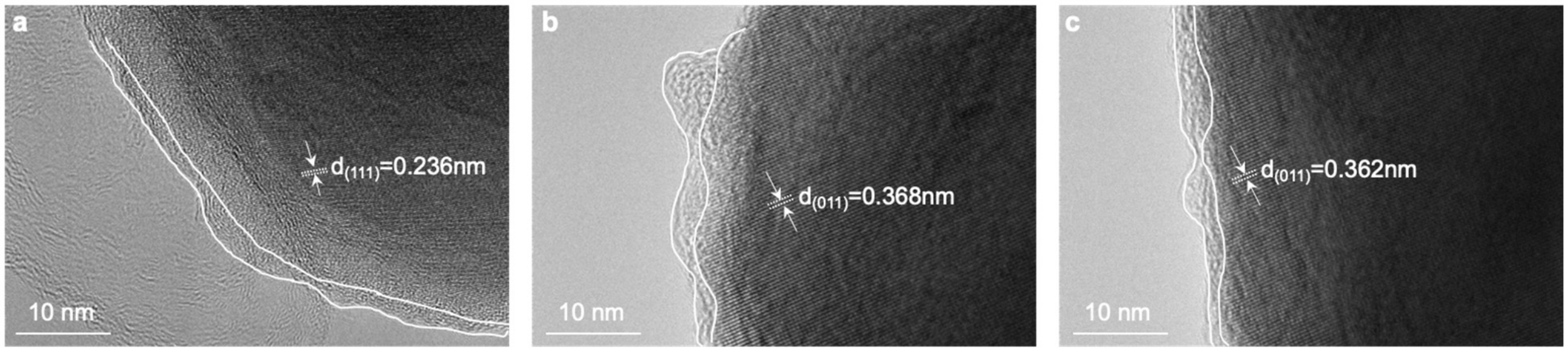

Supplementary Figure 28: High-resolution TEM of electrode interfaces. TEM image of (a) pLFP, (b) rLFP-PVDF, and (c) rLFP-PCC.

After recycling, the thickness of the carbon layer on the surface of rLFP-PCC particles are more uniform than rLFP-PVDF, indicating that the residual organic moieties were thermally decomposed under high-temperature conditions. The thermal decomposition produces pyrolytic carbon, which eventually deposits on the surface of rLFP-PCC particles, forming an amorphous carbon layer.

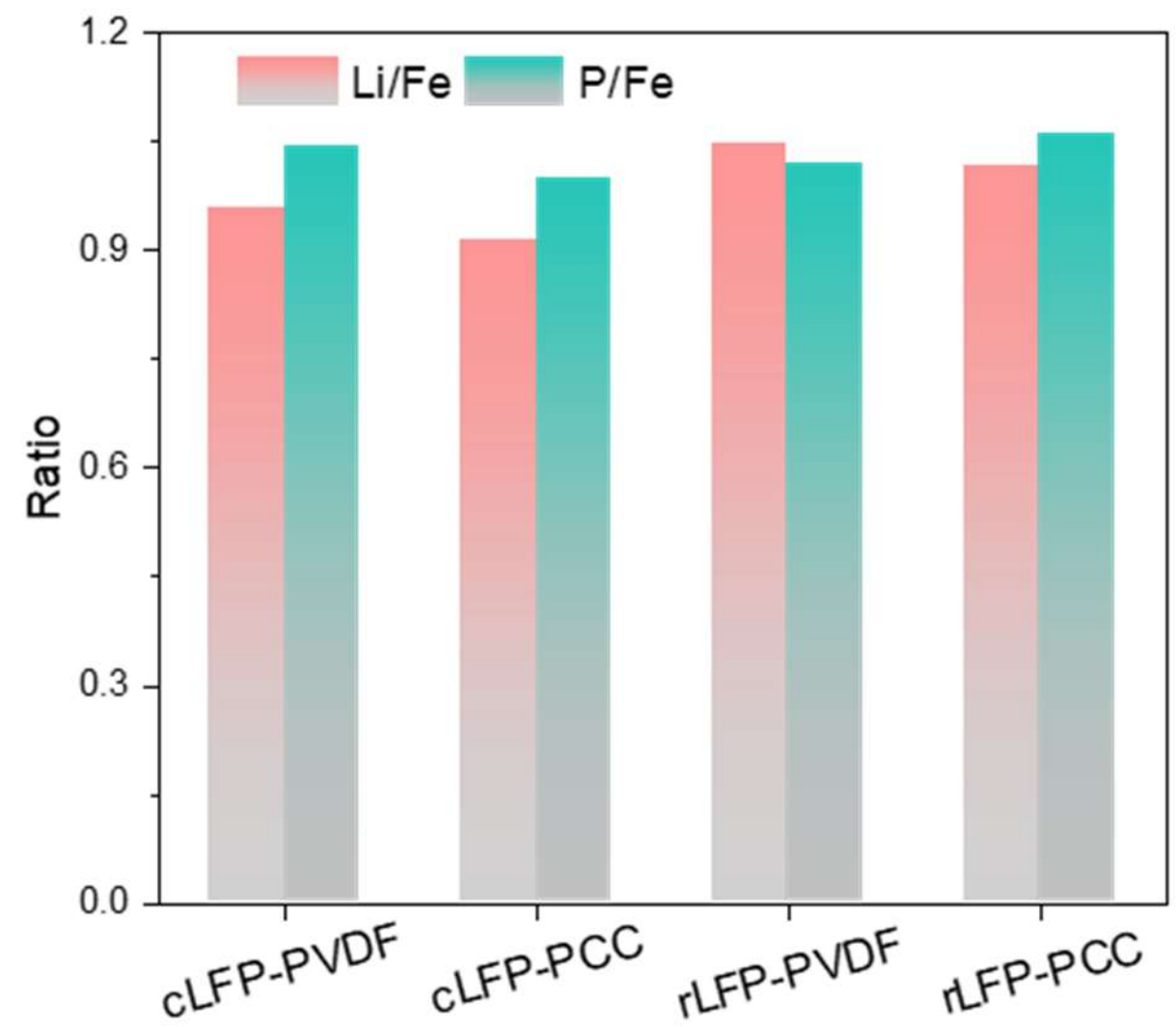

Supplementary Figure 29: Li/Fe ratio and P/Fe ratio of various LFP powder, calculated from ICP-MS tests.

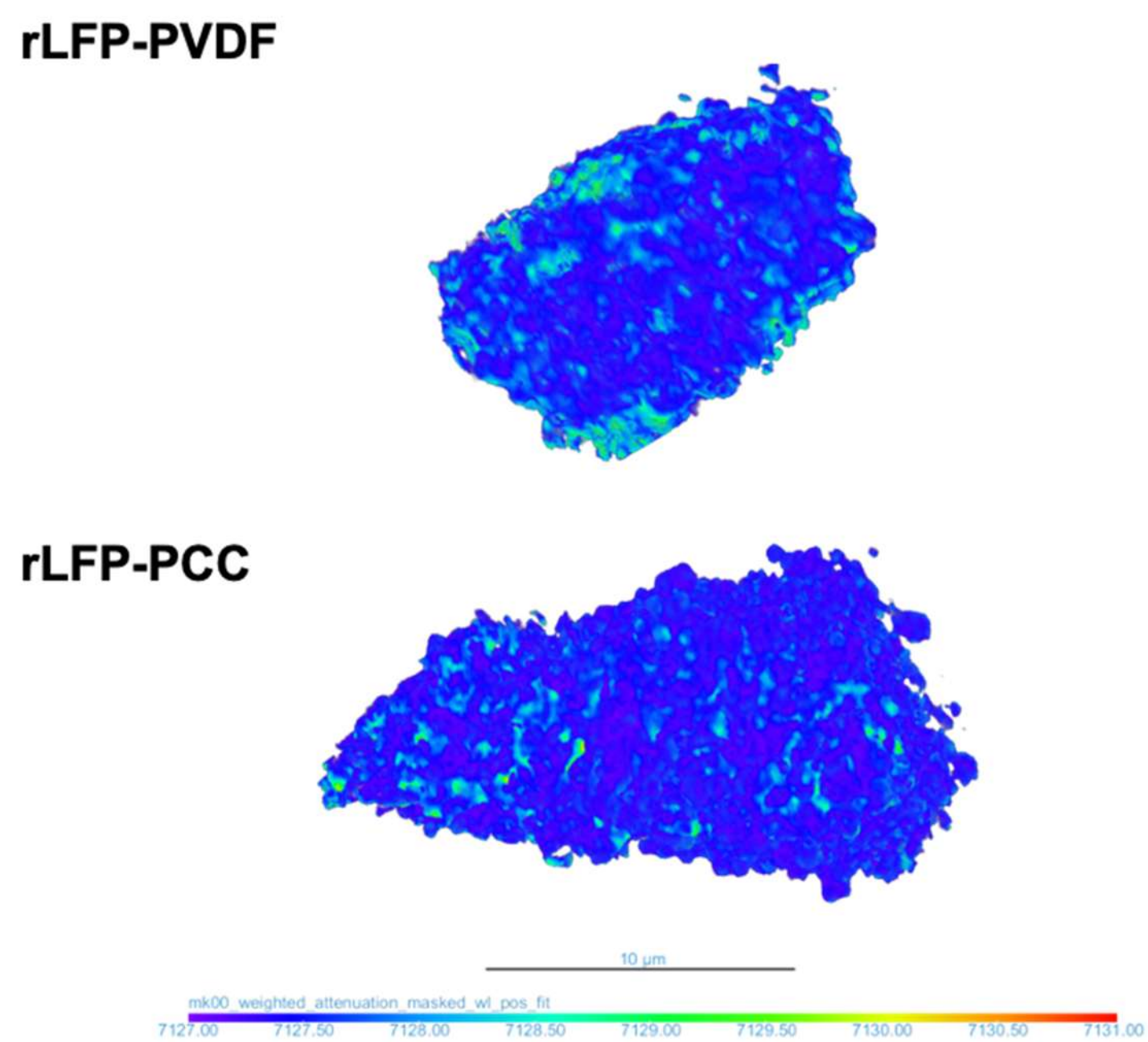

Supplementary Figure 30: **3D spectroscopic mapping of recycled LFP particles**, including rLFP-PVDF and rLFP-PCC.

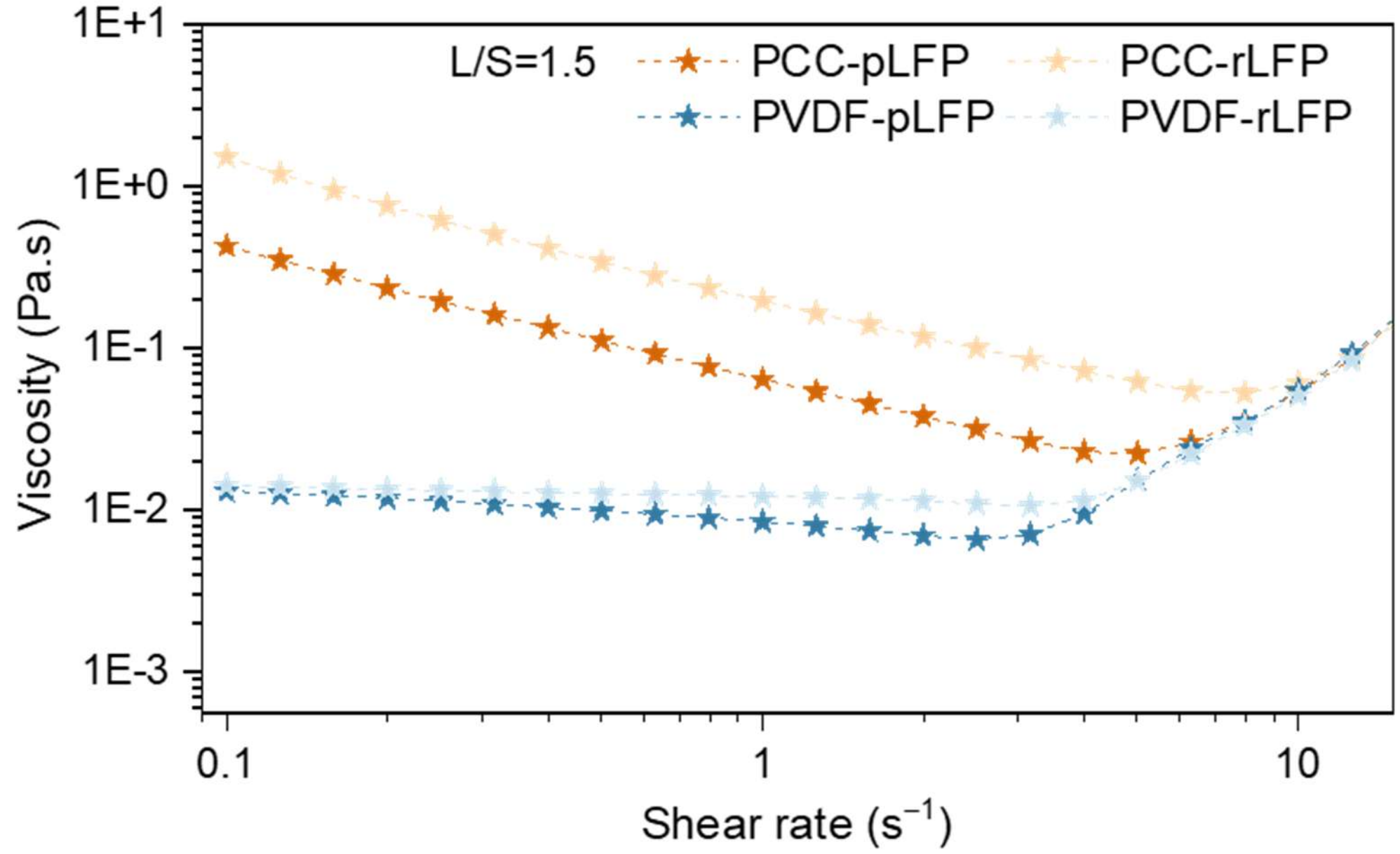

Supplementary Figure 31: **Time-dependent viscosity and dispersion behavior of LFP slurries.** Viscosity evolution of various LFP slurries prepared with PCC and PVDF binders as a function of holding time (1 h), measured under identical formulation conditions (liquid-to-solid ratio, L/S = 1.5; carbon additive omitted to isolate binder-LFP interactions) and identical rheological measurement settings.

Supplementary Figure 31 compares the shear-rate-dependent viscosity of slurries prepared using pristine (p) and recovered (r) LFP materials under otherwise identical formulation (L/S=1.5) and testing conditions. Measurements were conducted over the accessible shear-rate range of the rheometer to evaluate whether the recovery process alters slurry flow behavior and processability.

Across the measured shear-rate window, slurries prepared from recovered LFP exhibit viscosity profiles that closely track those of their pristine counterparts, indicating that the recovery process does not introduce significant changes in slurry rheology. Both pLFP and rLFP slurries show comparable shear-dependent behavior, with viscosity decreasing with increasing shear rate, consistent with shear-thinning flow typical of battery electrode slurries. Minor differences observed between pristine and recovered samples at low shear rates are attributed to subtle variations in particle surface condition or agglomeration state introduced during cycling and

recovery. However, these differences diminish at higher shear rates, which are more relevant to slurry mixing and coating processes. Importantly, the relative ordering of viscosity among different binder formulations is preserved for both pristine and recovered materials. Overall, the viscosity data indicate that the recovered materials from PCC system retain slurry processability comparable to pristine materials, supporting their compatibility with aqueous electrode fabrication processes.

Supplementary Tables

Supplementary Table 1: List of the investigated organic acids and their corresponding pK_a values in water at 25 °C.

Chemical	Chemical structure	pK_{a1}	pK_{a2}	pK_{a3}	pK_{a4}	Ref.
Oxalic acid	HOOC-COOH	1.25	4.14	—	—	15
Malonic acid	$HOOC-CH_2-COOH$	2.83	5.69	—	—	16
Succinic acid	$HOOC-(CH_2)_2-COOH$	4.2	5.6	—	—	16
Glutaric acid	$HOOC-(CH_2)_3-COOH$	4.32	5.42	—	—	16
Adipic acid	$HOOC-(CH_2)_4-COOH$	4.41	5.28	—	—	17
Suberic acid	$HOOC-(CH_2)_6-COOH$	4.526	5.498	—	—	18
Azelaic acid	$HOOC(CH_2)_7COOH$	4.550	5.498	—	—	18
Maleic acid	HOOC-CH=CH-COOH	1.9	6.07	—	—	19
Phthalic acid	$HOOC-C_6H_4-COOH$	2.89	5.51	—	—	20
2,5-Furandicarboxylic acid	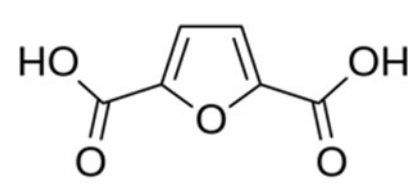	4.38	5.85	—	—	21
Lactic acid	$CH_3-CH(OH)COOH$	3.86	15.4	—	—	22,23
Tartaric acid	$HOOC-(CHOH)_2-COOH$	2.89	4.4	—	—	24
**Citric acid**	**$HOC(COOH)(CH_2COOH)_2$**	**3.13**	**4.76**	**6.39**	**14.4**	**22,25**
Glyceric acid	$HOCH_2CH(OH)COOH$	3.64	—	—	—	26
benzene-1,2,4-tricarboxylic acid	$C_6H_3(COOH)_3$	2.48	4.00	5.44	—	27
Aconitic acid	$HOOCCH_2C(COOH)=CHCOOH$	2.91	4.33	6.16	—	28

Boric acid	$B(OH)_3$	9.24	12.4	13.3	—	29
L-Ascorbic acid	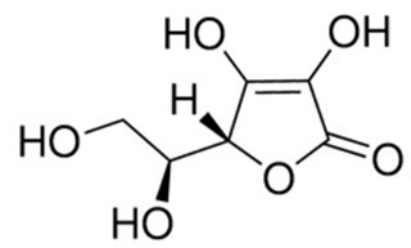	4.1	11.6	—	—	30,31
1,2,3,4-Butanetetracarboxylic acid	$HOOCCH_2CH(COOH)CH(COOH)CH_2COOH$	3.43	4.58	5.85	7.16	32
Gluconic acid	$HOCH_2(CHOH)_4COOH$	3.86	—	—	—	33
Ferulic acid	$(CH_3O)HOC_6H_3CH=CHCOOH$	4.61	—	—	—	34

Supplementary Table 2: Thermal analysis summary of various binders.

Sample	Low-temp Event (°C)	Primary decomposition (°C)	Secondary decomposition (°C)	High-temperature residual decomposition (°C)	Key implication
PAA	~100-150	~250-350	~380-500	—	Baseline polymer matrix with limited crosslinking.
PAA-CA	~150-200	~200-300	~350-500	—	CA-mediated esterification and increased crosslink density.
PAA-LiOH	~100-150	~200-350	~390-550	~550-750	Lithium carboxylate formation broadens thermal stability.
CMC	~80-150	~250-320	~400-500	~500-750	Non-crosslinked polysaccharide decomposition.
CMC-CA	~100-180	~230-320	~320-520	~620-750	Partial esterification with slightly earlier onset

CMC-PAA	~100-180	~250-420	~420-520	~620-750	Interpenetrating H-bonded polymer network.
CMC-PAA-LiOH	~100-200	~260-450	~460-520	~650-750	Lithium coordination improves thermal robustness.
PCC-2% CA	~180-300	~300-390	~390-520	~600-780	Under-crosslinked network.
PCC-5% CA	~160-300	~300-390	~390-520	~600-780	Optimal crosslinking window
PCC-8% CA	~180-300	~300-390	~390-520	~600-780	Optimal crosslinking window
PCC-10% CA	~160-300	~300-390	~390-520	~600-780	Over-crosslinking begins
PCC-20% CA	~160-250	~260-390	~390-520	~600-780	Over-crosslinked; diminishing thermal benefit

Low-temperature events correspond primarily to bound-water removal and initial thermally activated condensation reactions; primary and secondary decomposition regions are assigned based on the major mass-loss steps observed in TGA and the corresponding DSC thermal transitions.

Supplementary Notes

Supplementary Note 1

Theoretically assessment

Citric acid: $HOOC\text{-}CH_2\text{-}C(COOH)OH\text{-}CH_2\text{-}COOH$

Polyacrylic acid: $(CH_2\text{-}CHCOOH)_n$, Molecular weight of monomer is 72.

Carboxymethyl cellulose sodium: $C_6H_7O_2(OH)_{3-x}(OCH_2COONa)_x$, molecular weight of monomer is 242 when $x = 1$.

Assumption: DS is 1 for sodium substitution and 1 carboxymethyl groups per anhydroglucose unit.

$$R\text{-}COOH \rightleftharpoons R\text{-}COO^- + H^+ \quad (1)$$

$$pH = pK_a + \lg \frac{\text{proton acceptor}}{\text{proton donor}} \text{ (Henderson-Hasselbalch)} \quad (2)$$

$$H_3Cit \rightleftharpoons H_2Cit^- + H^+ \quad (3)$$

$$H_2Cit^- \rightleftharpoons HCit^{2-} + H^+ \quad (4)$$

$$HCit^{2-} \rightleftharpoons Cit^{3-} + H^+ \quad (5)$$

$$R\text{-}COOH \text{ (Citric acid)} + R'\text{-}OH \text{ (CMC)} \rightleftharpoons R\text{-}COO\text{-}R' + H_2O \quad (6)$$

$$R\text{-}OH \text{ (Citric acid)} + R''\text{-}COOH \text{ (PAA)} \rightleftharpoons R\text{-}COO\text{-}R'' + H_2O \quad (7)$$

$$R''\text{-}COOH\text{(PAA)} \cdots R'\text{-}OH \text{ (CMC) or } R'\text{-}OH \text{ (CMC)} \cdots R'\text{-}OH \text{ (CMC)} \quad (8)$$

$$R'\text{-}COO^- \cdots Li^+ \cdots R''\text{-}COO^- \quad (9)$$

Based on Eqs. 1 and 2, the dissociation of PAA and CMC at pH ≈6 is approximately 97% and 94%, respectively. Under these conditions, we assume a full sodium substitution for CMC (degree of substitution, DS = 1), corresponding to the chemical formula $C_6H_7O_2(OH)_2(OCH_2COONa)$ for CMCNa, with one carboxymethyl group per anhydroglucose unit. The esterification reaction, represented by Equations 6, 7, facilitates covalent grafting between the carboxylic groups of PAA or CMC and the hydroxyl groups of CA. This crosslinking mechanism underpins the structural

integrity of the binder network. The theoretical reaction extents under various CA additions are provided in **Supplementary Table 3**. For the PAA:CMC ratio of 1:1, the estimated grafting efficiency is summarized in **Supplementary Table 4**. Thermal analysis by DSC and TGA (**Supplementary Figure S2**) reveals the presence of excess unreacted CA when its content exceeds 10 wt.%, supporting our selection of 5-8 wt.% as the most suitable concentration. This optimized formulation ensures efficient crosslinking without compromising processability or thermal stability.

Supplementary Table 3: Theoretical reaction efficiency when the ratio of PAA to CMC is 1:1.

CA (wt.%)	CMC react with CA-COOH (%)	PAA react with CA-OH (%)
2	4.39	17.26
5	10.97	43.15
8	17.56	69.03
10	21.95	86.29
15	32.92	129.43

Supplementary Table 4: Theoretical reaction efficiency when adding 5% CA.

CMC	PAA	CMC react with CA-COOH (%)	PAA react with CA-OH (%)
0.5	0.5	10.97	43.15
1	0	5.48	—
0	1	—	21.57
0.3	0.5	18.29	30.81
0.75	0.25	7.31	86.29
0.8	0.2	6.85	107.86
0.25	0.75	21.95	28.76

Supplementary Table 5: Evolution of interfacial resistance components extracted from *in-situ* EIS analysis. The fitting quality is indicated by the normalized error ($\chi^2/|Z|^2$). The equivalent-circuit fitting was performed using the circuit shown in **Supplementary Fig. 16**, where the interfacial resistance is represented by R_{CEI}. In the present Li||LFP half-cell system, changes in R_{CEI} primarily reflect the evolution of the cathode electrolyte interphase (CEI), as the cathode binder is the only experimental variable.

Binder systems	States	R_s (Ω)	R_{CEI} (Ω)	R_{ct}(Ω)	χ^2 /$\|Z\|^2$
PVDF	Initial	2.0	15.14	247.9	0.03812
	1st cycle	1.996	20.39	152.3	0.03288
	2nd cycle	2.031	20.37	87.41	0.03215
	End of life (710 cycles)	2.464	6.647	45.01	0.0189
PCC	Initial	1.962	2.099	342.7	0.05341
	1st cycle	1.969	4.52	152.7	0.02448
	2nd cycle	2.164	3.992	30.43	0.03939
	End of life (1000 cycles)	4.927	3.378	3.656	0.02988

Supplementary Note 2

Life cycle assessment

In this section, we conducted in-depth study of life cycle assessments by using GREET 2024[35], BatPac 2024[36], and EverBatt 2023[37].

Environmental and processing comparison: PAA Vs. CMC Vs. PVDF

A comparative LCA of PVDF, PAA, and CMC reveals stark contrasts in their environmental burdens. PAA can be polymerized from bio-based acrylic acid, offering a scalable, water-processable binder compatible with negative electrode chemistries. Similarly, CMC, derived from cellulose, is inherently biodegradable and has been widely adopted in aqueous slurry processing for negative electrode systems. PVDF, the industry standard, is synthesized from vinylidene fluorinated monomers ($H_2C{=}CF_2$) through energy-intensive processes involving toxic solvents. The production of 1 kg PVDF emits approximately 55.8 kg CO_2-equivalent, consuming 90 MJ of energy[38]. This significantly undermines the environmental credibility of LIBs, especially as sustainable alternatives become increasingly viable. In contrast, PAA and CMC demonstrate orders-of-magnitude lower carbon footprints, with cradle-to-gate CO_2 emissions ranging between 1.5-2.7 kg CO_2-eq/kg, and energy requirements of just 32.2-38.7 MJ/kg.[38,39] Both polymers are synthesized via aqueous-phase or low-temperature reactions, often from renewable or bio-derived feedstocks. These environmental benefits, coupled with functional performance and compatibility with water-based electrode fabrication, support the consideration of PAA and CMC as promising alternatives for low-impact, recyclable LIB technologies. The drastic reduction in CO_2 emissions from binder production alone, over 90% lower than that of PVDF, underscores the critical importance of binder substitution in the broader strategy to decarbonize battery supply chains.

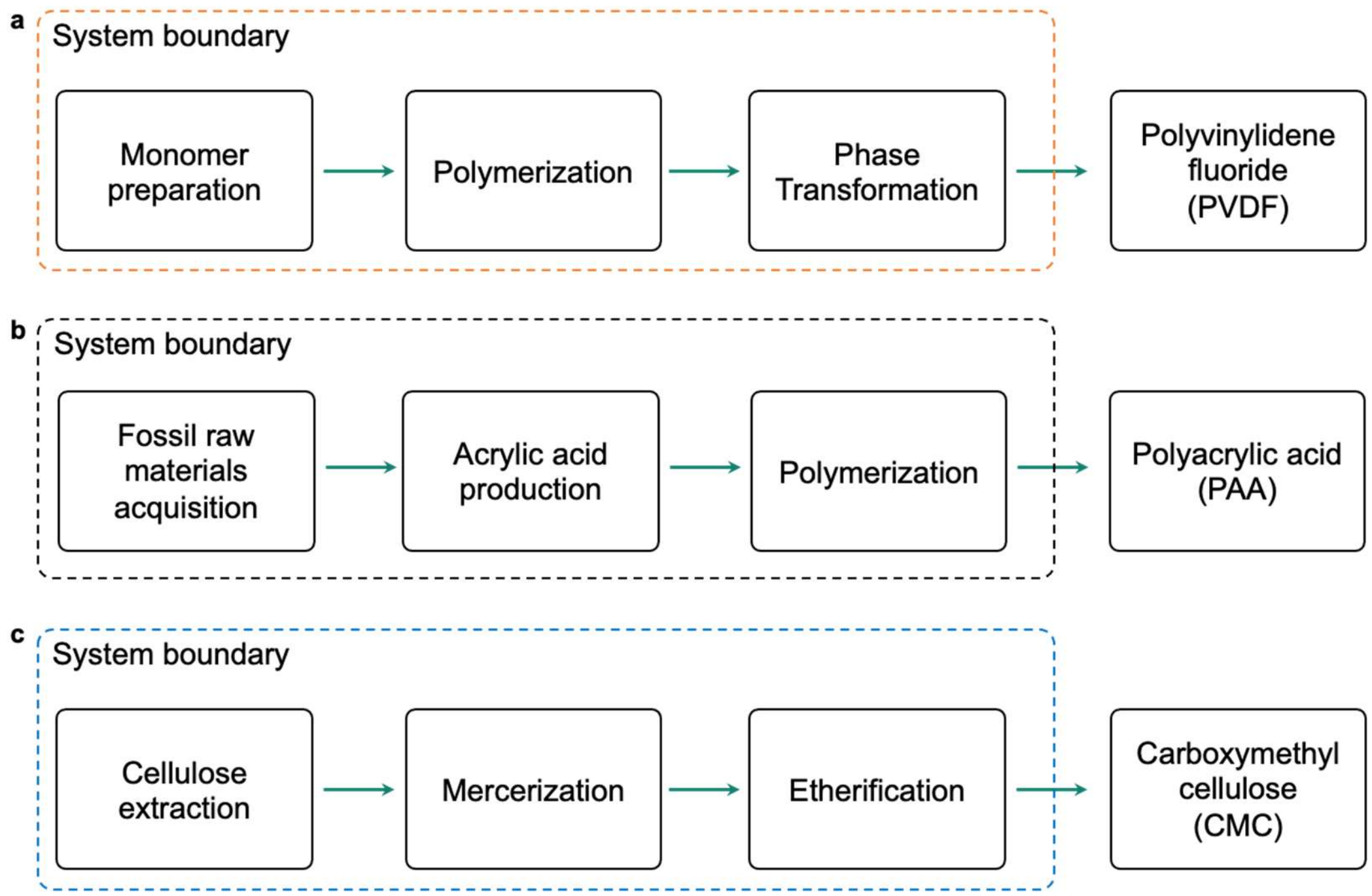

Supplementary Figure 32: Simplified description of **a.** PVDF, **b.** PAA, and **c.** CMC production system.

Environmental and processing comparison: NMP *vs*. Water

NMP has long served as the solvent of choice for PVDF-based electrode slurries in LIBs. However, its production, typically via high-temperature reaction of γ-butyrolactone and methylamine, requires substantial energy input (~98 MJ/kg) and emits significant greenhouse gases (~4.43kg CO_2-eq/kg), data source from GREET. Moreover, NMP is classified as a reproductive toxicant and a volatile organic compound (VOC), subject to increasingly stringent regulations across major markets[40,41]. In contrast, water is an inherently sustainable solvent with near-zero carbon emissions and energy requirements. Its compatibility with bio-derived, water-soluble binders has enabled a shift toward aqueous electrode fabrication. Overall, transitioning from NMP to water not only reduces environmental burdens across the cradle-to-gate life cycle, but also aligns electrode manufacturing with broader decarbonization and toxics-reduction goals. As battery manufacturing scales globally, the solvent system itself emerges as a critical and addressable determinant of sustainability.

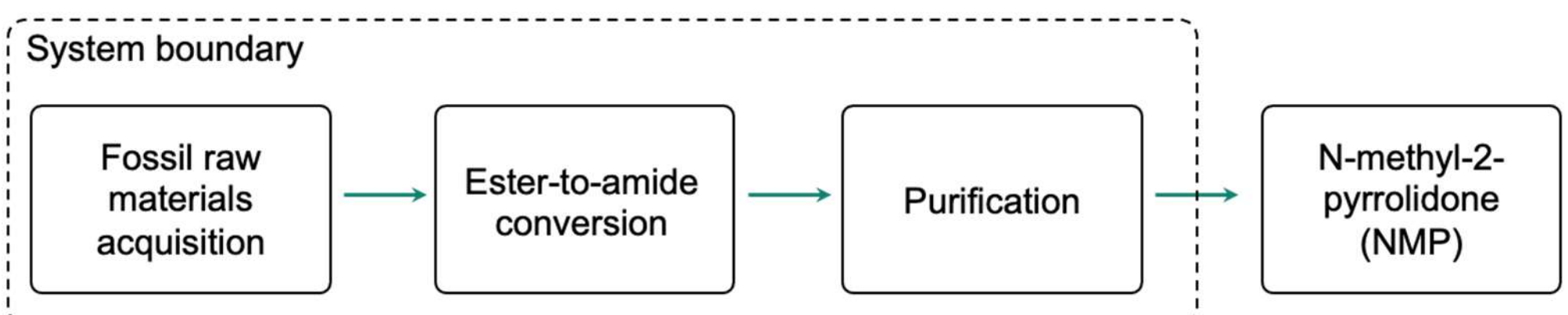

Supplementary Figure 33. Simplified description of NMP production system.

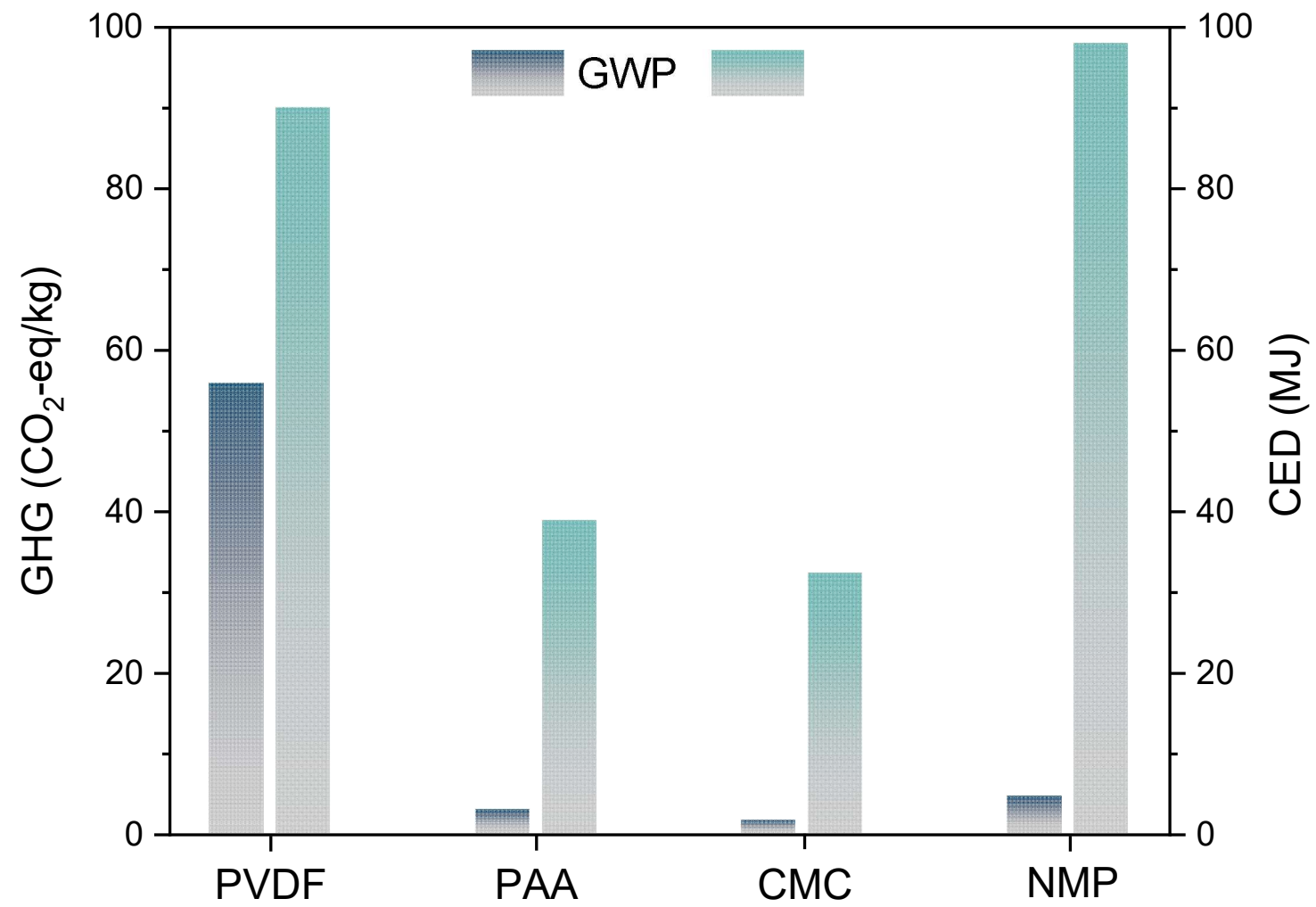

Supplementary Figure 34: Greenhouse gas (GHG) emission and cumulative energy demand (CED) comparison between PVDF, PAA, CMC, and NMP during the manufacture process.

LCA analysis of PVDF and PCC battery system

1. Goal and scope

The goal of the life cycle analysis is to consider and compare the potential economic and environmental effects in production and recycling LIBs. In particular, the greenhouse gas (GHG) emission, cumulative energy demand (CED), and water consumption are discussed in this section.

2. Scenario description, system boundaries, and inventory analysis

Two scenarios, PVDF cell manufacturing & recycling, PCC cell manufacturing & recycling process were considered in this part. In all the scenarios, manufacturing 0.25 kWh (based on Everbatt) of Gr||LFP LIBs and evaluate and normalize the material and energy flow according to the life cycle inventory, which is summarized in **Supplementary Tables 5-12**. The industrial production process of LIBs can be split into three stages: electrode manufacturing, cell fabrication, formation and integration, as shown in **Supplementary Figs. 35-36**.

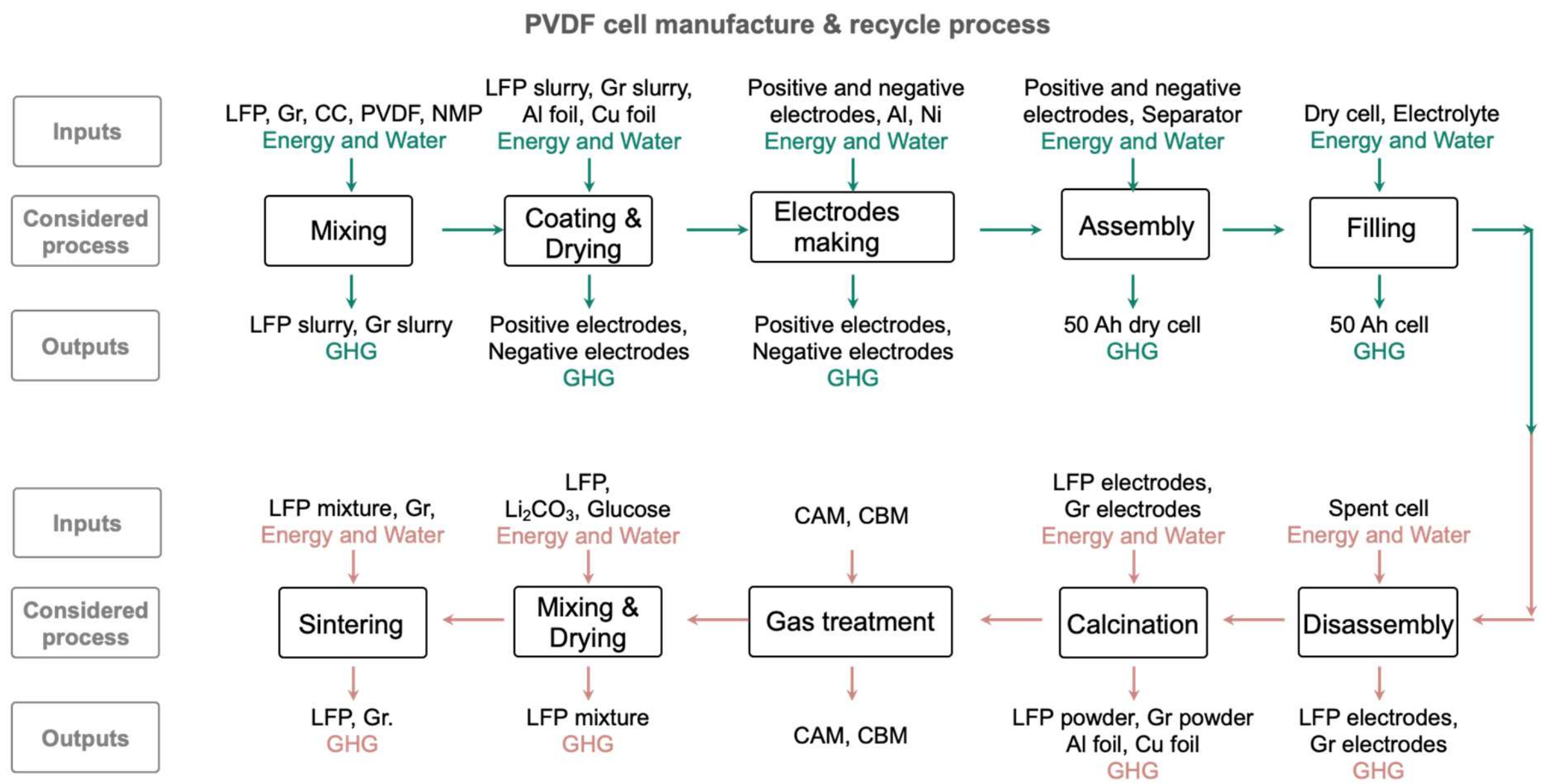

Supplementary Figure 35: Simplified description of PVDF manufacture and recycle system.

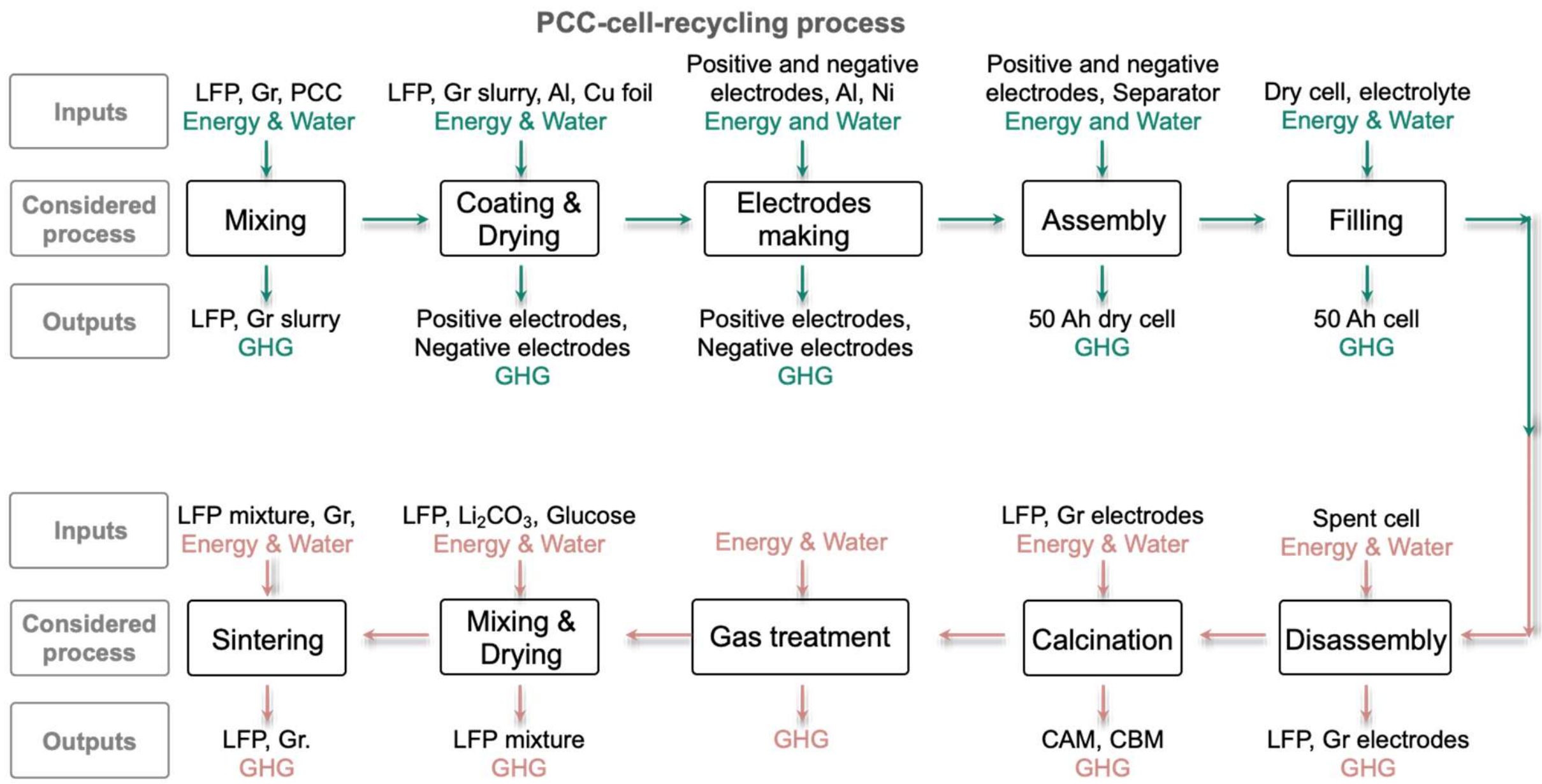

Supplementary Figure 36: Simplified description of PCC manufacture and recycle system.

Scenario 1 PVDF cell manufacture & recycle process

The fabrication of a 0.25 kWh LFP pouch cell follows a structured process consisting of electrode manufacturing, cell assembly, and formation & integration. The positive electrode is composited 96% LFP, 2% PVDF binder, and 2% carbon additive based on BactPac. The solvent/binder mass ratio is 16. The slurry is prepared with NMP as the solvent and coated onto a 15μm aluminum foil[42]. A representative NMP recovery efficiency of approximately 95% was assumed based on Everbatt. The negative electrode, formulated with 96% graphite, 2% carbon additive, and 2% negative electrode binder, prepared with H_2O as the solvent and solvent/binder mass ratio of 40, is coated onto 10μm copper foil with an N/P ratio of 1.1, ensuring slightly more negative electrode material to balance the Li^+ flow during cycling. The detailed lifecycle analysis for the production of a 0.25 kWh LFP pouch cell is provided in **Supplementary Tables 6-7**, encompassing material requirements, CED, water usage, GHG emissions,

Supplementary Table 6. Materials price for PVDF-NMP system, data source from BatPac.

Component	Material/Parameter	Price ($)
Positive Electrodes	LFP ($LiFePO_4$)	9.00/kg
	Binder (PVDF)	15.00/kg
	Conductive additive	7.00/kg
	Binder solvent (NMP)	2.70/kg
Negative Electrodes	Graphite	9.00/kg
	Binder	10.00/kg
	Conductive additive	7.00/kg
	Binder solvent (Water)	0.005/kg
Electrolyte	1.2 M $LiPF_6$ in EC/EMC	7.50/L
Separator	PE (Polyethylene)	0.90/m^2
Current Collectors	Aluminum Foil (positive electrode)	0.20/m^2
	Copper Foil (negative electrode)	1.20/m^2
Terminal	Positive terminal (Al)	2.41/kg
	Negative terminal (Cu)	8.64/kg
Cell Container	PET-Al-PP	3.00/kg
Heat conductor	Al	2.41/kg

Supplementary Table 7. Detail lifecycle analysis for the production of Gr||LFP by using PVDF-NMP system.

Material input		Quantity (kg/cell)	Cost ($/cell)
Electrodes	LFP ($LiFePO_4$)	0.521	4.69
	Graphite	0.257	2.32
	Conductive additive	0.016	0.11
	PVDF	0.011	0.16
	Binder (negative electrode)	0.005	0.05
Solvents and Electrolyte	$LiPF_6$	0.02	0.82
	EC	0.061	
	EMC	0.05	
	NMP	0.174	0.47
Separator	PE (Polyethylene)	0.003	1.61
Current Collectors	Aluminum foil	0.072	0.36
	Copper foil	0.117	1.57
Others	PET	0.003	0.05
	PP	0.011	
	Cell container	$0.2/cell	
	Heat conductor	$0.15/cell	
Terminal	Positive terminal	$0.08/cell	
	Negative terminal	$0.08/cell	
Energy & Process Consumption	Cell Assembly & Formation		$5.63/cell
**Total energy consumption**			**0.173 mmBtu/cell**
**Total water consumption**			**11 gal/cell**
**Total GHG emissions**			**12.81 kg/cell**
**Total Cost**			**$18.35/cell**

The solid-state relithiation process for a LFP cell begins with the discharge and disassembly of the battery. The battery is safely discharged to eliminate any stored energy, reducing the risk of accidental short circuits during disassembly. Afterward, mechanical methods are used to separate the various components, including the current collectors and separators. The positive and negative electrodes are separated, and the polyethylene separator is extracted for later disposal. Following electrode separation, the next step involves cleaning the materials using ethyl methyl carbonate (EMC) to remove any residual electrolyte or surface films prior to binder removal[43]. The LFP positive electrode is then subjected to NMP to dissolve and remove the PVDF binder[44]. This cleaning process ensures that the LFP material is free of organic residues, which is essential for the subsequent lithium supplementation. The cleaned LFP powder is mixed with Li_2CO_3 to compensate for about 20% lithium deficiency. The mixture is heated in an argon atmosphere at 650°C for 10 hours to restore the lithium content and improve the performance of the material[45]. Simultaneously, the graphite material is regenerated by high-temperature annealing in an argon atmosphere at 1000°C for 4 hours to heal structural disorder, drive off organics, and improve conductivity/rate capability[46]. Throughout, off-gas management is essential (e.g., HF mitigation when processing PVDF-bearing materials at elevated temperature)[47].

Supplementary Table 8. Detail cost analysis for the recycling of Gr||LFP for PVDF-NMP system.

Process Step & materials		Quantity (/kg spent-cell feed)	Price ($/unit)	Cost ($/kg spent-cell feed)
Preprocessing	Nitrogen	0.05 kg	0.3/kg	0.015
	Lime	0.04 kg	0.13/kg	0.0052
	Diesel	0.6 MJ	Data unavailable	Not included
	Natural gas	2 MJ	7.77/mmBtu	0.01473
	Electricity	0.2 MJ	0.08/ kWh	0.00444
	Process water	0.1 gal	0.005/gal	0.0005
	Flue Dust	0.00097 kg	54.03/ton	0.01007
	Waste (solid)	0.18554 kg		
	Waste (water)	0.37854 kg	0.006/gal	0.00060
**Preprocessing subtotal**	**0.05055**			
CM recovery and regeneration[a]	Lithium Carbonate	0.01776 kg	17.14/kg	0.30433
	NMP circulated	0.15195 kg	$2.70/kg	0.41027
	NMP recovered	0.14435 kg	95% recovery	0.00434
	Sulfuric Acid (for Gr cleaning)	0.14963 kg	0.08/kg	0.01197
	Diesel	0.3990 MJ	Data unavailable	Not included
	Natural gas	1.123885 MJ	7.77/mmBtu	0.00828
	Electricity	6.57685 MJ	0.08/ kWh	0.14615
	Process water	0.6650 gal	0.005/gal	0.00333
	Waste (solid)	0.08645 kg	54.03/ton	0.00467
	Waste (water)	2.53365 kg	0.006/gal	0.00402
**CM-recovery subtotal**	**0.89736**			
**Total Cost**			**$0.94791**	

[a] The CM-recovery inventory was originally calculated per kilogram of black mass and was multiplied by the PVDF-scenario black-mass yield of 0.665 kg black mass per kilogram of spent-cell feed.

[b] The total excludes diesel, capital, labor, maintenance, and plant-overhead costs because these values were not available or were not included in the custom process model.

Supplementary Table 9. Detail lifecycle analysis for the recycling of Gr||LFP for PVDF-NMP system

Process Step	CED (mmBtu/spent-cell feed)	Water Consumption (gal/kg spent-cell feed)	GHG emissions (kg CO_2-eq./kg spent-cell feed)	Listed input cost ($/kg spent-cell feed)	Revenue ($/kg spent-cell feed)
Preprocessing	0.00337	0.16474	0.49355	0.05055	0.67794
CM recovery and regeneration[a]	0.01883	2.02202	1.30839	0.50325	5.26680
**Total energy consumption**	**0.02220 mmBtu/spent-cell feed**				
**Total water consumption**	**2.18676 gal/kg spent-cell feed**				
**Total GHG emissions**	**1.80194 kg CO_2-eq./kg spent-cell feed**				
**Total cost**	**$0.94791/kg spent-cell feed**				
**Total revenue**	**$5.94474 kg spent-cell feed**				
**Total Profit[b]**	**$4.99683 kg spent-cell feed**				

[a] CM-recovery results were multiplied by 0.665 kg black mass per kilogram of spent-cell feed. The environmental burden associated with NMP production was calculated using the 5% fresh NMP makeup required under the assumed 95% NMP recovery efficiency. The energy demand of the modeled recycling process was retained.

[b] Profit was calculated as recovered-material revenue minus the listed process-input cost. It should not be interpreted as complete industrial profit because diesel, capital, labor, maintenance, and overhead costs were excluded.

Revenue calculation:

$R_{PVDF} = R_{preprocessing} + R_{CM} = 0.05923_{Al} + 0.61871_{Cu} + (0.59 \times \$9 \times 0.665)_{LFP} + (0.29 \times \$9 \times 0.665)_{Gr}$

$R_{PVDF} = \$5.94474$/kg spent feed

Scenario 2 PCC cell manufacture & recycle process

The fabrication of a 0.25 kWh LFP pouch cell follows a structured process consisting of electrode manufacturing, cell assembly, and formation & integration. The positive electrode is composited 97% LFP, 2% PCC binder, and 1% carbon additive. The solvent/binder mass ratio is 40. The slurry is prepared with water as the solvent and coated onto a 15μm aluminum foil[42]. The negative electrode, formulated with 96% graphite, 2% carbon additive, and 2% PCC binder, prepared with H_2O as the solvent and solvent/binder mass ratio of 40, is coated onto 10μm copper foil with an N/P ratio of 1.1, ensuring slightly more negative electrode material to balance the Li^+ flow during cycling. The detailed lifecycle analysis for the production of a 0.25 kWh LFP pouch cell is provided in **Supplementary Tables 10-11**, encompassing material requirements, CED, water usage, GHG emissions, and associated costs.

Supplementary Table 10. Materials price for PCC-H_2O system, data source from BatPac.

Component	Material/Parameter	Price ($)
Positive Electrodes	LFP ($LiFePO_4$)	9.00/kg
	Binder	3.00/kg
	Conductive additive	7.00/kg
	Binder solvent (Water)	0.005/kg
Negative Electrodes	Graphite	9.00/kg
	Binder	3.00/kg
	Conductive additive	7.00/kg
	Binder solvent (Water)	0.005/kg
Electrolyte	1.2 M $LiPF_6$ in EC/EMC	7.50/L
Separator	PE (Polyethylene)	0.90/m^2
Current Collectors	Aluminum Foil (positive electrode)	0.20/m^2
	Copper Foil (negative electrode)	1.20/m^2
Terminal	Positive terminal (Al)	2.41/kg
	Negative terminal (Cu)	8.64/kg
Cell Container	PET-Al-PP	3.00/kg
Heat conductor	Al	2.41/kg

Supplementary Table 11. Detail lifecycle analysis for the production of Gr||LFP by using PCC-H_2O system.

Material input		Quantity (kg/cell)	Cost ($/cell)
Electrodes	LFP ($LiFePO_4$)	0.521	4.69
	Graphite	0.257	2.32
	Conductive additive	0.011	0.08
	PCC	0.016	0.05
Solvents and Electrolyte	$LiPF_6$	0.02	0.82
	EC	0.061	
	EMC	0.05	
	NMP	0	0
Separator	PE (Polyethylene)	0.003	1.61
Current Collectors	Aluminum foil	0.072	0.36
	Copper foil	0.117	1.57
Others	PET	0.003	0.05
	PP	0.011	
	Cell container	$0.2/cell	
	Heat conductor	$0.15/cell	
Terminal	Positive terminal	$0.08/cell	
	Negative terminal	$0.08/cell	
Energy & Process Consumption	Cell Assembly & Formation		$5.63/cell
**Total energy consumption**			**0.157 mmBtu/cell**
**Total water consumption**			**10.8 gal/cell**
**Total GHG emissions**			**11.924 kg/cell**
**Total Cost**			**$17.69/cell**

The recycling of a LFP pouch cell fabricated with a water-processable PCC binder system, follows a streamlined and environmentally favorable approach enabled by the intrinsic water solubility and thermal degradability of the binder. The spent cell is first fully discharged to mitigate electrical hazards, followed by mechanical disassembly to retrieve the positive electrode and negative electrode laminates, separator, and metallic current collectors. Unlike PVDF-based systems that require organic solvents such as NMP for binder removal, the PCC binder network dissolves efficiently in water, eliminating the need for toxic solvents. The electrodes are immersed in water under moderate agitation and temperature (~50 °C), enabling clean delamination of the active materials from both the aluminum and copper foils. Residual binder and citric-acid-based crosslinks are decomposed or hydrolyzed during the process, minimizing chemical complexity in the recovered solids. The extracted LFP powder is filtered, rinsed, and directly mixed with a stoichiometric amount of Li_2CO_3 to compensate for lithium loss during battery operation. This mixture is subjected to a solid-state relithiation step at 650 °C under an argon atmosphere for 10 hours, regenerating the electrochemical performance of the LFP material[45]. Simultaneously, the graphite material is regenerated by high-temperature annealing in an argon atmosphere at 1000°C for 4 hours to heal structural disorder, drive off organics, and improve conductivity/rate capability[46]. Importantly, the absence of fluorinated polymers and halogenated solvents significantly reduces GHG emissions and wastewater treatment burden. All process steps are designed to minimize waste generation and energy consumption.

Supplementary Table 12. Detail cost analysis for the recycling of Gr||LFP for PCC-based system.

Process Step & materials		Quantity (/kg spent-cell feed)	Price ($/unit)	Cost ($/kg spent-cell feed)
Preprocessing	Nitrogen	0.05 kg	0.3/kg	0.015
	Lime	0.04 kg	0.13/kg	0.0052
	Diesel	0.6 MJ	Data unavailable	Not included
	Natural gas	2 MJ	7.77/mmBtu	0.014729858
	Electricity	0.2 MJ	0.08/ kWh	0.00444
	Process water	0.00097 gal	0.005/gal	0.00050
	Flue Dust	0.00097	54.03/ton	0.01007
	Waste (solid)	0.18554		
	Waste (water)	0.37854	0.006/gal	0.00060
Preprocessing subtotal	0.05055			
CM recovery and regeneration[a]	Lithium Carbonate	0.01749 kg	17.14/kg	0.29975
	Diesel	0.3930 MJ	Data unavailable	Not included
	Natural gas	1.10695 MJ	7.77/mmBtu	0.00815
	Electricity	6.47795 MJ	0.08/ kWh	0.14395
	Process water	0.6550 gal	0.005/gal	0.00328
	Waste (solid)	0.08515 kg	54.03/ton	0.00460
	Waste (water)	2.49555 kg	0.006/gal	0.00396
**CM-recovery subtotal**	**0.46369**			
**Total Cost[b]**			**$0.51424**	

[a] The CM-recovery inventory was originally calculated per kilogram of black mass and was multiplied by the PCC-scenario black-mass yield of 0.655 kg black mass per kilogram of spent-cell feed.

[b] The total excludes diesel, capital, labor, maintenance, and plant-overhead costs.

Supplementary Table 13. Detail lifecycle analysis for the recycling of Gr||LFP for PCC-based system.

Process Step	CED (mmBtu/spent-cell feed)	Water Consumption (gal/kg spent-cell feed)	GHG emissions (kg CO_2-eq./kg spent-cell feed)	Listed input cost ($/kg spent-cell feed)	Revenue ($/kg spent-cell feed)
Preprocessing	0.00337	0.16474	0.49355	0.05055	0.67794
Recycling process	0.01656	1.95845	1.17869	0.46369	5.18760
**Total energy consumption**		**0.019993 mmBtu/spent-cell feed**			
**Total water consumption**		**2.12318 gal/kg spent-cell feed**			
**Total GHG emissions**		**1.67224 kg CO_2-eq./kg spent-cell feed**			
**Total cost**		**$0.51424/kg spent-cell feed**			
**Total revenue**		**$5.86554/kg spent-cell feed**			
**Total profit**		**$5.35130/kg spent-cell feed**			

[a] CM-recovery results were multiplied by 0.655 kg black mass per kilogram of spent-cell feed.

[b] Profit was was calculated as recovered-material revenue minus the listed process-input cost. It should not be interpreted as complete industrial profit because diesel, capital, labor, maintenance, and overhead costs were excluded.

Revenue calculation:

$R_{PVDF} = R_{preprocessing} + R_{CM} = 0.05923_{Al} + 0.61871_{Cu} + (0.59 \times \$9 \times 0.655)_{LFP} + (0.29 \times \$9 \times 0.665)_{Gr}$

$R_{PVDF} = \$5.86554$/kg spent feed

Supplementary Note 3

In this section, we perform an in-depth fluorine mass-balance and NMP impact assessment for both PVDF - and PCC-based cell systems, the system boundary is defined in **Supplementary Fig. 30**.

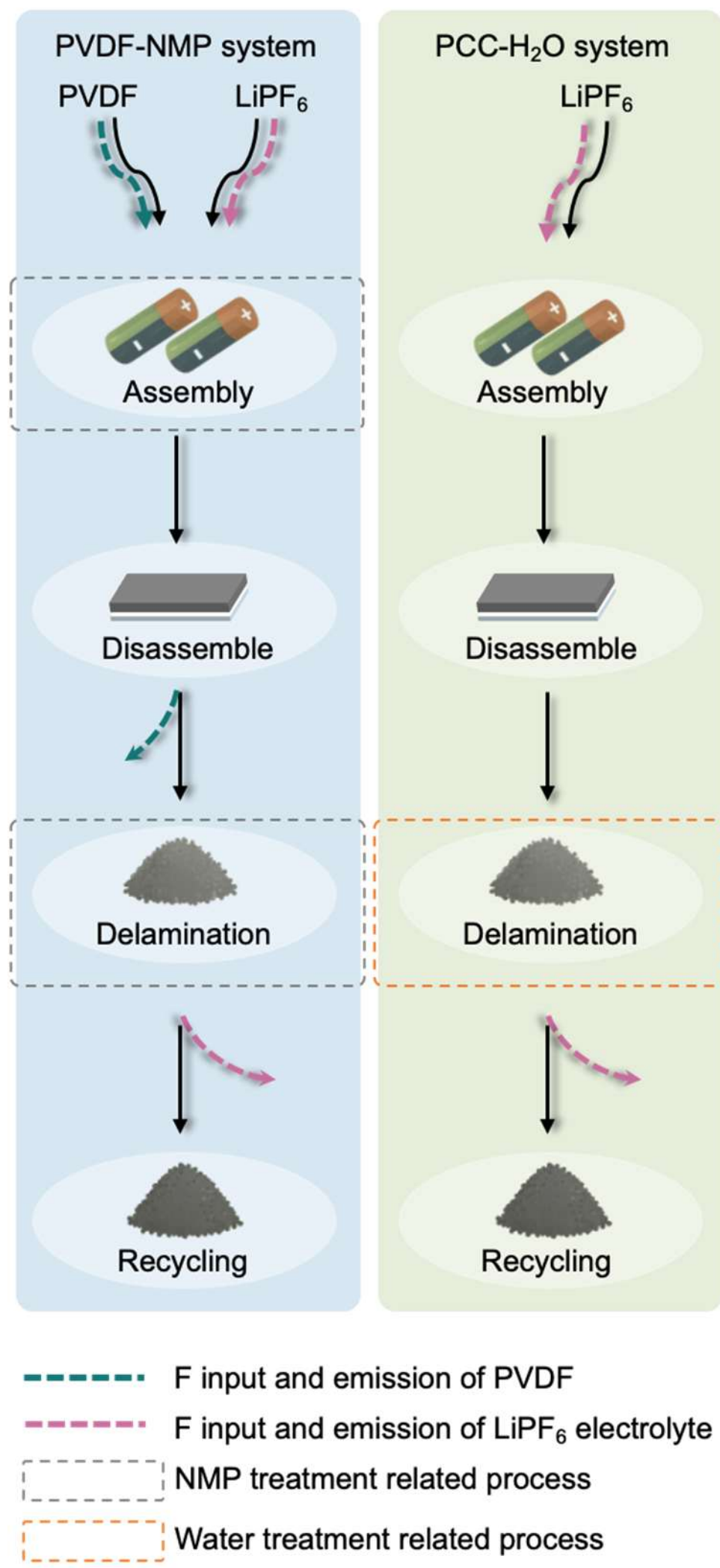

Supplementary Figure 37: The environmental impacts of fluorine input and emission of the PCC-H_2O battery system and PVDF-NMP battery system, as well as the influence of solvents (NMP treatment related process).

Fluorine input and emission analysis

Scenario 1 PVDF cell manufacture & recycle process

During the fabrication of a 0.25 kWh Gr||LFP cell, fluorine is introduced primarily through two sources: the PVDF binder and the $LiPF_6$ electrolyte salt. Specifically, PVDF contributes approximately 6.53 g of fluorine per cell, while $LiPF_6$ accounts for an additional 14.99 g/cell, based on the mass input and elemental fluorine content. During the manufacturing process, we assume that there is no fluorine emission, so the main emission concern will come from the subsequent recycling steps.

During positive electrode pretreatment of recycling, NMP-based solvent extraction is typically employed to recover and purify active materials. However, only about 80% of the PVDF binder can be effectively removed from the electrode surface, corresponding to ~5.22 g/cell of fluorine removed as dissolved PVDF.[48] The remaining ~20% (1.3 g/cell of fluorine) is often retained in the porous electrode matrix due to physical embedding or chemical adhesion to active material surfaces, after drying. In subsequent high-temperature treatments (during relithiation or calcination), the residual PVDF undergoes thermal decomposition, forming a mixture of volatile fluorinated species such as HF[49]. If not effectively scrubbed by off-gas systems, these species are released into the environment. It is estimated that up to 99% of the unrecovered fluorine from PVDF may be volatilized under such conditions. Only trace amounts (~0.007 g/cell) may react with lithium or transition metals to form stable lithium fluoride (LiF) residues on the material surface[48].

In contrast, $LiPF_6$, which contains six fluorine atoms per molecule (74.97 wt% fluorine), is intrinsically unstable and undergoes thermal and hydrolytic decomposition even under moderate cycling conditions. Approximately 60% of the fluorine from $LiPF_6$ (~8.99 g/cell) is converted into LiF, which forms electrically insulating deposits on negative electrode SEI layers and positive electrode interfaces[8,50]. The remaining ~6.0 g/cell of fluorine is typically released as a mixture of PF_5, POF_3, and HF[51]. These volatile and corrosive species typically remain sealed within the cell until end-of-life processing but present significant safety and emissions concerns upon cell disassembly. During recycling, LiF is thermodynamically very stable and largely persists as a solid on active material/graphite surfaces. Only a minor portion of fluorine may enter aqueous streams

(LiF has very low water solubility, (~0.09g/cell), unless strong acids or specific fluorine-capturing reagents are used.

Scenario 2 PCC cell manufacture & recycle process

In cells employing PCC binder systems, fluorine input is limited exclusively to $LiPF_6$ electrolyte, contributing the same 14.99 g/cell of fluorine. Similarly, approximately 60% of the fluorine from $LiPF_6$ (~8.99 g/cell) is converted into LiF, which forms electrically insulating deposits on negative electrode SEI layers and positive electrode interfaces[8,50]. The remaining ~6.0 g/cell of fluorine is typically released as a mixture of PF_5, POF_3, and HF[51], because $LiPF_6$ is known to be thermally and hydrolytically unstable, even at moderate temperatures and under trace moisture. These volatile and corrosive species typically remain sealed within the cell until end-of-life processing but present significant safety and emissions concerns upon cell disassembly. During recycling, LiF is thermodynamically very stable and largely persists as a solid on active material/graphite surfaces. Only a minor portion of fluorine may enter aqueous streams (LiF has very low water solubility, ~0.09g/cell), unless strong acids or specific fluorine-capturing reagents are used.

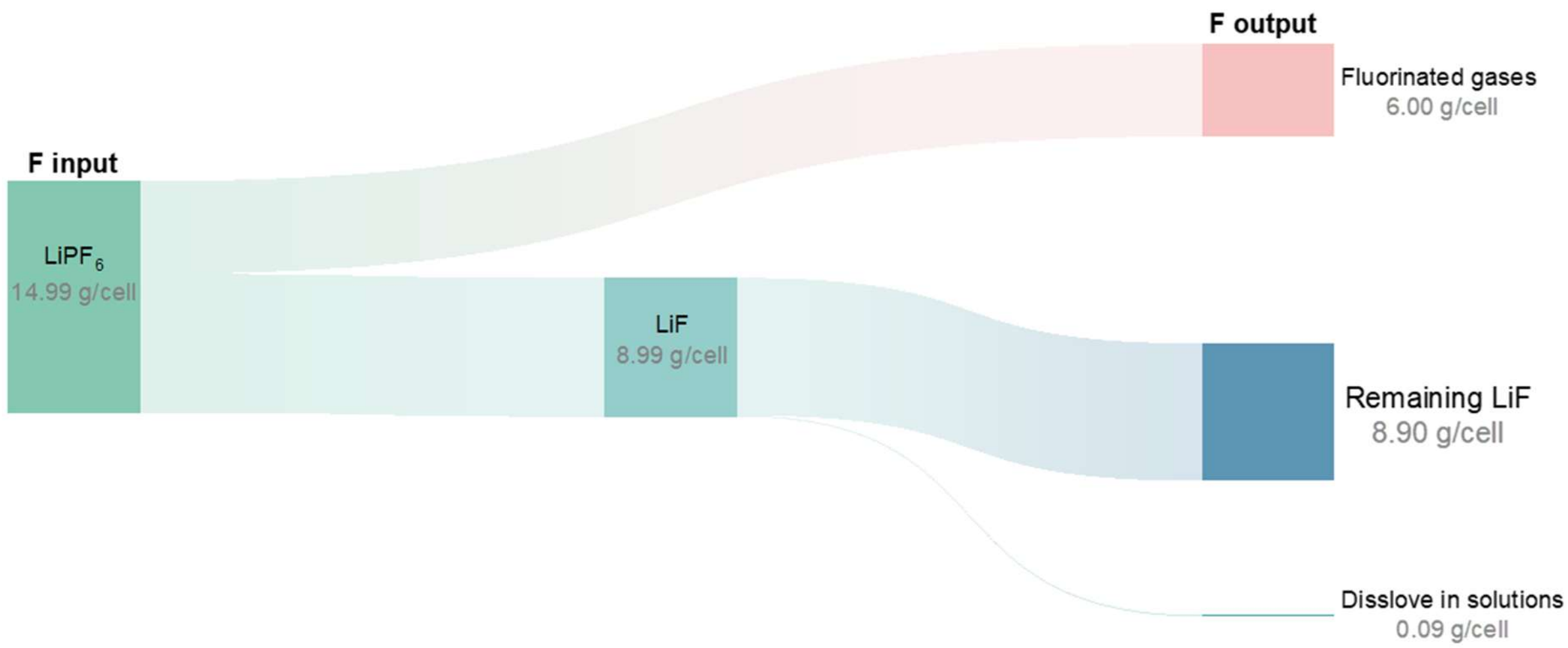

Supplementary Figure 38. Fluorine input and output analysis during the manufacture and recycle process of PCC-based system.

NMP input and emission analysis

Scenario 1. NMP input and emission during PVDF cell manufacture process

In the fabrication of a 0.25 kWh Gr||LFP battery, approximately 174 g/cell of NMP is consumed per cell as a dispersing solvent for the PVDF binder. Based on industrial-scale recovery systems, a solvent recovery efficiency of ~95% is commonly achievable via vacuum-assisted distillation or condensation units[36,52]. Nevertheless, approximately 8.7 g/cell of NMP is unavoidably lost, primarily through the following pathways: (1) Incomplete solvent evaporation or capture (~3.0 g/cell): due to residual vapor loss during electrode drying stages, especially in older or poorly sealed systems[52]. (2) Entrapment within dried electrode layers (~2.5 g/cell): a fraction of NMP remains physically absorbed or capillary-retained within the porous electrode architecture[53]. (3) Surface adhesion on slurry mixing and coating equipment (~1.5 g/cell): mechanical cleaning cannot fully remove all solvent residues from reactor walls or pumps[52]. (4) Contamination in waste slurry (~1.7 g/cell): particularly prevalent in the disposal of off- specification batches or cleaning cycles. Despite this relatively high recovery rate, the environmental burden of NMP loss remains significant. NMP is currently restricted under Annex XVII of the Registration, Evaluation, Authorization and Restriction of Chemicals (REACH) under the European Chemicals Agency (ECHA)[54] and is a notable contributor to VOC emissions, necessitating robust containment, exhaust gas scrubbing, and post-use solvent purification strategies for large-scale production.

Scenario 2. NMP input and emission during PVDF cell recycling process

In the recycling phase, NMP is also widely employed for PVDF binder extraction, where approximately 230 g of NMP is required per kilogram of feedstock to ensure sufficient solubilization and diffusion of the fluorinated binder from composite electrodes[55]. Assuming a similar 95% recovery rate via vacuum distillation, about 218.5 g of NMP can be reclaimed, with ~11.5 g lost through: volatilization during high-temperature leaching or filtration (~5 g/kg feed processed), adsorption onto carbonaceous or polymeric residues (~3-4 g/kg feed processed), and incorporation into wash effluents with high chemical oxygen demand (~2.5-3.0 g/kg feed processed)[52,53]. While this NMP-based removal process facilitates efficient fluorine decoupling from active materials and enables cleaner downstream separation, it generates secondary waste streams, including NMP-PVDF complexes, fluorinated decomposition byproducts, and high-COD aqueous filtrates, all of which demand strict handling protocols due to NMP's toxicity and persistence in aquatic systems[56].

Supplementary References

1 Zhou, J., Wu, S., Li, Q. & Wu, F. Biologically Insoluble Binder for High-Performance Cathodes in Lithium-Ion Batteries. *Adv. Energy Mater.* **15**, 2406032, (2025).

2 Özen, İ., Okyay, G. & Ulaş, A. Coating of nonwovens with potassium nitrate containing carboxymethyl cellulose for efficient water and fertilizer management. *Cellulose* **25**, 1527-1538, (2018).

3 He, Y., Zhu, B. & Inoue, Y. Hydrogen bonds in polymer blends. *Prog. Polym. Sci.* **29**, 1021-1051, (2004).

4 Han, M., Liang, X., Han, C., Zhou, C., Xiang, Q., Cao, Y. & Sun, J. Cross-Linked Binary Polymeric Binder of Polyacrylic Acid–Carboxymethyl Cellulose for Improving Phosphorus Anode. *Adv. Funct. Mater.* **34**, 2308325, (2024).

5 Palanivel, T., Mamlouk, M., Pollet, B. G. & Vinodh, R. Exploring properties of hyperbranched polymers in anion exchange membranes for fuel cells and its potential integration for water electrolysis: A review. *J. Energy Chem.* **102**, 670-702, (2025).

6 Lu, Y., Zhao, C.-Z., Huang, J.-Q. & Zhang, Q. The timescale identification decoupling complicated kinetic processes in lithium batteries. *Joule* **6**, 1172-1198, (2022).

7 Li, C.-C., Chen, C.-A. & Chen, M.-F. Gelation mechanism of organic additives with $LiFePO_4$ in the water-based cathode slurries. *Ceram. Int.* **43**, S765-S770, (2017).

8 Xu, K. Electrolytes and Interphases in Li-Ion Batteries and Beyond. *Chem. Rev.* **114**, 11503-11618, (2014).

9 Huang, J., Liu, J., He, J., Wu, M., Qi, S., Wang, H., Li, F. & Ma, J. Optimizing electrode/electrolyte interphases and Li-ion flux/solvation for lithium-metal batteries with qua-functional heptafluorobutyric anhydride. *Angew. Chem., Int. Ed.* **133**, 20885-20890, (2021).

10 Guo, Y., Yao, Y., Guo, C., Song, Y., Huang, P., Liao, X., He, K., Zhang, H., Liu, H. & Hu, R. Atomistic observation and transient reordering of antisite Li/Fe defects toward sustainable $LiFePO_4$. *Energ. Environ. Sci.* **17**, 7749-7761, (2024).

11 Cui, C., Pang, B., Xu, S., Feng, J., Dong, H., Shang, M., Yu, L. & Dong, L. Enhanced photo-assisted lithium-ion batteries using natural dye-impregnated $LiFePO_4$ cathodes. *J. Mater. Chem. C*, (2025).

12 Lee, H., Lim, H.-S., Ren, X., Yu, L., Engelhard, M. H., Han, K. S., Lee, J., Kim, H.-T., Xiao, J. & Liu, J. Detrimental effects of chemical crossover from the lithium anode to cathode in rechargeable lithium metal batteries. *ACS Energy Lett.* **3**, 2921-2930, (2018).

13 Hekmatfar, M., Kazzazi, A., Eshetu, G. G., Hasa, I. & Passerini, S. Understanding the electrode/electrolyte interface layer on the Li-rich nickel manganese cobalt layered oxide cathode by XPS. *ACS Appl. Mater. Interfaces* **11**, 43166-43179, (2019).

14 Dedryvère, R., Maccario, M., Croguennec, L., Le Cras, F., Delmas, C. & Gonbeau, D. X-ray photoelectron spectroscopy investigations of carbon-coated Li x FePO4 materials. *Chem. Mater.* **20**, 7164-7170, (2008).

15 Bjerrum, J. *Stability Constants of Metal-ion Complexes, with Solubility Pro-ducts of Inorganic Substances*. (Chemical Society, 1957).

16 Tomaszewski, R. Citations to chemical resources in scholarly articles: CRC Handbook of Chemistry and Physics and The Merck Index. *Scientometrics* **112**, 1865-1879, (2017).

17 Das, B. & Baruah, J. B. Assemblies of cytosine within H-bonded network of adipic acid and citric acid. *J. Mol. Struct.* **1001**, 134-138, (2011).

18 Bretti, C., Crea, F., Foti, C. & Sammartano, S. Solubility and Activity Coefficients of Acidic and Basic Nonelectrolytes in Aqueous Salt Solutions. 2. Solubility and Activity Coefficients of Suberic, Azelaic, and Sebacic Acids in NaCl(aq), $(CH_3)_4$NCl(aq), and (C2H5)4NI(aq) at Different Ionic Strengths and at t = 25 °C. *J. Chem. Eng. Data* **51**, 1660-1667, (2006).

19 Weast, R. C. CRC handbook of chemistry and physics. (1986).

20 Braude, E. A. & Nachod, F. C. *Determination of organic structures by physical methods*. (Elsevier, 2013).

21 Hopff, H. & Krieger, A. Über decarboxylierung und dissoziation heterocyclischer dicarbonsäuren. *Helv. Chim. Acta* **44**, 1058-1063, (1961).

22 Silva, A. M., Kong, X. & Hider, R. C. Determination of the pKa value of the hydroxyl group in the α-hydroxycarboxylates citrate, malate and lactate by 13C NMR: implications for metal coordination in biological systems. *BioMetals* **22**, 771-778, (2009).

23 Wang, C., Chang, T., Yang, H. & Cui, M. Surface physiological changes induced by lactic acid on pathogens in consideration of pKa and pH. *Food Control* **46**, 525-531, (2014).

24 Dawson, R. & Elliott, W. Buffers and physiological media. *Data for biochemical research. Oxford University Press, Oxford*, 200-205, (1959).

25 Andrew, J. P. Handbook of polyethylene: Structures, properties and applications. *New York, jan*, (2000).

26 Kumler, W. & Eiler, J. J. The acid strength of mono and diesters of phosphoric acid. The n-alkyl esters from methyl to butyl, the esters of biological importance, and the natural guanidine phosphoric acids. *J. Am. Ceram. Soc.* **65**, 2355-2361, (1943).

27 De Robertis, A., De Stefano, C., Rigano, C. & Sammartano, S. Thermodynamic parameters for the protonation of carboxylic acids in aqueous tetraethylammonium iodide solutions. *J. Solution Chem.* **19**, 569-587, (1990).

28 Pfendt, L., Drazić, B., Popović, G., Drakulić, B., Vitnik, Z. & Juranić, I. Determination of all pKa values of some di-and tri-carboxylic unsaturated and epoxy acids and their polylinear correlation with the carboxylic group atomic charges. *J. Chem. Res.* **2003**, 247-248, (2003).

29 Lopalco, A., Lopedota, A. A., Laquintana, V., Denora, N. & Stella, V. J. Boric Acid, a Lewis Acid With Unique and Unusual Properties: Formulation Implications. *J. Pharm. Sci.* **109**, 2375-2386, (2020).

30 Davey, M. W., Montagu, M. V., Inze, D., Sanmartin, M., Kanellis, A., Smirnoff, N., Benzie, I. J. J., Strain, J. J., Favell, D. & Fletcher, J. Plant L-ascorbic acid: chemistry, function, metabolism, bioavailability and effects of processing. *J. Sci. Food Agric.* **80**, 825-860, (2000).

31 Brittain, H. G. *Profiles of drug substances, excipients, and related methodology*. Vol. 45 (Academic press, 2020).

32 Ji, B., Yan, K. & Sun, G. Investigation on functional properties of 1, 2, 3, 4-butanetetracarboxylic acid cross-linked fabrics impacted by molecular structures and chemical affinity of catalysts. *Ind. Eng. Chem. Res.* **55**, 5216-5222, (2016).

33 Bjerrum, J., Schwarzenbach, G. & Sillen, L. Stability Constants (The Chemical Society, London, 1958). *Part II*, (1957).

34 Mota, F. L., Queimada, A. J., Pinho, S. P. & Macedo, E. A. Aqueous solubility of some natural phenolic compounds. *Ind. Eng. Chem. Res.* **47**, 5182-5189, (2008).

35 Wang, M., Cai, Hao, Lu, Zifeng, Elgowainy, Amgad, Alam, Md Rakibul, Benavides, Pahola T., Benvenutti, Lívia, Burnham, Andrew, Do, Thai N., Farhad, Masum, Gan, Yu, Gracida-Alvarez, Ulises R., Hawkins, Troy R., Iyer, Rakesh K., Kar, Saurajyoti, Kelly, Jarod C., Kim, Taemin, Kolodziej, Christopher, Kwon, Hoyoung, Lee, Uisung, Lim, Juin Y., Liu, Xinyu, Morales, Michele, Ng, Clarence, Ou, Longwen, Pandey, Ishan, Shukla, Siddharth, Siddique, Nazib, Sun, Pingping, Sykora, Tom, Vyawahare, Pradeep, and Zhou, Jo. Greenhouse gases,. Greenhouse gases, Regulated Emissions, and Energy use in Technologies Model ® (2024 .Net). Computer Software. *USDOE Office of Energy Efficiency and Renewable Energy (EERE)*, (2024).

36 Kevin W. Knehr, J. J. K., Paul A. Nelson, and Shabbir Ahmed. Battery Performance and Cost Modeling for Electric-Drive Vehicles: A Manual for BatPaC v5.0. *ANL/CSE-22/1, July 2022*, (2022).

37 Dai, Q., Spangenberger, J., Ahmed, S., Gaines, L., Kelly, J. C. & Wang, M. EverBatt: A closed-loop battery recycling cost and environmental impacts model. (Argonne National Laboratory (ANL), Argonne, IL (United States), 2019).

38 Hu, X., An, A. K. J. & Chopra, S. S. Life Cycle Assessment of the Polyvinylidene Fluoride Polymer with Applications in Various Emerging Technologies. *ACS Sustain. Chem. Eng.* **10**, 5708-5718, (2022).

39 Foroughi, F., Rezvani Ghomi, E., Morshedi Dehaghi, F., Borayek, R. & Ramakrishna, S. A Review on the Life Cycle Assessment of Cellulose: From Properties to the Potential of Making It a Low Carbon Material. *Materials (Basel)* **14**, (2021).

40 Agency, E. P. *n-Methylpyrrolidone (NMP); Regulation Under the Toxic Substances Control Act (TSCA)*, 2024).

41 Agency, U. S. E. P. *Risk Evaluation for n-Methylpyrrolidone (NMP)*, 2025).

42 Choudhury, R., Wild, J. & Yang, Y. Engineering current collectors for batteries with high specific energy. *Joule* **5**, 1301-1305, (2021).

43 Tembo, P. M., Dyer, C. & Subramanian, V. Lithium-ion battery recycling—a review of the material supply and policy infrastructure. *NPG Asia Mater.* **16**, 43, (2024).

44 Zhang, X., Li, L., Fan, E., Xue, Q., Bian, Y., Wu, F. & Chen, R. Toward sustainable and systematic recycling of spent rechargeable batteries. *Chem. Soc. Rev.* **47**, 7239-7302, (2018).

45 Song, L., Qi, C., Wang, S., Zhu, X., Zhang, T., Jin, Y. & Zhang, M. Direct regeneration of waste $LiFePO_4$ cathode materials with a solid-phase method promoted by activated CNTs. *Waste Manag.* **157**, 141-148, (2023).

46 Rey, I., Vallejo, C., Santiago, G., Iturrondobeitia, M. & Lizundia, E. Environmental Impacts of Graphite Recycling from Spent Lithium-Ion Batteries Based on Life Cycle Assessment. *ACS Sustain. Chem. Eng.* **9**, 14488-14501, (2021).

47 Fan, E., Li, L., Wang, Z., Lin, J., Huang, Y., Yao, Y., Chen, R. & Wu, F. Sustainable Recycling Technology for Li-Ion Batteries and Beyond: Challenges and Future Prospects. *Chem. Rev.* **120**, 7020-7063, (2020).

48 Zhang, X., Xue, Q., Li, L., Fan, E., Wu, F. & Chen, R. Sustainable Recycling and Regeneration of Cathode Scraps from Industrial Production of Lithium-Ion Batteries. *ACS Sustain. Chem. Eng.* **4**, 7041-7049, (2016).

49 Ji, Y., Jafvert, C. T., Zyaykina, N. N. & Zhao, F. Decomposition of PVDF to delaminate cathode materials from end-of-life lithium-ion battery cathodes. *J. Clean. Prod.* **367**, 133112, (2022).

50 Pu, X., Zhang, S., Zhao, D., Xu, Z.-L., Chen, Z. & Cao, Y. Building the robust fluorinated electrode–electrolyte interface in rechargeable batteries: from fundamentals to applications. *Electrochem. Energy Rev.* **7**, 21, (2024).

51 Bertilsson, S., Larsson, F., Furlani, M., Albinsson, I. & Mellander, B.-E. Lithium-ion battery electrolyte emissions analyzed by coupled thermogravimetric/Fourier-transform infrared spectroscopy. *J. Power Sources* **365**, 446-455, (2017).

52 Ahmed, S., Nelson, P. A., Gallagher, K. G. & Dees, D. W. Energy impact of cathode drying and solvent recovery during lithium-ion battery manufacturing. *J. Power Sources* **322**, 169-178, (2016).

53 Wood, D. L., Quass, J. D., Li, J., Ahmed, S., Ventola, D. & Daniel, C. Technical and economic analysis of solvent-based lithium-ion electrode drying with water and NMP. *Dry. Technol.* **36**, 234-244, (2018).

54 Léger, M., La Monaca, A., Basu, N. & Demopoulos, G. P. Alternatives assessment of polyvinylidene fluoride-compatible solvents for N-methyl pyrrolidone substitution in lithium-ion battery cathodes. *Next Sustainability* **5**, 100084, (2025).

55 Sarkar, A., May, R., Ramesh, S., Chang, W. & Marbella, L. E. Recovery and reuse of composite cathode binder in lithium ion batteries. *ChemistryOpen* **10**, 545-552, (2021).

56 Ahuis, M., Aluzoun, A., Keppeler, M., Melzig, S. & Kwade, A. Direct recycling of lithium-ion battery production scrap – Solvent-based recovery and reuse of anode and cathode coating materials. *J. Power Sources* **593**, 233995, (2024).
