# Supplementary material for: Fluorine-free polymer binder enables circular manufacturing and recycling of lithium-ion batteries
Source: Nat Commun. 2026 Oct 9;17:11473. doi: 10.1038/s41467-026-78215-3 (PMC13740254; doi:10.1038/s41467-026-78215-3)
Supplement: Supplementary file 2 [file 41467_2026_78215_MOESM2_ESM.pdf]

nature portfolio

Peer Review File

Fluorine-free polymer binder enables circular manufacturing and recycling of lithium-ion batteries

Corresponding Author: Professor Zheng Chen

This file contains all reviewer reports in order by version, followed by all author rebuttals in order by version.

Version 0:

Reviewer comments:

Reviewer #1

(Remarks to the Author)
The authors present a binder copolymer that combines the advantages of both building blocks while negating their disadvantages. Both building blocks are well-known and have already been investigated many times. Combining them inherently in a copolymer can be considered a reasoned and novel idea.
The authors primarily compare their results with those of the widely used PVDF binder. They provide a detailed comparison regarding electrochemical performance, processability, and mechanical stability. Most results are presented in a well-thought-out manner; however, some structural changes would improve clarity:
On pages 6-7, the authors discuss bonding mechanisms, particle sizes, and agglomerate formation, and claim superior mechanical stability. The data and images supporting the hypothesis of superior mechanical stability, however, are presented in the section discussing the electrochemical properties. They should be included in the section before.
In paragraphs 260-272, the authors refer to the established pre-treatment protocol for electrode delamination. The reference, though, presents a soaking in NMP, which would contradict the concept of sustainability. In the Methods section, they describe the delamination procedure and the use of water to delaminate cathodes containing the novel binder copolymer. This information should be included in the main paragraph to improve clarity.
Additional remarks:
The processability of recyclate CAM is a significant challenge; the authors should provide rheological data after the direct recycling process to support their claims.
The authors claim the superior processability of the PCC binder and therefore provide rheological data. However, the claim of processability based on slurry viscosity at a single shear rate is insufficient. Depending on the coating technology used (e.g., slot die, doctor blade), shear rates may change significantly, thereby affecting the processability of the electrode slurry. Here, the authors should provide a complete flow diagram with shear rates ranging from 0.1 1/s to at least 100 1/s.
The unit for shear rates is 1/s.

Overall, the authors provide a well-structured, well-designed report of their work.

Reviewer #2

(Remarks to the Author)
I co-reviewed this manuscript with one of the reviewers who provided the listed reports. This is part of the Nature Communications initiative to facilitate training in peer review and to provide appropriate recognition for Early Career Researchers who co-review manuscripts.

Reviewer #3

(Remarks to the Author)
This study investigates a fluorine-free, water-processable polymer binder (PCC) for circular manufacturing and recycling of lithium-ion batteries. The authors combine polymer chemistry, electrochemistry, advanced characterization, in-situ impedance analysis, recycling demonstrations, and life-cycle assessment (LCA) to support PCC as a viable alternative to PVDF/NMP systems.
The study is technically robust and relevant to sustainable battery manufacturing. The supplementary information substantiates most claims made in the main manuscript. This work could significantly influence both academic and industrial

battery sectors.
However, several aspects need clearer justification, more rigorous quantitative analysis, and cautious interpretation, particularly regarding mechanistic claims, LCA assumptions, and long-term generalizability.
The work is technically competent and clearly the product of significant experimental effort. With substantial revision and intellectual tightening, it may become suitable for publication. Please include these comments and suggestions for improvement or better clarification.
1. The link between CA crosslink density and electrochemical behavior is inferred mainly from DSC and DRT trends. The "optimal" CA content (5–8 wt%) is justified thermally, but its direct electrochemical mechanistic role remains unclear. Clarify whether performance gains arise primarily from: Mechanical cohesion? Interfacial chemistry? Ionic transport through binder domains? Consider adding or explicitly discussing: Binder swelling/electrolyte uptake measurements, Mechanical modulus vs. cycling stability correlations.
2. Acknowledge uncertainty in distinguishing crosslinking vs. degradation in DSC. Clarify whether DSC is used qualitatively or quantitatively to infer crosslink density. Consider rephrasing conclusions about "maximum crosslink density" to avoid overstatement.
3. Include a concise table summarizing: RSEI and Rct evolution (initial vs. end-of-life). Discuss potential limitations of DRT peak assignment, especially in porous composite electrodes. Explicitly state whether electrolyte composition and formation protocols were identical across binder systems.
4. Avoid implying direct causality between PVDF and amorphous layer thickness without: Chemical identification (e.g., EELS, XPS depth profiling). Reframe conclusions to emphasize correlation and consistency, not definitive mechanism.
5. Some assumptions (e.g., NMP recovery rate, fluorine volatilization fraction, binder removal efficiency) strongly influence results. These assumptions are sometimes presented as fixed values rather than ranges. Recommendations: Explicitly include: Sensitivity analysis or uncertainty ranges Discussion of regional or technological variability Clarify that the LCA reflects a scenario analysis, not a universal industrial reality.
Minor Comments
o Clearly define PCC early and use consistently.
o Avoid switching between "PCC", "PC + CA + LiOH", etc., without reminders.
o A few passages use promotional phrasing ("superior", "dramatic", "decisive").
o Recommend adopting a more neutral academic tone in these sections.

Reviewer #4

(Remarks to the Author)
This manuscript presents a comprehensive narrative that bridges molecular binder design to system-level life-cycle assessment (LCA), offering a clear solution to the critical recycling challenges associated with PVDF. I commend the high completeness of the work—particularly the demonstration of impurity-free recycling—and would like to highlight both the aspects I found especially impressive and the areas that would benefit from further refinement. However, to fully justify publication in Nature Communications, critical issues regarding the chemical novelty and processability on Ni-rich cathodes must be addressed.

1) Stability on NCM811. To substantiate the claim of a "universal platform" for Ni-rich cathodes, the authors should address the risk of slurry gelation. Please provide time-dependent viscosity data (e.g., 0 h, 6 h, 24 h) for the NCM811–PCC slurry to demonstrate stability during storage and casting.

2) Verification of chemical novelty. To prove the necessity of the specific "esterification crosslinking" chemistry beyond what can be achieved with conventional PAA/CMC-type binders, please include a head-to-head performance comparison against a simple physical mixture (PAA/CMC/CA without esterification).

3) Durability at high temperature and voltage. LSV data alone is insufficient for practical validation of oxidative stability. Please add leakage-current measurements (floating test) or cycling data at elevated temperature (>45 °C) and high cut-off voltage (4.5 V) to confirm stability under harsh operating conditions

Version 1:

Reviewer comments:

Reviewer #1

(Remarks to the Author)
The authors have done a good job of addressing all concerns. This manuscript is acceptable for publication now as is.

Reviewer #2

(Remarks to the Author)
I co-reviewed this manuscript with one of the reviewers who provided the listed reports. This is part of the Nature Communications initiative to facilitate training in peer review and to provide appropriate recognition for Early Career Researchers who co-review manuscripts.

Reviewer #4

(Remarks to the Author)
The reviewer's comments have been adequately addressed, and the manuscript has been improved accordingly. I therefore recommend acceptance of this manuscript.

I have reviewed the authors' responses to Reviewer 3's comments.

Overall, the authors appear to have adequately addressed the major concerns raised by Reviewer 3.

In particular, they provided additional electrochemical data to support the correlation between CA crosslinking and electrochemical performance.
They also revised the interpretation of the DSC and DRT results by avoiding overly quantitative or definitive statements and reframing them as qualitative or comparative analyses.
In addition, depth-resolved XPS data were added to support the discussion of the PVDF-related interphase, and the LCA section was clarified as a scenario-based comparative analysis.
The terminology and overly promotional expressions also appear to have been appropriately revised.

However, I believe that Supplementary Table 5 requires final confirmation.
In the authors' response, they state that RSEI and Rct were summarized, whereas the actual table is labeled with Rs and Rct.
Therefore, it should be clarified whether Rs was intended to represent RSEI.
In addition, the PCC end-of-life Rs value is reported as 0 Ω, which should be checked to determine whether it is a typographical error, a fitting artifact, or a negligible fitted value.

In summary, I consider the rebuttal suitable for further consideration.
If the minor issue regarding the Rs/RSEI notation and the PCC end-of-life Rs value in Supplementary Table 5 is clarified, I do not see any major remaining problem with the authors' response to Reviewer 3.

Open Access This Peer Review File is licensed under a Creative Commons Attribution 4.0 International License, which permits use, sharing, adaptation, distribution and reproduction in any medium or format, as long as you give appropriate credit to the original author(s) and the source, provide a link to the Creative Commons license, and indicate if changes were made.
In cases where reviewers are anonymous, credit should be given to 'Anonymous Referee' and the source.
The images or other third party material in this Peer Review File are included in the article's Creative Commons license, unless indicated otherwise in a credit line to the material. If material is not included in the article's Creative Commons license and your intended use is not permitted by statutory regulation or exceeds the permitted use, you will need to obtain permission directly from the copyright holder.
To view a copy of this license, visit https://creativecommons.org/licenses/by/4.0/

Point-by-point response to Reviewer 1's comments

Overall Comments: The authors present a binder copolymer that combines the advantages of both building blocks while negating their disadvantages. Both building blocks are well-known and have already been investigated many times. Combining them inherently in a copolymer can be considered a reasoned and novel idea. The authors primarily compare their results with those of the widely used PVDF binder. They provide a detailed comparison regarding electrochemical performance, processability, and mechanical stability. Most results are presented in a well-thought-out manner; however, some structural changes would improve clarity. Overall, the authors provide a well-structured, well-designed report of their work.

Reply: We thank the reviewer for the thorough and constructive evaluation of our manuscript. We appreciate the recognition of the rationale and novelty of combining the two well-established building blocks into a single copolymer binder, as well as the positive assessment of the comparative analysis with PVDF in terms of electrochemical performance, processability, and mechanical stability.

In response to the reviewer's suggestion regarding clarity, we have revised the manuscript structure to improve the logical flow of the presentation. We believe these changes further strengthen the clarity and readability of the manuscript while preserving the technical rigor of the study.

Comment 1): On pages 6-7, the authors discuss bonding mechanisms, particle sizes, and agglomerate formation, and claim superior mechanical stability. The data and images supporting the hypothesis of superior mechanical stability, however, are presented in the section discussing the electrochemical properties. They should be included in the section before.

Reply 1): We thank the reviewer for this constructive comment. We agree that the discussion of bonding mechanisms, particle size effects, and agglomerate formation should be directly supported by mechanical evidence to improve clarity and logical flow.

In response, we have reorganized the manuscript so that the data supporting the claim of superior mechanical stability, including electrode morphology, adhesion behavior, and mechanical integrity during processing, are now presented immediately following the discussion of binder-particle interactions and agglomerate formation, as shown in **Figure 2**. The electrochemical performance section has been correspondingly revised to focus on electrochemical behavior, with appropriate cross-references to the preceding mechanical characterization section. We hope this restructuring improves the coherence of the argument and ensures that claims regarding mechanical stability are supported by experimental evidence at the point where they are introduced. (**Pages 6-8 of Main text**)

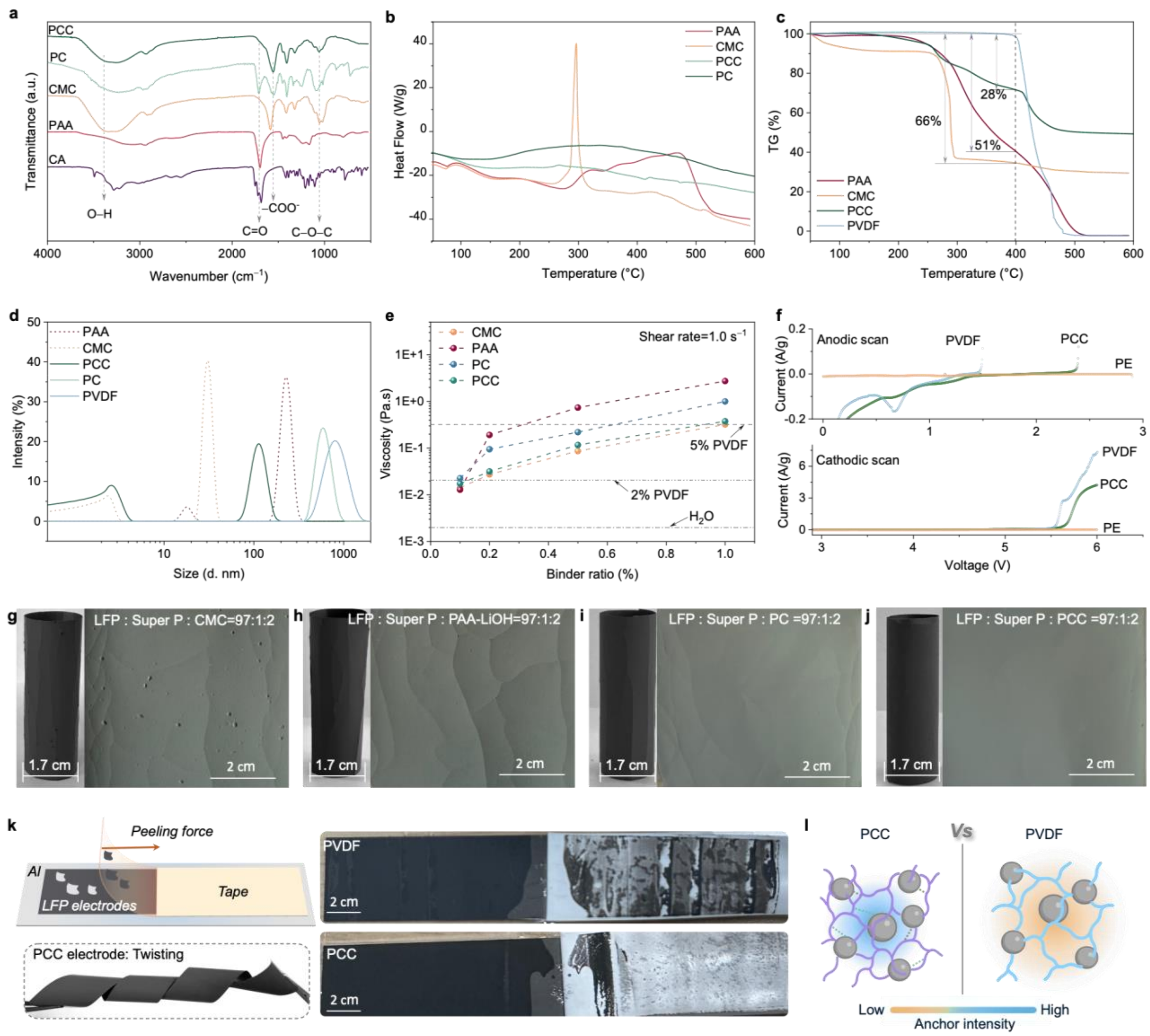

Fig. 2: Characterizations of the PCC binder system. **a**, FTIR spectra of PAA, CMC, CA, and various mixtures. **b**, DSC profile of pristine PAA powder, pristine CMC powder, PAA and CMC (PC) mixture, and PCC mixture. **c,** TGA thermograms of PAA, CMC, PCC, and PVDF. **d**, Size distributions of PAA, CMC, PC, PCC, and PVDF/NMP solutions, all the water-soluble binder was prepared in 1 wt. %, while PVDF was prepared in 0.2 wt.% due to the large particle size leading to poor dispersion. **e**, Viscosity comparison of various binder solutions (CMC, PAA, PC, PCC) at different concentrations (wt%). Data was selected from a shear rate of 1 s^{-1} for comparison. Dashed lines mark the viscosity range of conventional 2% and 5% PVDF solutions in NMP, as well as pure water, for comparison. A complete flow diagram with shear rates ranging from 0.1-15 s^{-1} is provided in supporting information (Supplementary Fig. 5). **f**, Linear sweep voltammetry of electrodes prepared with PVDF, PCC, and pure electrolyte (PE) under anodic and cathodic scans. **g-j,** Digital photographs of LFP electrodes prepared with different binders: **(g)** CMC, **(h)** PAA-LiOH, **(i)** PC, and **(j)** PCC. All electrodes were prepared with a fixed composition of LFP: Super P: Binder = 97:

1: 2 by weight and cast on carbon-coated Al foil under identical conditions. **k**, Schematic and photographic illustration of tape-peeling tests comparing PVDF and PCC electrodes, alongside a twisting test for PCC electrodes. The two left panels show the initial peel. **l**, Schematic comparison of the polymer-particle interactions in PCC and PVDF. PCC- and PVDF-based electrodes. The color gradient denotes relative anchoring strength.

Comment 2): In paragraphs 260-272, the authors refer to the established pre-treatment protocol for electrode delamination. The reference, though, presents a soaking in NMP, which would contradict the concept of sustainability. In the Methods section, they describe the delamination procedure and the use of water to delaminate cathodes containing the novel binder copolymer. This information should be included in the main paragraph to improve clarity.

Reply 2): We thank the reviewer for pointing out the need for clarification. We agree that the original wording could lead to ambiguity regarding the delamination solvent. In this study, different delamination protocols were intentionally applied depending on the binder chemistry. For control electrodes employing conventional PVDF binders, we followed the reported NMP-based delamination protocol as described in reference[1]. In contrast, for electrodes fabricated with the F-free, water-processable PCC binder, delamination was carried out exclusively using deionized water, enabled by the intrinsic water solubility of the PCC binder.

We have revised the main text to explicitly state this distinction, thereby ensuring consistency with the Methods section and clearly highlighting the sustainability advantage introduced by the PCC binder system. (**Page 12 of Main text**)

Page 12 of Main text

Following cell disassembly, cycled LFP and graphite electrodes (denoted as "cLFP" and "cGr") were collected for regeneration. PVDF-bound electrodes delamination was performed using NMP following established pre-treatment protocols[1]. In contrast, PCC-bound electrodes were delaminated by mild soaking in deionized water, leveraging the intrinsic water solubility of PCC binder to enable efficient separation of active materials from the current collectors.

Comment 3): Additional remarks: The processability of recyclate CAM is a significant challenge; the authors should provide rheological data after the direct recycling process to support their claims. The authors claim the superior processability of the PCC binder and therefore provide rheological data. However, the claim of processability based on slurry viscosity at a single shear rate is

insufficient. Depending on the coating technology used (e.g., slot die, doctor blade), shear rates may change significantly, thereby affecting the processability of the electrode slurry. Here, the authors should provide a complete flow diagram with shear rates ranging from 0.1 1/s to at least 100 1/s. The unit for shear rates is 1/s.

Reply 3): We thank the reviewer for this technically important comment. We agree that slurry processability cannot be adequately assessed based on viscosity measured at a single shear rate, as practical coating techniques such as doctor-blade and slot-die coating span a broad range of shear conditions.

In response, we have expanded the rheological characterization to include full shear-rate-dependent flow curves rather than reporting viscosity at a single point. Due to instrument torque constraints of the Discovery Hybrid Rheometer HR-3 under the tested geometries and slurry formulations, the experimentally accessible shear-rate window is limited to 0.1-15 s^{-1} (**Supplementary Figs. 5, 22, and 31**). Nevertheless, we believe this range captures shear conditions relevant to slurry mixing, leveling, and laboratory-scale doctor-blade coating, and enables a consistent comparative evaluation of binder processability across different binder systems. We explicitly acknowledge in the revised manuscript that industrial slot-die coating may involve substantially higher shear rates (≥100 s^{-1}). Accordingly, our discussion of processability is framed in terms of comparative rheological trends within the experimentally accessible and application-relevant regime, rather than extrapolation beyond measured conditions. In addition, the unit of shear rate has been corrected to s^{-1} throughout the manuscript (**Figure 2e**).

Regarding the reviewer's concern about the processability of recycled cathode active materials, we agree that rheological validation after direct recycling is important. Accordingly, we have added shear-rate-dependent rheological measurements for aqueous slurries prepared using directly recycled LFP with the PCC binder system (**Supplementary Fig. 31**). For comparison, rheological data obtained using the conventional PVDF system are also provided. As shown in **Supplementary Fig. 31**, across the measured shear-rate window, slurries prepared from recovered pristine LFP (pLFP) and recycled LFP (rLFP) exhibit viscosity profiles that closely track those of their pristine counterparts, indicating that the recovery process does not introduce significant changes in slurry rheology. Both pLFP and rLFP slurries show comparable shear-dependent behavior, with viscosity decreasing with increasing shear rate, consistent with shear-thinning flow typical of battery electrode slurries. Minor differences observed between pristine and recovered samples at low shear rates are attributed to subtle variations in particle surface condition or agglomeration state introduced during cycling and recovery. However, these differences diminish at higher shear rates, which are more relevant to slurry mixing and coating processes. Importantly, the relative ordering of viscosity among different binder formulations is preserved for both pristine and recovered materials. Overall, the viscosity data indicate that the recovered materials from PCC system retain slurry processability comparable to pristine materials, supporting their compatibility

with aqueous electrode fabrication processes. The corresponding data and discussion have been incorporated into the revised manuscript.

We appreciate the reviewer's emphasis on rigorous rheological evaluation, which has strengthened the technical completeness of the manuscript. (**Page 6 of Main text**; **Pages 12-13, 34-35, and 44-45 of Supporting Information**)

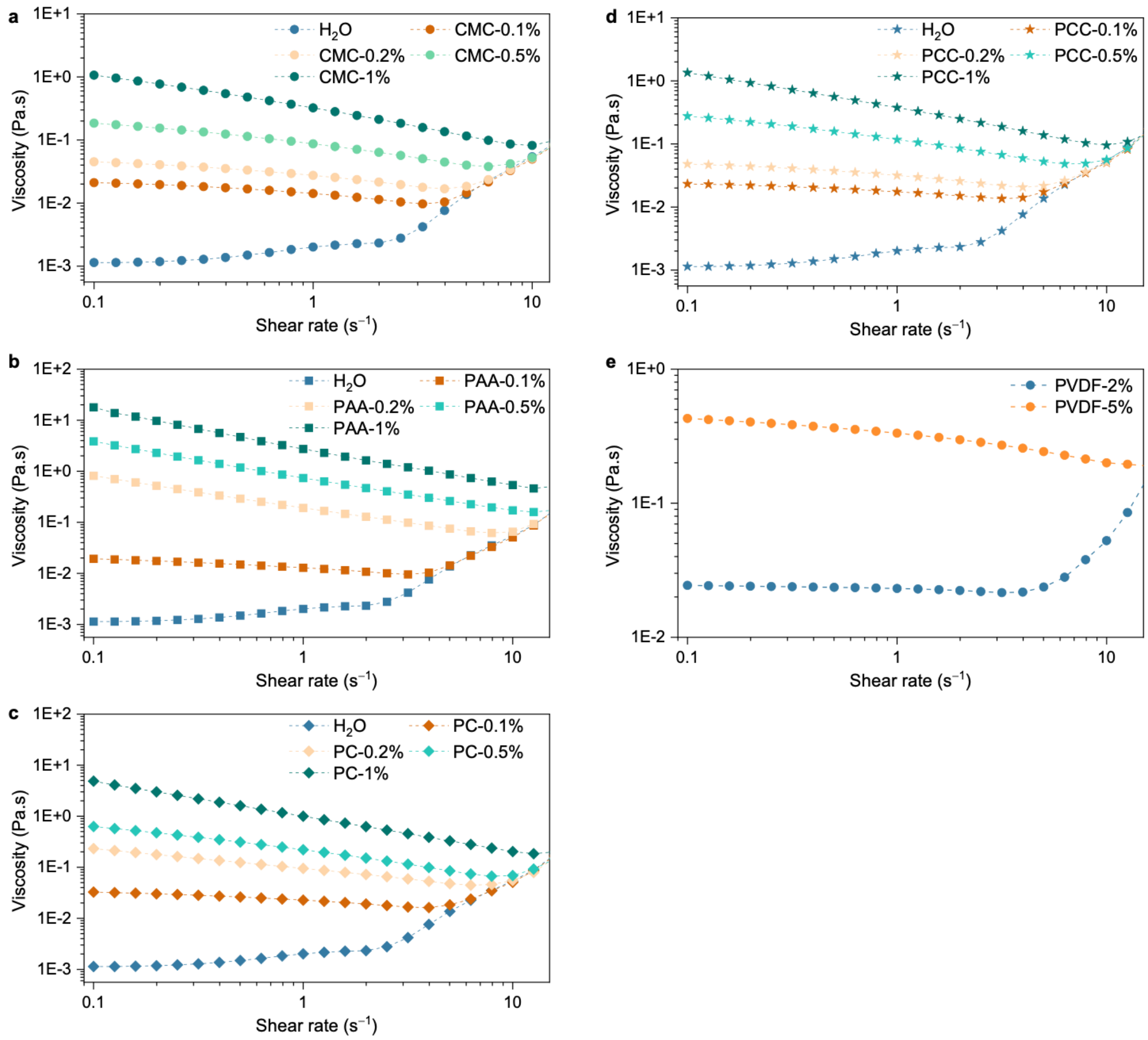

Supplementary Figure 5: **Viscosity behavior of different binder solutions and dispersion appearance.** Viscosity of different binders as a function of shear rate at different concentrations (wt.%) for (**a**) CMC, (**b**) PAA (pH adjusted to ~6 using LiOH), (**c**) PC (pH adjusted to ~6 using LiOH), and (**d**) PCC. For water-soluble binder systems, the viscosity of pure water is included as a reference. (**e**) Viscosity of PVDF solutions in NMP at 2 wt% and 5 wt%.

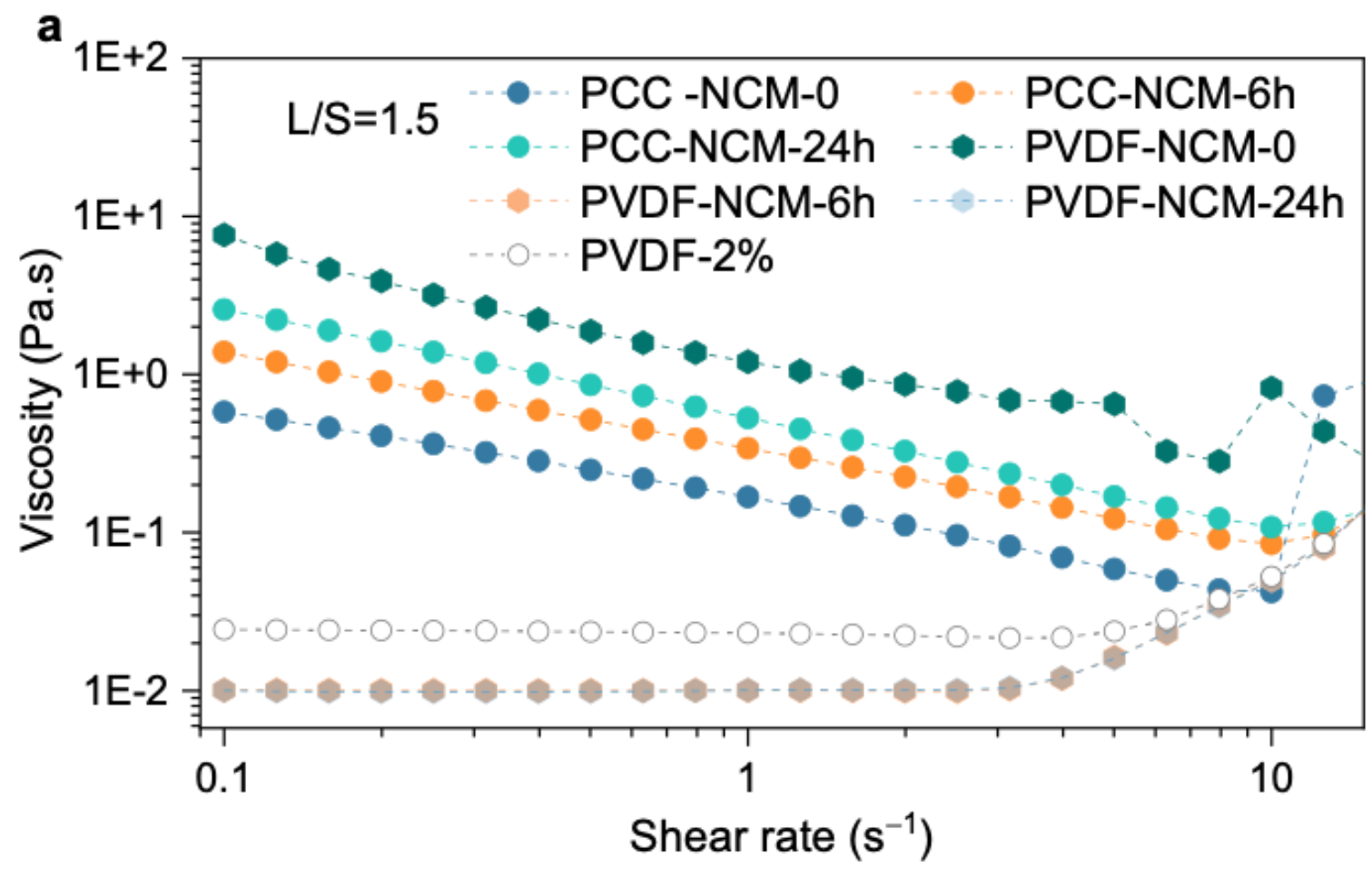

Supplementary Figure 22: Time-dependent viscosity and dispersion behavior of NCM811 slurries. a, Viscosity evolution of NCM811 slurries prepared with PCC and PVDF binders as a function of holding time (0, 6, and 24 h), measured under identical formulation conditions (liquid-to-solid ratio, L/S = 1.5; carbon additive omitted to isolate binder-NCM interactions) and identical rheological measurement settings. At the highest shear rates, a slight viscosity upturn is observed for the PVDF-NCM-0 and PCC-NCM-O samples, which is attributed to measurement artifacts arising from edge fracture or high-stress instability rather than intrinsic shear thickening behavior.

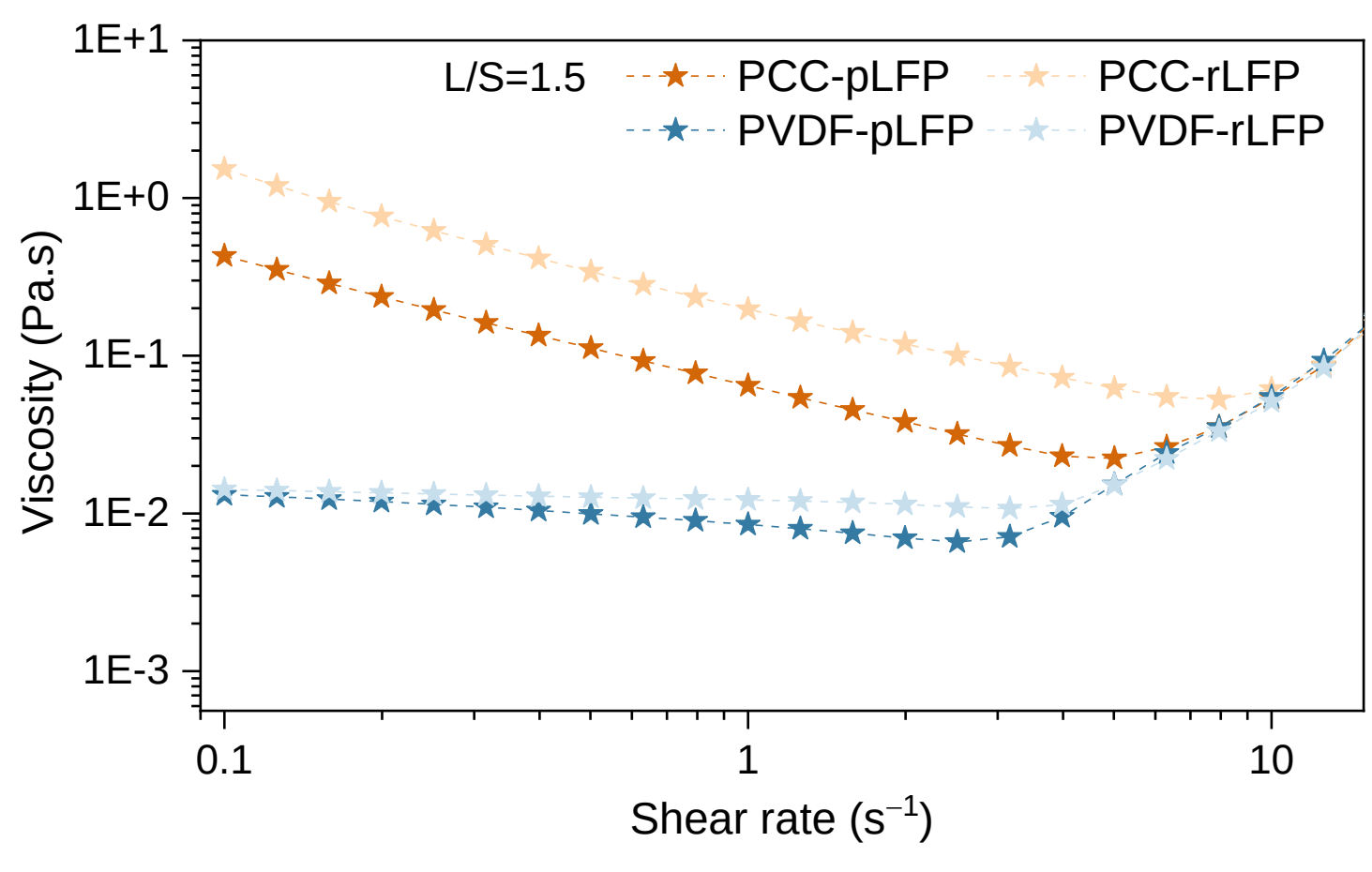

Supplementary Figure 31: **Time-dependent viscosity and dispersion behavior of LFP slurries.** Viscosity evolution of various LFP slurries prepared with PCC and PVDF binders as a function of holding time (1 h), measured under identical formulation conditions (liquid-to-solid ratio, L/S = 1.5; carbon additive omitted to isolate binder-LFP interactions) and identical rheological measurement settings.

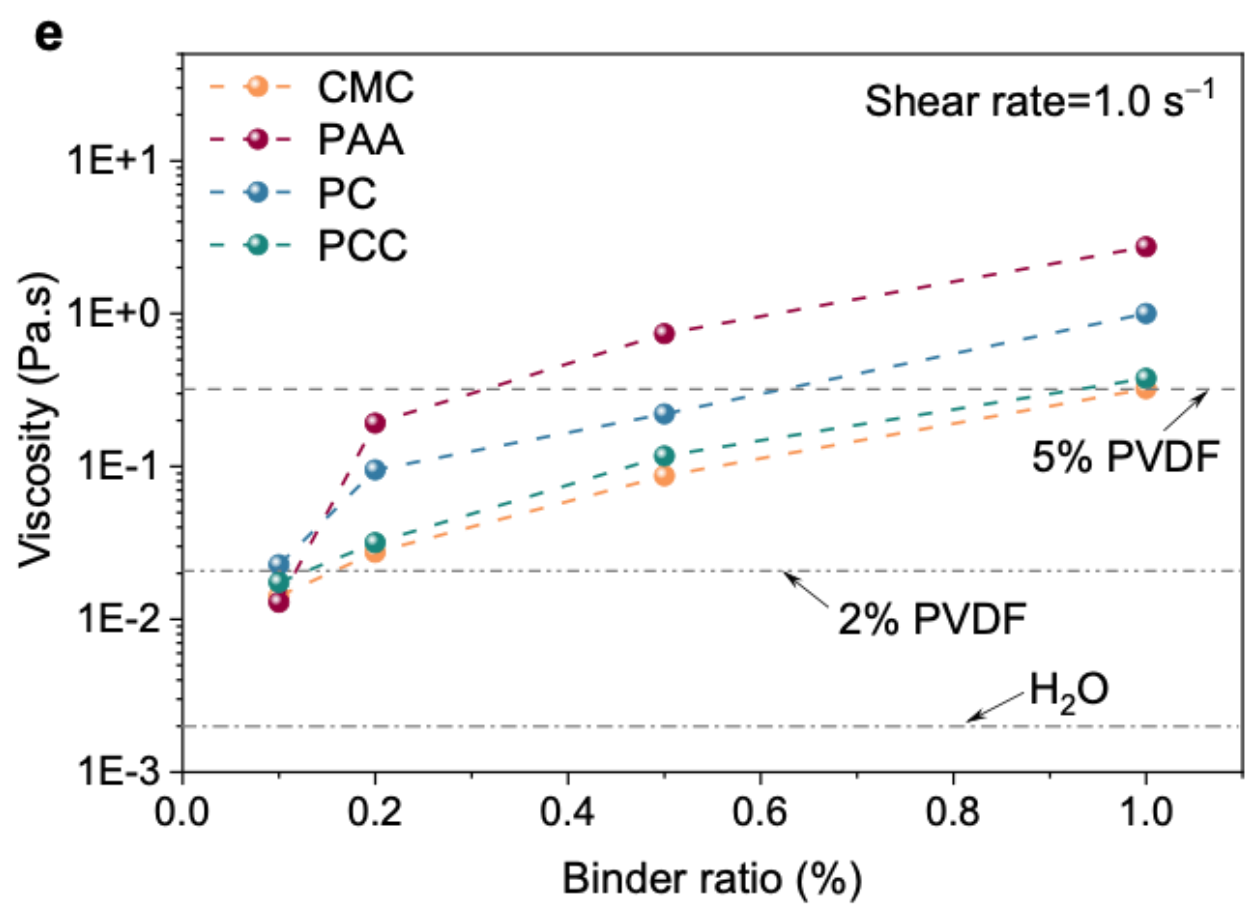

Fig. 2: Characterizations of the PCC binder system. **e**, Viscosity comparison of various binder solutions (CMC, PAA, PC, PCC) at different concentrations (wt.%). Data was selected from a shear rate of 1 s^{-1} for comparison. Dashed lines mark the viscosity range of conventional 2 wt.% and 5 wt.% PVDF solutions in NMP, as well as pure water, for comparison. A complete flow diagram with shear rates ranging from 0.1-15 s^{-1} was provided in supporting information.

Point-by-point response to Reviewer 2's comments

Overall Comments: I co-reviewed this manuscript with one of the reviewers who provided the listed reports. This is part of the Nature Communications initiative to facilitate training in peer review and to provide appropriate recognition for Early Career Researchers who co-review manuscripts.

Reply: We thank the reviewer for the positive assessment of our manuscript and for the recommendation for publication in Nature Communications following revision. We also appreciate the transparency regarding the co-review process and the initiative to support training and recognition of Early Career Researchers in peer review.

Point-by-point response to Reviewer 3's comments

Overall Comments: This study investigates a fluorine-free, water-processable polymer binder (PCC) for circular manufacturing and recycling of lithium-ion batteries. The authors combine polymer chemistry, electrochemistry, advanced characterization, *in-situ* impedance analysis, recycling demonstrations, and life-cycle assessment (LCA) to support PCC as a viable alternative to PVDF/NMP systems. The study is technically robust and relevant to sustainable battery manufacturing. The supplementary information substantiates most claims made in the main manuscript. This work could significantly influence both academic and industrial battery sectors. However, several aspects need clearer justification, more rigorous quantitative analysis, and cautious interpretation, particularly regarding mechanistic claims, LCA assumptions, and long-term generalizability. The work is technically competent and clearly the product of significant experimental effort. With substantial revision and intellectual tightening, it may become suitable for publication. Please include these comments and suggestions for improvement or better clarification.

Reply: We thank the reviewer for the thorough and thoughtful evaluation of our manuscript. We appreciate the recognition of the technical rigor of the study, the integration of polymer chemistry, electrochemistry, advanced characterization, in situ impedance analysis, recycling demonstrations, and life-cycle assessment, as well as the relevance of this work to sustainable battery manufacturing in both academic and industrial contexts.

We also acknowledge the reviewer's concerns regarding the need for clearer justification, more rigorous quantitative analysis, and cautious interpretation in certain areas, particularly with respect to mechanistic discussions, LCA assumptions, and the generalizability of the conclusions. Accordingly, we have carefully revised the manuscript to strengthen the quantitative basis of our claims, clarify underlying assumptions, and refine the language to avoid overinterpretation where appropriate. Additional analyses, clarifications, and cross-references to the Supplementary Information have been incorporated to improve transparency and robustness.

Comment 1): The link between CA crosslink density and electrochemical behavior is inferred mainly from DSC and DRT trends. The "optimal" CA content (5–8 wt%) is justified thermally, but its direct electrochemical mechanistic role remains unclear. Clarify whether performance gains arise primarily from: Mechanical cohesion? Interfacial chemistry? Ionic transport through binder domains? Consider adding or explicitly discussing: Binder swelling/electrolyte uptake measurements, Mechanical modulus vs. cycling stability correlations.

Reply 1): We thank the reviewer for this insightful comment. We agree that, in the original version, the correlation between CA crosslink density and electrochemical behavior was primarily inferred from DSC and DRT analyses, without sufficiently clarifying the direct electrochemical mechanism.

It is important to note that the mechanical modulus of isolated binder films cannot fully represent the mechanical behavior of practical composite electrodes, because electrode integrity is governed by the coupled interactions among binder, active material particles, and conductive additives. Therefore, to better elucidate the mechanistic role of CA crosslinking in realistic electrode environments, we incorporated additional electrochemical and physicochemical analyses.

First, electrolyte uptake measurements of free-standing PCC films with varying CA contents have been added (**Supplementary Fig. 3b**). These results show a systematic decrease in solvent uptake with increasing CA addition, confirming that CA content directly regulates polymer network density and swelling behavior. At low CA content (2 wt.%), excessive swelling indicates a loosely crosslinked network that may undermine mechanical cohesion and interfacial stability. Conversely, at high CA content (≥10 wt.%), the polymer network becomes overly compact, restricting electrolyte penetration and limiting Li^+ accessibility within the electrode matrix.

Second, GITT analysis (**Supplementary Fig. 3c**) further demonstrates that electrodes with low-to-intermediate CA contents (2-8 wt%) exhibit higher Li^+ diffusion coefficients than highly crosslinked systems, indicating that excessive crosslink density introduces additional transport resistance. Finally, cycling results (**Supplementary Fig. 3d**) confirm an optimal CA window of 5-8 wt%, where discharge capacity and stability are maximized.

Collectively, these results establish a clear structure-property-performance relationship within the PCC system. The improved performance at intermediate CA content arises from a balanced regulation of polymer network integrity and ionic transport through the binder domain. We have revised the manuscript to explicitly clarify this mechanistic framework and to emphasize that the optimal CA content is defined by coupled mechanical and electrochemical considerations rather than thermal trends alone. (**Page 5 of Main text**, **Pages 8-10 of Supporting Information**)

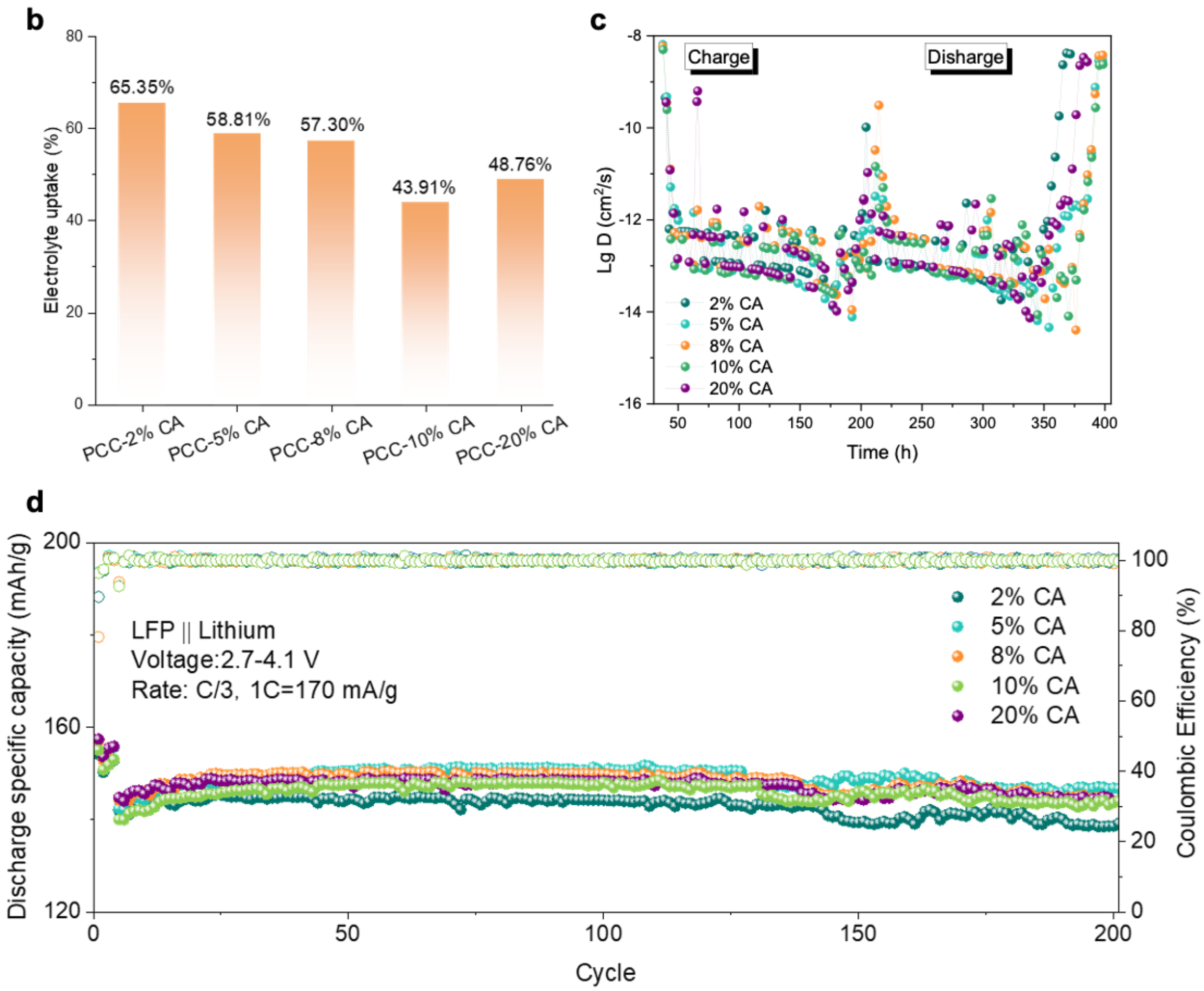

Supplementary Figure 3: Effect of citric acid (CA) content on PCC binder properties and electrochemical performance. b, Electrolyte uptake of PCC binder films with varying CA contents, illustrating the influence of crosslinking density on solvent absorption behavior**. c,** Galvanostatic intermittent titration technique (GITT) profiles during charge and discharge for electrodes prepared with different CA loadings, comparing Li^+ diffusion behavior. GITT measurements were conducted in the second cycle following first-cycle activation at C/20 for all samples. **d,** Long-term cycling stability of electrodes employing PCC binders with varying CA contents, highlighting the impact of crosslinking level on capacity retention and electrochemical durability**.**

Comment 2): Acknowledge uncertainty in distinguishing crosslinking vs. degradation in DSC. Clarify whether DSC is used qualitatively or quantitatively to infer crosslink density. Consider rephrasing conclusions about "maximum crosslink density" to avoid overstatement.

Reply 2): We thank the reviewer for this important clarification. We agree that DSC alone cannot unambiguously distinguish between crosslinking reactions and thermally induced degradation, and that our original wording could be interpreted as overly quantitative.

In the revised manuscript, we explicitly clarify that DSC is used qualitatively to assess relative changes in the thermal response and apparent network evolution of the PCC binders, rather than as a direct or quantitative measure of crosslink density. We have revised the text to acknowledge the inherent uncertainty in separating crosslinking from early-stage degradation based solely on DSC profiles. Accordingly, statements referring to "maximum crosslink density" have been removed and replaced with descriptions of the observed thermal behavior, without assigning a specific structural interpretation. These revisions ensure a more balanced interpretation of the DSC data and avoid overstatement of conclusions drawn from thermal analysis alone. (**Pages 4-7 of Supporting Information**)

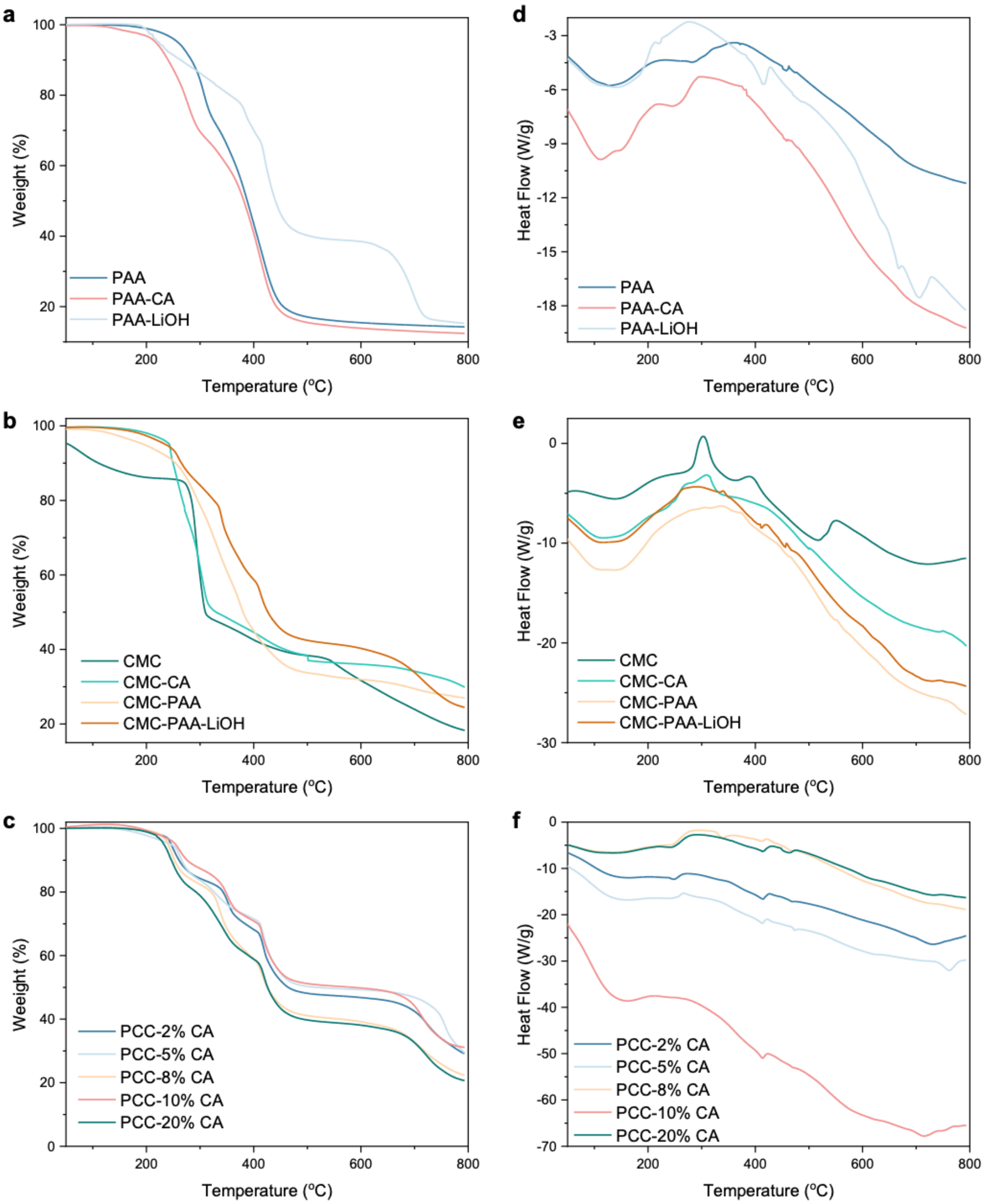

Supplementary Figure 2: Thermal behaviour of binder systems. a, b, c, Thermogravimetric analysis of the binder formulations indicated in the legends. **d, e, f,** Corresponding heat-flow traces for the same samples. All measurements were conducted under identical conditions; exotherm/endotherm directions and heating rates follow the instrument convention. DSC results

are used qualitatively to assess relative changes in the thermal response and apparent network evolution of the PCC binders, rather than as a direct or quantitative measure of crosslink density.

The thermal properties of PAA, PAA-CA, and PAA-LiOH binders were evaluated using differential scanning calorimetry (DSC) and thermogravimetric analysis (TGA) to elucidate how chemical modification influences the thermal response of the binder system (**Supplementary Fig. 2a, d**). Pure PAA remains thermally stable below ~250 °C, followed by an initial mass loss stage from ~250-320 °C and a major decomposition step between ~320 and 430 °C, consistent with progressive polymer backbone degradation. In contrast, PAA-CA displays a pronounced heat-flow feature in the range of ~150-200 °C, which is likely associated with thermally activated esterification and crosslinking reactions between CA and PAA chains. Correspondingly, TGA reveals an earlier onset of mass loss (~220-250 °C), followed by the main decomposition process up to ~430 °C, suggesting that the presence of CA modifies the thermal decomposition pathway. PAA-LiOH shows a distinct two-step decomposition behavior. The first mass loss stage occurs from ~250 to 450 °C, followed by a second decomposition step at ~620 to 720 °C, likely associated with the breakdown of lithium carboxylate species and residual inorganic lithium-containing phases. Compared with PAA and PAA-CA, the broader decomposition window and delayed high-temperature mass-loss step indicate improved thermal persistence after LiOH neutralization. Overall, these results suggest that CA promotes thermally induced network formation, whereas LiOH modification stabilizes the binder through formation of lithium carboxylate structures with distinct multi-step decomposition behavior.

TGA-DSC was performed on CMC and its chemically modified variants to evaluate the effects of polymer blending, crosslinking, and lithium coordination on thermal stability and decomposition pathways (**Supplementary Fig. 2b, e**). Pristine CMC exhibits an initial minor weight loss below ~150 °C, accompanied by a broad DSC thermal event, which is attributed to the removal of physically adsorbed and bound water associated with the hydrophilic polysaccharide backbone. A major mass loss step is observed between ~250-350 °C, where the weight decreases sharply from ~90% to ~50%, corresponding to the primary decomposition of the CMC backbone through glycosidic bond cleavage, dehydration, and decarboxylation reactions. This process is consistent

with the pronounced DSC peak centered at ~300 °C. At higher temperatures (~350-750 °C), a more gradual mass loss is observed, which can be assigned to further decomposition and carbonization of residual polymer fragments. CMC-CA shows a similar overall decomposition profile to CMC but with slightly earlier onset of mass loss and reduced residual mass evolution at elevated temperature, suggesting that the introduction of citric acid modifies intermolecular interactions and may induce partial esterification between hydroxyl and carboxyl groups during heating. Correspondingly, the DSC response shows a broadened and less intense thermal transition around ~280-320 °C, indicating altered decomposition kinetics relative to pristine CMC. For the CMC-PAA system, the major decomposition step shifts to a broader temperature range (~280-450 °C), with TGA showing a more gradual weight-loss process compared with pristine CMC. This behavior is consistent with the formation of an interpenetrated hydrogen-bonded polymer network between CMC hydroxyl groups and PAA carboxyl groups, which increases structural connectivity and broadens the decomposition pathway[2]. The corresponding DSC trace exhibits an extended thermal event spanning ~100-350 °C, supporting stronger interpolymer interactions and a more distributed bond dissociation process[3]. Upon LiOH incorporation, the CMC-PAA-LiOH sample exhibits the broadest decomposition window (~280-500 °C) and the highest mass retention in the intermediate temperature region (~400-650 °C), indicating enhanced thermal robustness of the polymer matrix. This behavior is attributed to the formation of lithium carboxylate coordination sites within the CMC-PAA network, which introduces ionic crosslinking and stabilizes the polymer framework during thermal decomposition. The broadened DSC response and suppressed low-temperature thermal event further support the presence of a more thermally distributed decomposition process in the lithium-coordinated binder system.

The thermal behaviors of PCC binders containing different citric acid (CA) contents (2-20 wt.%) were evaluated by TGA and DSC to examine the influence of crosslinker concentration on thermal decomposition and network evolution (**Supplementary Fig. 2c, f**). All PCC samples exhibit similar multistep decomposition behavior. A minor low-temperature thermal event below ~150 °C is assigned to the removal of residual moisture. This is followed by the first major mass-loss stage at ~220-320 °C, corresponding to the onset of thermally activated condensation reactions and initial decomposition of the polymer network, and a second major decomposition stage at ~320-

450 °C, associated with further breakdown of the crosslinked PAA-CMC framework and cleavage of CA-mediated ester linkages. A third gradual mass-loss stage above ~650 °C is attributed to the decomposition and carbonization of residual fragments. The DSC profiles show that the heat-flow response evolves systematically with CA content, indicating that the crosslinker concentration strongly affects the thermal reaction pathway of the PCC network. Among all samples, the 20% CA binder shows the lowest residual mass at high temperature, indicating that excessive CA does not improve thermal robustness and may instead introduce a larger fraction of thermally labile linkages.

Comment 3): Include a concise table summarizing: R_{SEI} and R_{ct} evolution (initial vs. end-of-life). Discuss potential limitations of DRT peak assignment, especially in porous composite electrodes. Explicitly state whether electrolyte composition and formation protocols were identical across binder systems.

Reply 3): We thank the reviewer for these constructive suggestions. Accordingly, we have made the following revisions to improve clarity and rigor.

First, we have added a concise summary table comparing the evolution of interfacial resistance components, including R_{SEI} and R_{ct}, at the initial state and at end-of-life for the different binder systems (**Supplementary Table 5**). This table provides a clear, side-by-side overview of interfacial evolution and facilitates direct comparison across samples. (**Page 54 of Supporting Information**)

Second, we have expanded the discussion of the limitations of DRT peak assignment, explicitly noting that peak deconvolution in porous composite electrodes is inherently non-unique and that individual DRT features may reflect overlapping contributions from multiple interfacial and transport processes. The revised text emphasizes that DRT analysis is used here as a comparative diagnostic tool rather than as a definitive assignment of discrete physicochemical processes. (**Page 19-20 of Supporting Information**)

Finally, we now explicitly state that the electrolyte composition, cell configuration, and formation protocols were kept identical across all binder systems to ensure that observed differences in impedance evolution arise from binder effects rather than variations in electrolyte chemistry or formation history. (**Page 19 of Main text**)

We believe these revisions improve the transparency and interpretability of the impedance analysis and directly address the reviewer's concerns.

Supplementary Table 5: Evolution of interfacial resistance components extracted from *in situ* EIS analysis.

Binder system	State	R_s (Ω)	R_{ct}(Ω)
**PVDF**	Initial	1.896	224.3
	1st cycle	1.915	143.3
	2nd cycle	1.918	92.99
	End of life	2.51	15.64
**PCC**	Initial	1.806	284.3
	1st cycle	1.926	151.3
	2nd cycle	2.028	27.86
	End of life	0	0.416

Comment 4): Avoid implying direct causality between PVDF and amorphous layer thickness without: Chemical identification (e.g., EELS, XPS depth profiling). Reframe conclusions to emphasize correlation and consistency, not definitive mechanism.

Reply 4): Thank you for your suggestion. We agree that the relationship between binder chemistry and the observed amorphous interphase should not be interpreted as a definitive causal mechanism without chemical identification. To provide a more comprehensive assessment of the interfacial chemistry, we performed depth-resolved XPS analysis on cycled LFP electrodes employing PCC and PVDF binders (**Supplementary Fig. 19**). Compared with EELS, which probes highly localized regions on individual particles, XPS depth profiling allows a more representative evaluation of surface chemistry across the electrode. High-resolution spectra of C 1s (**Supplementary Fig. 19a**), O 1s (**Supplementary Fig. 19b**), F 1s (**Supplementary Fig. 19c**), Li 1s (**Supplementary Fig. 19d**), and P 2p (**Supplementary Fig. 19e**) were collected as a function of Ar^+ sputtering depth (0-3 steps). (**Pages 29-31 of Supporting Information**)

The C 1s spectra (**Supplementary Fig. 19a**) show that oxygenated carbon species at the PCC electrode surface attenuate rapidly with sputtering, whereas the PVDF electrode exhibits a persistent $-CF_2-$ component (~291 eV), indicating fluorinated polymer-derived residues extending into the near-surface region. Consistently, the O 1s spectra (**Supplementary Fig. 19b**) reveal faster decay of high-binding-energy oxygenated interphase components for PCC, while PVDF retains a thicker oxygenated interphase to greater depths.

The F 1s spectra (**Supplementary Fig. 19c**) further demonstrate that fluorine in the PCC electrode is largely surface-localized and readily removed, whereas the PVDF electrode shows persistent organic fluorine (~688-690 eV) and a LiF-like component (~685 eV) that remains significant, and

can increase at subsurface depths. Correspondingly, the Li 1s spectra (**Supplementary Fig. 19d**) indicate that interphase-related Li species (LiF/carbonate-related) diminish rapidly for PCC but remain pronounced for PVDF, consistent with a salt-rich subsurface interphase. In contrast, the P 2p spectra (**Supplementary Fig. 19e**) remain essentially unchanged across sputtering depth and binder systems, indicating that the phosphate (PO_4^{3-}) units of the $LiFePO_4$ structure are preserved.

Taken together, these depth-dependent trends, corroborated across multiple core levels, demonstrate that PCC forms a thinner, more readily removable interphase, whereas PVDF generates a fluorine- and lithium-rich subsurface interphase. Importantly, these differences arise from interfacial chemistry rather than degradation of the LFP host lattice. (**Pages 10-11 of Main text**)

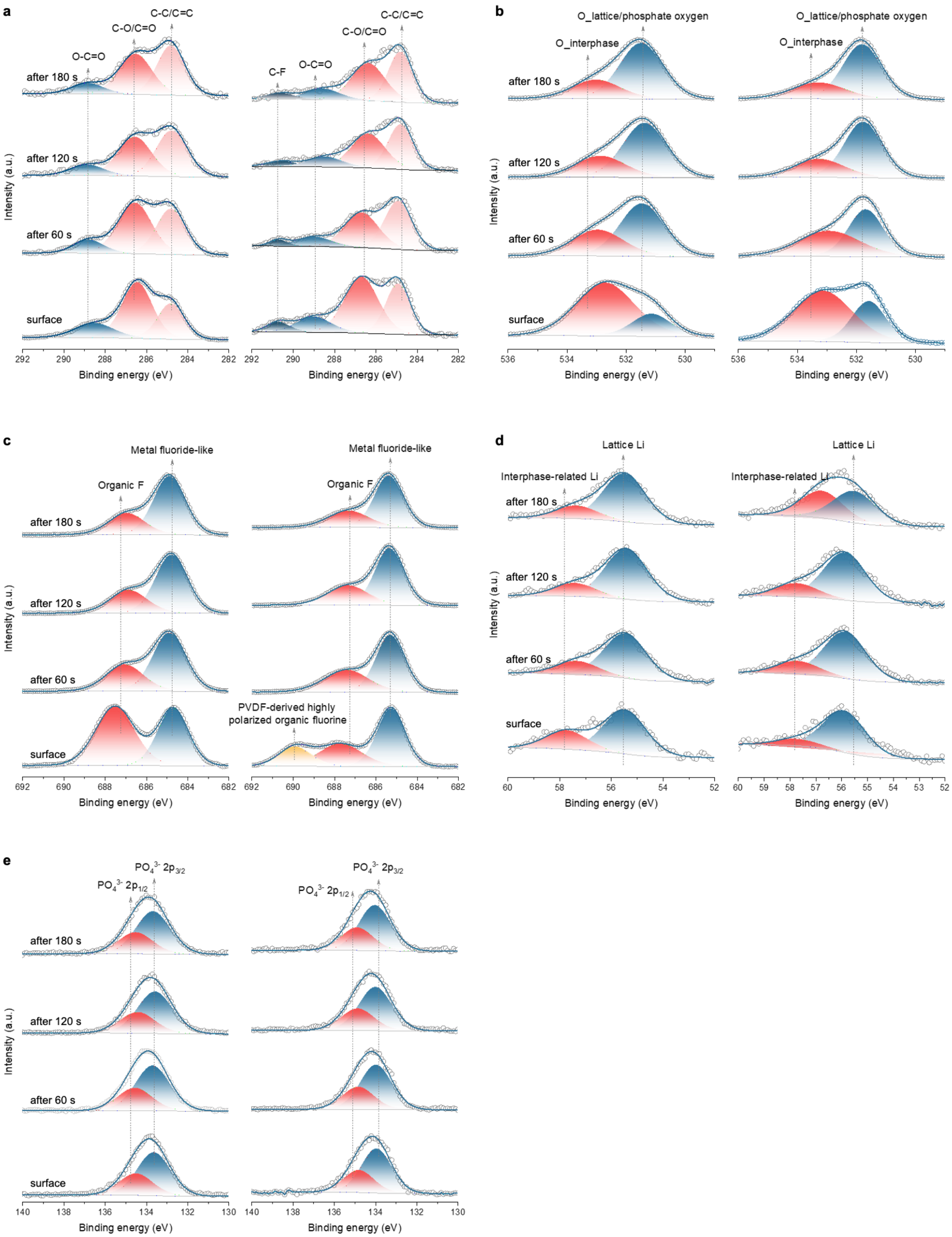
a
C-C/C=C
C-O/C=O
O-C=O
C-F
after 180 s
after 120 s
after 60 s
surface
Intensity (a.u.)
Binding energy (eV)
b
O_lattice/phosphate oxygen
O_interphase
c
Metal fluoride-like
Organic F
PVDF-derived highly
polarized organic fluorine
d
Lattice Li
Interphase-related Li
e
PO_4^{3-} $2p_{3/2}$
PO_4^{3-} $2p_{1/2}$

Supplementary Figure 19: Depth-resolved XPS analysis of cycled LFP electrodes with PCC and PVDF binders. High-resolution XPS spectra of (**a**) C 1s, (**b**) O 1s, (**c**) F 1s, (**d)** Li 1s, and (**e**) P 2p collected from cycled LFP electrodes employing PCC (left) and PVDF (right) binders. For each panel, spectra from bottom to top correspond to increasing sputtering time, including the surface spectrum (0 s) followed by sequential Ar^{+}_{1000} gas cluster sputtering steps of 60 s per cycle (up to three cycles, total 180 s). Binding energies were calibrated using the C-C component of the C 1s peak at 284.8 eV, and spectra were fitted using physically constrained peak positions and full widths at half maximum across sputtering depths. Chemical-state assignments are discussed conservatively using grouped components, particularly for Li 1s and O 1s, due to intrinsic peak overlap and possible sputtering-induced artifacts[4]. Depth-dependent trends are interpreted qualitatively and corroborated across complementary core levels.

.

Comment 5): Some assumptions (e.g., NMP recovery rate, fluorine volatilization fraction, binder removal efficiency) strongly influence results. These assumptions are sometimes presented as fixed values rather than ranges. Recommendations: Explicitly include: Sensitivity analysis or uncertainty ranges Discussion of regional or technological variability Clarify that the LCA reflects a scenario analysis, not a universal industrial reality.

Reply 5): We thank the reviewer for this important comment. We agree that several parameters in the life-cycle assessment, including NMP recovery efficiency, fluorinated species formation and capture, and binder removal effectiveness, can significantly influence quantitative outcomes and should not be interpreted as universally fixed values.

Accordingly, we have revised the manuscript to explicitly clarify that the LCA presented in this work is a scenario-based analysis intended to enable relative comparison between PVDF-NMP and PCC-water binder systems, rather than to represent a universal industrial reality. We now explicitly discuss regional and technological variability, noting that these parameters may vary depending on the regulatory environment, process design, scale, and technology maturity. (**Page 15 of Main text**)

Where appropriate, assumptions that were previously stated as fixed values are now described as representative ranges or benchmark assumptions based on literature and established LCA models. In addition, we have added a dedicated discussion addressing uncertainty and sensitivity, clarifying that while variations in these parameters may affect absolute values of energy use, emissions, and

cost, they do not alter the qualitative conclusions regarding the comparative sustainability advantages of the PCC system. (**Page 17 of Main text**)

Page 15 of Main text

Binder chemistry exerts a profound yet often underestimated influence on the sustainability of LIBs. To elucidate its system-level impact, we conducted a cradle-to-gate comparison between conventional PVDF-NMP and our F-free, water-processable PCC systems. Prospective life cycle assessments (LCAs), based on GREET 2024[5], BatPac 2024[6], and EverBatt 2023[7], quantified environmental and economic burdens from raw-material acquisition through cell manufacturing and EoL processing. For EoL evaluation, solid-state relithiation and graphite reconstruction were benchmarked against conventional high-temperature sintering, while pyrometallurgical and hydrometallurgical pathways were excluded due to their limited suitability for LFP and graphite recovery (**Supplementary Notes 2, 3**)[8]. It is important to note that the LCA presented here represents a scenario-based analysis designed to enable relative comparison between PVDF-NMP and PCC-water binder systems, rather than a universal depiction of industrial practice. Several parameters, including solvent recovery efficiency, fluorinated species formation and capture, and binder removal effectiveness, can vary with regional regulations, plant design, process scale, and technology maturity. Accordingly, the results should be interpreted as indicative of comparative trends rather than absolute industrial benchmarks.

Page 17 of Main text

Across twelve sustainability indicators, including six quantitative (energy consumption, GHG emissions, water usage, cost, solvent consumption, and material wastage) and six qualitative (toxicity index, recyclability, processing simplicity, solvent compatibility, safety, and energy demand), the PCC system outperforms PVDF across all criteria (**Fig. 5g**). Moreover, its ambient aqueous processing and F-free chemistry simplify EoL treatment and mitigate hazards (**Fig. 5h**). While absolute values are influenced by assumptions regarding solvent recovery, fluorine capture, and binder removal efficiency, reasonable variations in these parameters do not alter the qualitative conclusion that replacing PVDF-NMP with a fluorine-free, water-processable binder substantially

reduces solvent-related emissions, fluorine-associated environmental burdens, and process complexity. Future work may further refine these estimates as aqueous manufacturing and direct-recycling technologies continue to mature.

Comment 6): Minor Comments
o Clearly define PCC early and use consistently.
o Avoid switching between "PCC", "PC + CA + LiOH", etc., without reminders.
o A few passages use promotional phrasing ("superior", "dramatic", "decisive").
o Recommend adopting a more neutral academic tone in these sections.

Reply 6): We thank the reviewer for these helpful editorial suggestions. We agree that clarity and consistency in terminology, as well as a neutral academic tone, are important for improving readability and rigor.

Accordingly, we have revised the manuscript to define PCC clearly at its first occurrence as the citric-acid-crosslinked PAA-CMC binder system and to use "PCC" consistently throughout the text. Instances where alternative descriptions such as "PC + CA + LiOH" were previously used have been either replaced with "PCC" or accompanied by brief reminders to avoid ambiguity.

In addition, we have carefully reviewed the manuscript to remove or soften promotional language, replacing terms such as "superior", "dramatic", and "decisive" with more neutral, descriptive phrasing that reflects the data-driven comparisons presented. These revisions ensure a more balanced and objective academic tone while preserving scientific content and conclusions.

Point-by-point response to Reviewer 4's comments

Overall Comments: This manuscript presents a comprehensive narrative that bridges molecular binder design to system-level life-cycle assessment (LCA), offering a clear solution to the critical recycling challenges associated with PVDF. I commend the high completeness of the work—particularly the demonstration of impurity-free recycling—and would like to highlight both the aspects I found especially impressive and the areas that would benefit from further refinement. However, to fully justify publication in Nature Communications, critical issues regarding the chemical novelty and processability on Ni-rich cathodes must be addressed.

Reply: We thank the reviewer for the thoughtful and balanced assessment of our manuscript. We appreciate the recognition of the integrated narrative linking molecular binder design to system-

level life-cycle assessment, as well as the positive evaluation of the impurity-free direct recycling demonstration.

We also acknowledge the reviewer's concern that, to fully justify publication in *Nature Communications*, the chemical novelty and processability of the binder system on Ni-rich cathodes require clearer justification. In response, we have revised the manuscript to more explicitly articulate the chemical design rationale and novelty of the PCC binder relative to prior aqueous binder systems, clarifying how the coupled covalent, ionic, and hydrogen-bonded network differentiates PCC from previously reported formulations. In addition, we have strengthened the discussion of processability and electrochemical behavior on Ni-rich cathodes.

Comment 1): Stability on NCM811. To substantiate the claim of a "universal platform" for Ni-rich cathodes, the authors should address the risk of slurry gelation. Please provide time-dependent viscosity data (e.g., 0 h, 6 h, 24 h) for the NCM811–PCC slurry to demonstrate stability during storage and casting.

Reply 1): We thank the reviewer for raising the important concern regarding slurry stability and potential gelation for Ni-rich cathodes. In response, we have performed time-dependent rheological measurements on NCM811 slurries formulated with the PCC binder at holding time of 0, 6, and 24 h under identical formulation and measurement conditions (liquid-to-solid ratio L/S = 1.5; carbon additive omitted to isolate binder-NCM interactions). As shown in **Supplementary Figure 22a**, the NCM811-PCC slurry exhibits no abrupt viscosity increase or shear-rate-independent plateau over time, which are characteristic signatures of slurry gelation. Instead, only a modest increase in low-shear viscosity is observed with holding time, while the magnitude of overall viscosity and shear-thinning behavior remain largely unchanged. This indicates a stable particle-binder network without time-dependent gelation during storage or casting-relevant time scales.

For comparison, time-dependent data for PVDF-based NCM811 slurries are also included, which show a pronounced viscosity decrease with holding time. Consistent with the rheological trends, visual inspection after 48 h of static holding (reveals significant sedimentation in the PVDF slurry, whereas the PCC-based slurry remains homogeneous **Supplementary Fig. 22b, c**).

Collectively, these results demonstrate that the PCC binder provides robust time-dependent stability without gelation for NCM811 slurries, supporting its applicability as a universal aqueous binder platform for Ni-rich cathodes during practical slurry storage and electrode fabrication. (**Page 11 of Main text**; **Pages 34-35 of Supporting Information**)

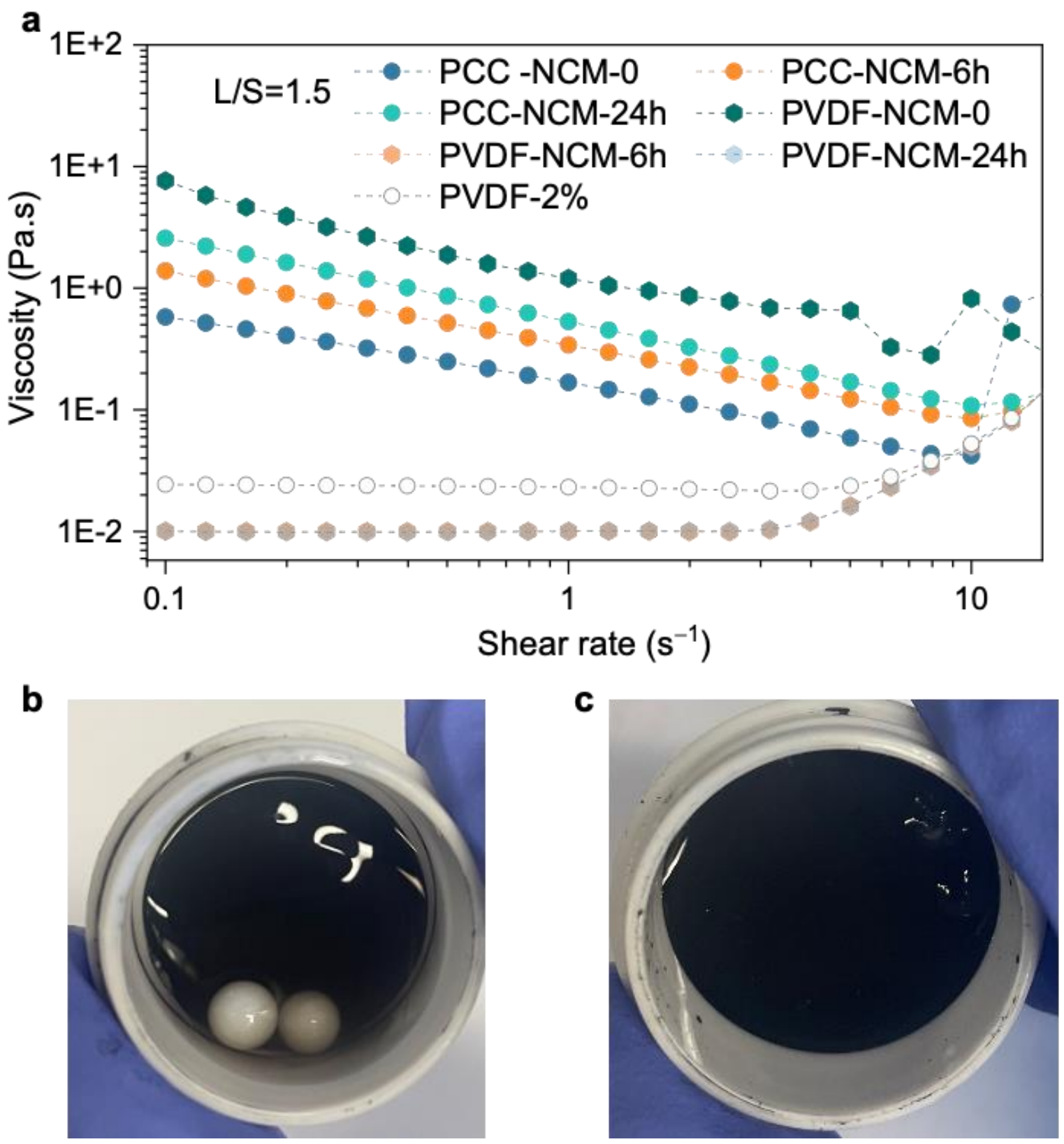

Supplementary Figure 22: Time-dependent viscosity and dispersion behavior of NCM811 slurries. a, Viscosity evolution of NCM811 electrode slurries prepared with PCC and PVDF binders as a function of holding time (0, 6, and 24 h), measured under identical formulation conditions (liquid-to-solid ratio, L/S = 1.5; carbon additive omitted to isolate binder-NCM interactions) and identical rheological measurement settings. **b, c,** Photographs of PVDF-based (b) and PCC-based (c) NCM811 slurries after 48 h of static holding. The PVDF-based slurry precipitates, leaving a clear solution on the top where the balls can be clearly seen while the PCC slurry remains homogenous without phase separation.

Comment 2): Verification of chemical novelty. To prove the necessity of the specific "esterification crosslinking" chemistry beyond what can be achieved with conventional PAA/CMC-type binders, please include a head-to-head performance comparison against a simple physical mixture (PAA/CMC/CA without esterification).

Reply 2): We thank the reviewer for this important comment regarding verification of chemical novelty. We agree that distinguishing the performance contribution of esterification-enabled crosslinking from that of a simple physical mixture is essential to justify the necessity of the PCC chemistry.

In response, we have included a direct head-to-head electrochemical comparison between the fully crosslinked PCC binder, and a control binder formulated as a physical mixture of PAA/CMC/CA without esterification (prepared under conditions that suppress covalent crosslinking). As shown in **Fig. 2g-j**, electrodes prepared with the physically mixed binder exhibit severe cracking or particle aggregation in the freshly fabricated electrode sheets, indicating insufficient mechanical integrity during electrode formation. For comparison, systems containing only partially crosslinked components, such as CMC-CA and PAA-LiOH-CA (**Supplementary Fig. 6**), also show significant delamination, further highlighting the limitations of single-component crosslinking strategies. In contrast, electrodes prepared with the PCC binder form continuous and mechanically robust films. Consistent with these observations, the physically mixed binder also exhibits inferior electrochemical performance and accelerated capacity decay compared with PCC (**Supplementary Figure 3a**). (**Pages 8-10 of Supporting Information**)

These results demonstrate that the observed performance improvements cannot be attributed solely to hydrogen bonding or physical blending of PAA, CMC, and CA. Instead, the esterification-induced covalent network formation plays a critical role in stabilizing electrode structure and interfacial integrity during prolonged cycling. We have revised the manuscript to explicitly clarify this distinction and to emphasize that the chemical novelty of PCC lies in the formation of a hybrid covalent-ionic-hydrogen-bonded network, rather than in the mere combination of known binder components.

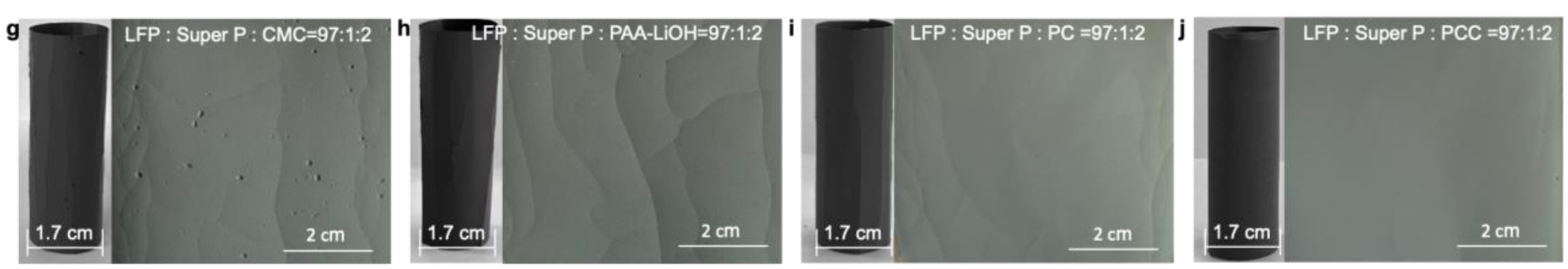

Fig. 2: Characterizations of the PCC binder system. **g-j,** Digital photographs of LFP electrodes prepared with different binders: **(g)** CMC, **(h)** PAA-LiOH, **(i)** PC, and **(j)** PCC. All electrodes were prepared with a fixed composition of LFP: Super P: Binder = 97: 1: 2 by weight and cast on carbon-coated Al foil under identical conditions.

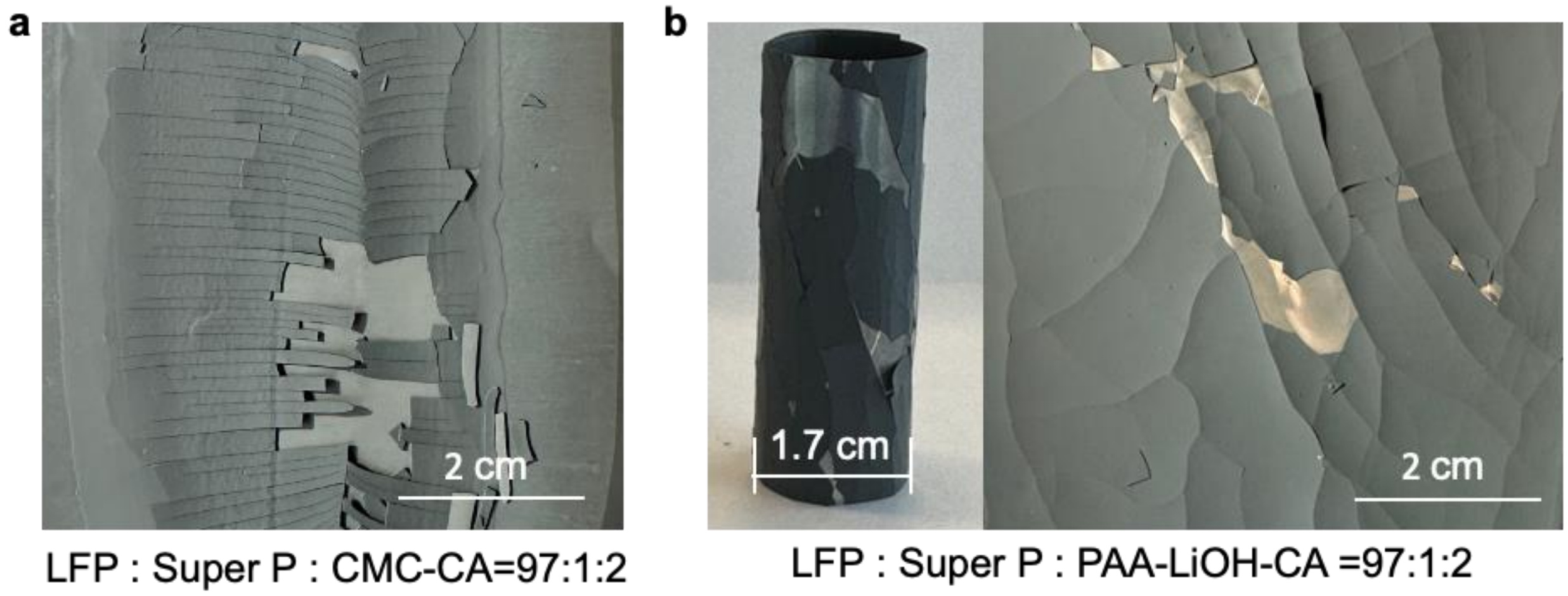

Supplementary Figure 6: **Photographs and corresponding planar views of LFP electrodes using different binder systems**. **a**, CMC-CA, **b**, PAA-LiOH-CA. All electrodes were prepared with a fixed composition of LFP: Super P: Binder = 97: 1 : 2 by weight and cast on carbon-coated Al foil under identical conditions.

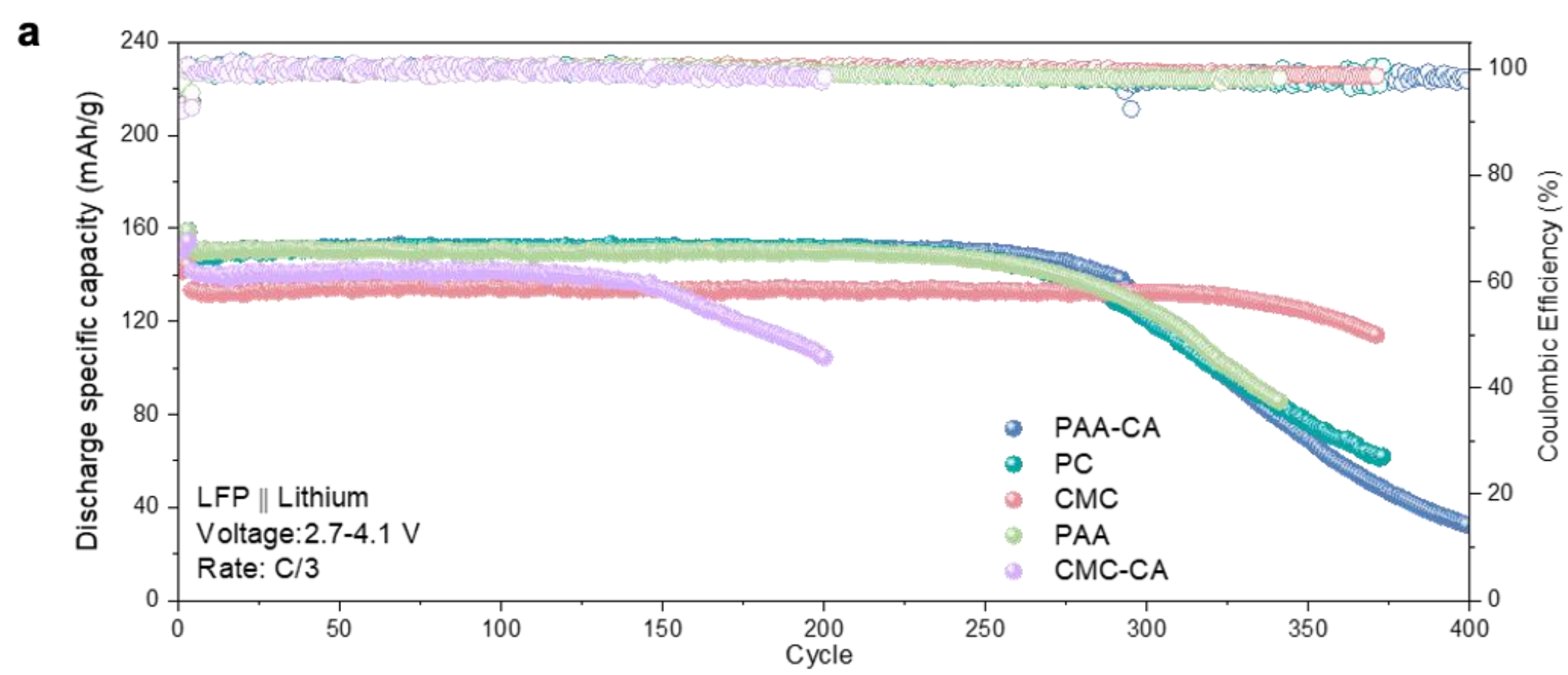

Supplementary Figure 3: Effect of citric acid (CA) content on PCC binder properties and electrochemical performance. a, Electrochemical performance comparison of PAA/CMC binders with and without esterification crosslinking. Cycling stability of electrodes prepared with PAA-CA, PAA-CMC-LiOH (PC), CMC, CMC-CA, and PAA-LiOH binders. The comparison isolates the effect of esterification-enabled covalent crosslinking from physical blending and hydrogen-bond interactions, highlighting its role in stabilizing electrochemical performance during prolonged cycling.

Comment 3): Durability at high temperature and voltage. LSV data alone is insufficient for practical validation of oxidative stability. Please add leakage-current measurements (floating test) or cycling data at elevated temperature (>45 °C) and high cut-off voltage (4.5 V) to confirm stability under harsh operating conditions.

Reply 3): We thank the reviewer for this constructive suggestion. We agree that LSV alone is insufficient to validate oxidative stability under practical operating conditions.

To address this concern, we have added floating (leakage-current) measurements for the LFP-PCC system. As shown in **Fig. 3a**, the PCC-based LFP electrode exhibits a near-zero leakage current during a 100 h floating test at 4.1 V, indicating effective interfacial passivation and suppressed parasitic oxidation under high-voltage hold conditions. (**Pages 8-9 of Main text**)

In addition, we have included cycling tests at elevated temperature and high cut-off voltage in the LMFP system. LFP is not suitable for cycling to 4.5 V because its redox plateau (~3.45 V vs Li/Li^+) lies far below this voltage window. Therefore, LMFP serves as a more appropriate platform for evaluating binder stability under high-voltage conditions. As shown in **Supplementary Fig. 21a**, the LMFP-PCC electrode exhibits stable charge-discharge voltage profiles at 42 °C (our standardized elevated-temperature protocol for accelerated stability evaluation) up to 4.5 V, with well-maintained plateaus and minimal polarization growth, indicating preserved reaction kinetics under thermal and high-voltage stress. Correspondingly, the cycling performance demonstrates that the LMFP-PCC electrode delivers an initial discharge capacity of 144.0 mAh g^{-1} at C/3 and retains 91.2% of its capacity after 180 cycles (**Supplementary Fig. 21b**). These results confirm the electrochemical stability of the PCC binder under combined high-voltage and elevated-temperature conditions, thereby providing practical validation of its durability in harsh operating environments. (**Page 11 of Main text**; **Page 33 of Supporting Information**)

The manuscript has been revised accordingly.

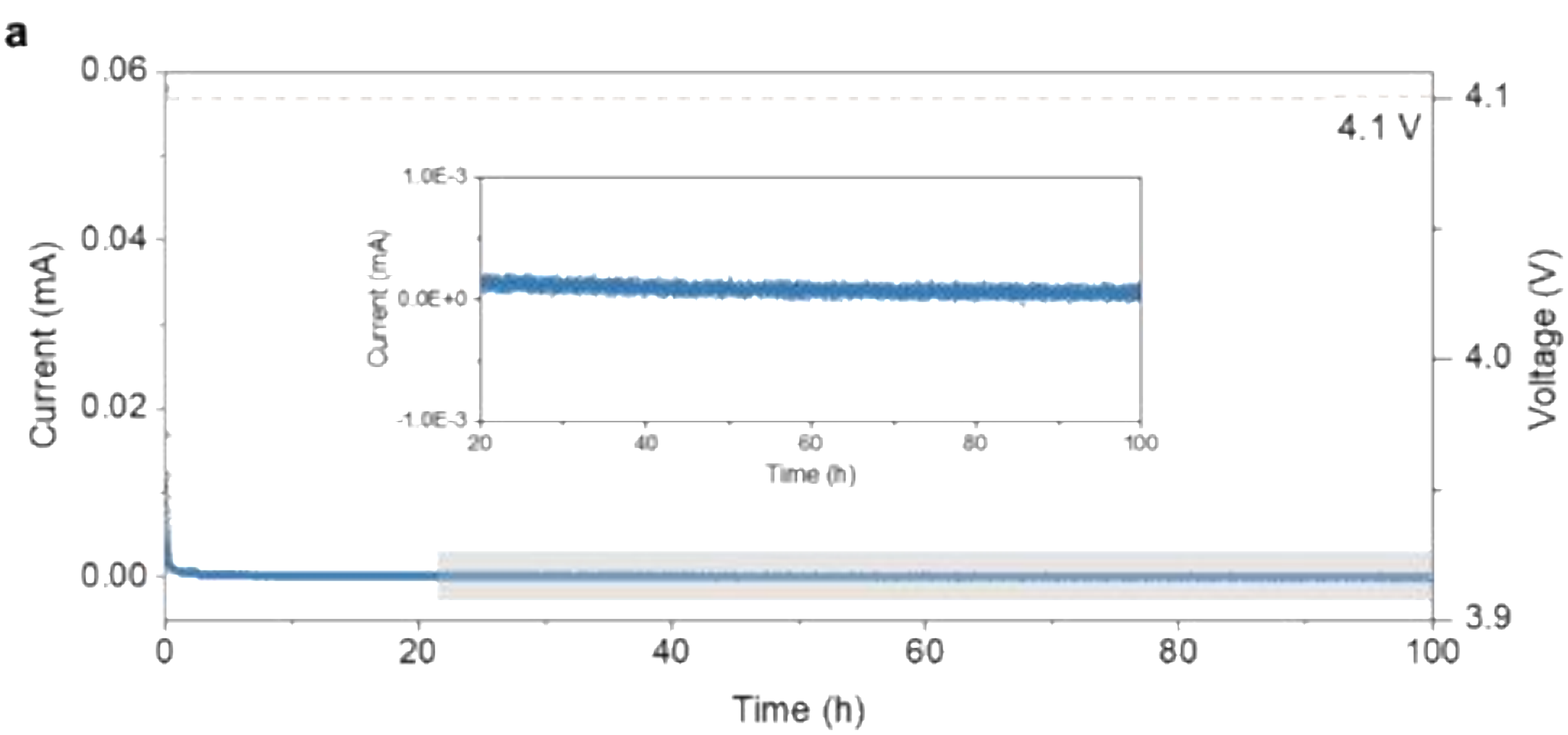

Fig. 3: PCC binder enables uniform coatings, strong adhesion and stable electrochemical performance. a, Current profiles of LFP||Lithium half-cells under 4.1 V float charging test at 25 ℃.

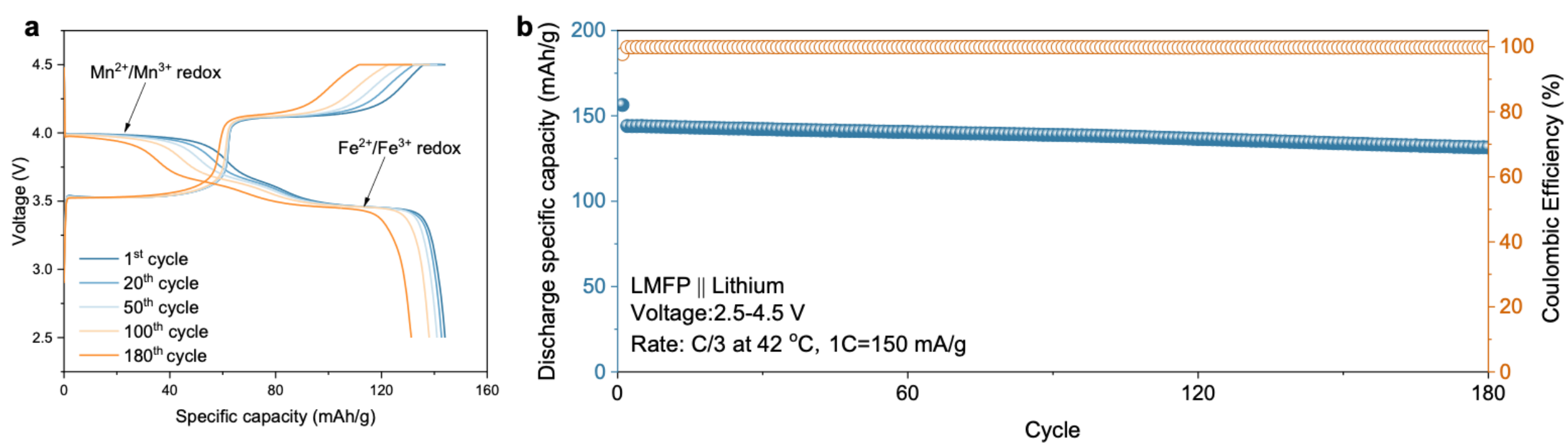

Supplementary Figure 21: Cycling performance of LMFP at 42 °C. a, Representative charge-discharge profiles and **b**, cycling stability of LMFP at 42 °C, evaluated at C/3 with a voltage window of 2.5-4.5 V vs. Li/Li^+.

References:

1 Xu, P., Dai, Q., Gao, H., Liu, H., Zhang, M., Li, M., Chen, Y., An, K., Meng, Y. S., Liu, P., Li, Y., Spangenberger, J. S., Gaines, L., Lu, J. & Chen, Z. Efficient Direct Recycling of Lithium-Ion Battery Cathodes by Targeted Healing. *Joule* **4**, 2609-2626, (2020).

2 He, Y., Zhu, B. & Inoue, Y. Hydrogen bonds in polymer blends. *Prog. Polym. Sci.* **29**, 1021-1051, (2004).

3 Han, M., Liang, X., Han, C., Zhou, C., Xiang, Q., Cao, Y. & Sun, J. Cross-Linked Binary Polymeric Binder of Polyacrylic Acid–Carboxymethyl Cellulose for Improving Phosphorus Anode. *Adv. Funct. Mater.* **34**, 2308325, (2024).

4 Xu, K. Electrolytes and Interphases in Li-Ion Batteries and Beyond. *Chem. Rev.* **114**, 11503-11618, (2014).

5 Wang, M., Cai, Hao, Lu, Zifeng, Elgowainy, Amgad, Alam, Md Rakibul, Benavides, Pahola T., Benvenutti, Lívia, Burnham, Andrew, Do, Thai N., Farhad, Masum, Gan, Yu, Gracida-Alvarez, Ulises R., Hawkins, Troy R., Iyer, Rakesh K., Kar, Saurajyoti, Kelly, Jarod C., Kim, Taemin, Kolodziej, Christopher, Kwon, Hoyoung, Lee, Uisung, Lim, Juin Y., Liu, Xinyu, Morales, Michele, Ng, Clarence, Ou, Longwen, Pandey, Ishan, Shukla, Siddharth, Siddique, Nazib, Sun, Pingping, Sykora, Tom, Vyawahare, Pradeep, and Zhou, Jo. Greenhouse gases,. Greenhouse gases, Regulated Emissions, and Energy use in Technologies Model ® (2024 .Net). Computer Software. *USDOE Office of Energy Efficiency and Renewable Energy (EERE)*, (2024).

6 Kevin W. Knehr, J. J. K., Paul A. Nelson, and Shabbir Ahmed. Battery Performance and Cost Modeling for Electric-Drive Vehicles: A Manual for BatPaC v5.0. *ANL/CSE-22/1, July 2022*, (2022).

7 Dai, Q., Spangenberger, J., Ahmed, S., Gaines, L., Kelly, J. C. & Wang, M. EverBatt: A closed-loop battery recycling cost and environmental impacts model. (Argonne National Laboratory (ANL), Argonne, IL (United States), 2019).

8 Lin, J., Li, W. & Chen, Z. Scaling Direct Recycling of Lithium-Ion Batteries toward Industrialization: Challenges and Opportunities. *ACS Energy Lett.* **10**, 947-957, (2025).

Point-by-point response to Reviewer 1's comments

Overall Comments: The authors have done a good job of addressing all concerns. This manuscript is acceptable for publication now as is.

Reply: We thank the reviewer for the positive evaluation of our revised manuscript and for recommending its acceptance for publication.

Point-by-point response to Reviewer 2's comments

Overall Comments: I co-reviewed this manuscript with one of the reviewers who provided the listed reports. This is part of the Nature Communications initiative to facilitate training in peer review and to provide appropriate recognition for Early Career Researchers who co-review manuscripts.

Reply: We thank the reviewer for the positive assessment of our manuscript and for the recommendation for publication in Nature Communications following revision. We also appreciate the transparency regarding the co-review process and the initiative to support training and recognition of Early Career Researchers in peer review.

Point-by-point response to Reviewer 4's comments

Overall Comments: The reviewer's comments have been adequately addressed, and the manuscript has been improved accordingly. I therefore recommend acceptance of this manuscript.

Reply: We thank the reviewer for the positive assessment of our revised manuscript and for recommending its acceptance for publication.

Comment): I have reviewed the authors' responses to Reviewer 3's comments. Overall, the authors appear to have adequately addressed the major concerns raised by Reviewer 3. In particular, they provided additional electrochemical data to support the correlation between CA crosslinking and electrochemical performance. They also revised the interpretation of the DSC and DRT results by avoiding overly quantitative or definitive statements and reframing them as qualitative or comparative analyses. In addition, depth-resolved XPS data were added to support the discussion of the PVDF-related interphase, and the LCA section was clarified as a scenario-based comparative analysis. The terminology and overly promotional expressions also appear to have been appropriately revised.

However, I believe that Supplementary Table 5 requires final confirmation. In the authors' response, they state that RSEI and Rct were summarized, whereas the actual table is labeled with Rs and Rct. Therefore, it should be clarified whether Rs was intended to represent RSEI. In addition, the PCC end-of-life Rs value is reported as 0 Ω, which should be checked to determine whether it is a typographical error, a fitting artifact, or a negligible fitted value. In summary, I consider the rebuttal suitable for further consideration. If the minor issue regarding the Rs/RSEI notation and the PCC end-of-life Rs value in Supplementary Table 5 is clarified, I do not see any major remaining problem with the authors' response to Reviewer 3.

Reply 1): We sincerely thank the reviewer for the careful evaluation of our revision and for identifying this inconsistency.

To address this concern, we have re-examined the impedance fitting results and updated the corresponding fitting parameters in **Supplementary Table 5** to ensure consistency between the equivalent-circuit analysis and the impedance spectra presented in the manuscript.

In the previous version, the resistance components resolved by DRT were denoted as solid-electrolyte interphase resistance (R_{SEI}) and charge-transfer resistance (R_{ct}), whereas the corresponding resistance components obtained from equivalent-circuit fitting were denoted as cathode electrolyte interphase resistance (R_{CEI}) and R_{ct}. The simultaneous use of the abbreviations R_{SEI} and R_{CEI} may have led readers to interpret them as representing the same resistance obtained from different analysis methods.

In the revised manuscript, the mid-frequency DRT process is assigned to the cathode electrolyte interphase (CEI) to maintain consistency with the electrode system and equivalent-circuit analyses. However, because DRT resolves relaxation processes according to their characteristic time constants rather than directly yielding equivalent-circuit parameters, we refer to this process descriptively as the cathode electrolyte interphase resistance in the DRT analysis, while reserving the abbreviation R_{CEI} for the resistance parameter obtained from the equivalent-circuit fitting. The manuscript and Supplementary Information have been revised accordingly to clearly distinguish the DRT interpretation from the equivalent-circuit fitting results.

Regarding the PCC end-of-life resistance value noted by the reviewer, we rechecked the corresponding fitting result and corrected the value reported **in Supplementary Table 5**. We thank the reviewer for bringing this inconsistency to our attention, which has helped improve the accuracy and clarity of the impedance analysis.

Supplementary Table 5: Evolution of interfacial resistance components extracted from *in-situ* EIS analysis. The fitting quality is indicated by the normalized error ($\chi^2/|Z|^2$). The equivalent-circuit fitting was performed using the circuit shown in **Supplementary Fig. 16**, where the interfacial resistance is represented by R_{CEI}. In the present Li||LFP half-cell system, changes in R_{CEI} primarily reflect the evolution of the cathode electrolyte interphase (CEI), as the cathode binder is the only experimental variable.

Binder systems	States	R_s (Ω)	R_{CEI} (Ω)	R_{ct}(Ω)	$\chi^2/|Z|^2$
**PVDF**	Pristine	2.0	15.14	247.9	0.03812
	1st cycle	1.996	20.39	152.3	0.03288
	2nd cycle	2.031	20.37	87.41	0.03215
	End of life (710 cycles)	2.464	6.647	45.01	0.0189
**PCC**	Pristine	1.962	2.099	342.7	0.05341
	1st cycle	1.969	4.52	152.7	0.02448
	2nd cycle	2.164	3.992	30.43	0.03939
	End of life (1000 cycles)	4.927	3.378	3.656	0.02988

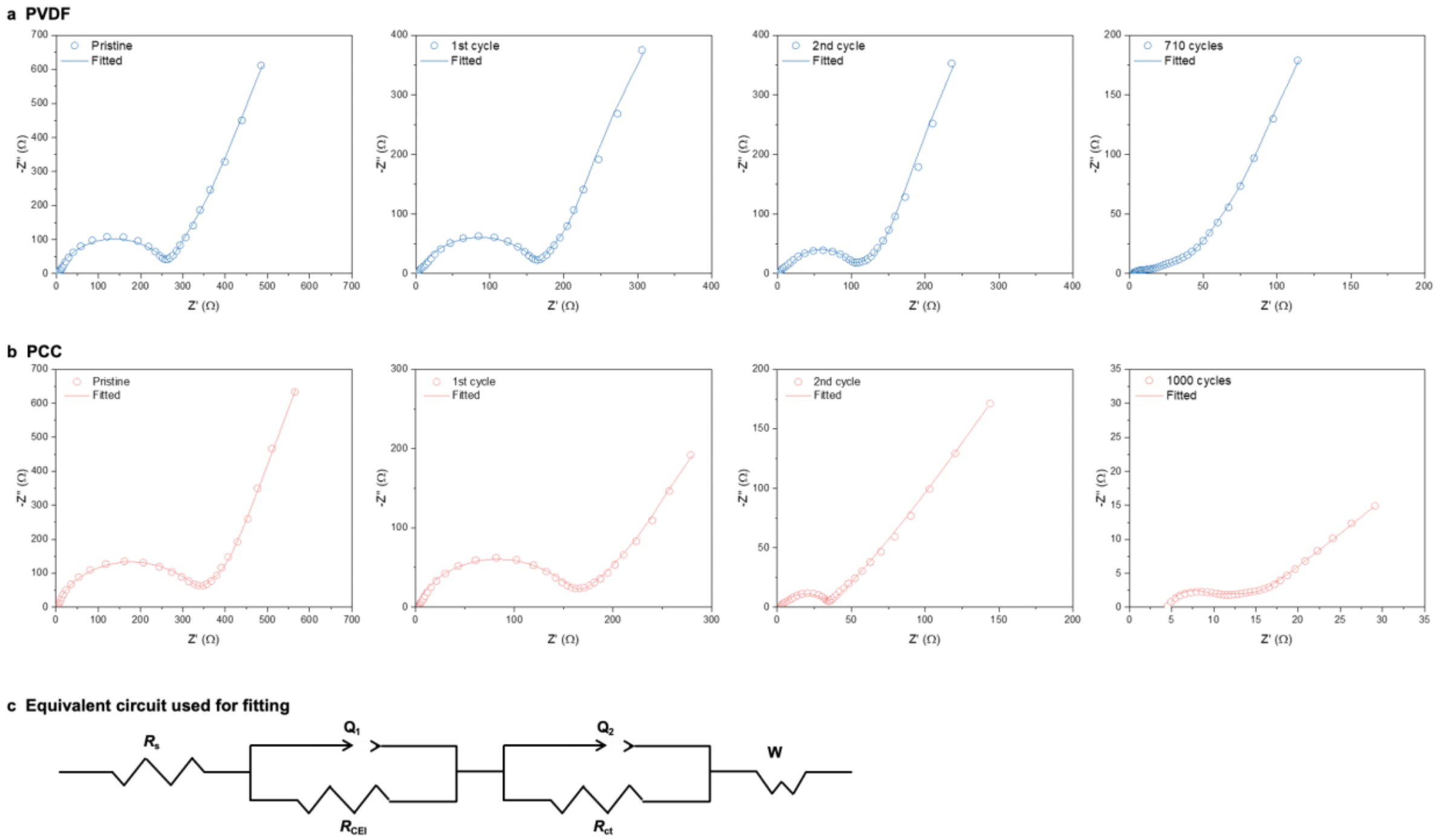

Supplementary Figure 16: Experimental Nyquist plots and corresponding equivalent-circuit fits. Experimental electrochemical impedance spectra (symbols) and corresponding equivalent-circuit fits (solid lines) for **(a)** PVDF and **(b)** PCC electrodes measured in the pristine state, after cycles 1 and 2, and at the end of life (left to right). **(c)** Equivalent circuit used for impedance fitting. The extracted fitting parameters are summarized in Supplementary Table 5.
